# Terpenoids from Kiwi endophytic fungus *Bipolaris* sp. and their antibacterial activity against *Pseudomonas syringae* pv. *actinidiae*

**DOI:** 10.3389/fchem.2022.990734

**Published:** 2022-09-01

**Authors:** Jun-Jie Yu, Wen-Ke Wei, Yu Zhang, Russell J. Cox, Juan He, Ji-Kai Liu, Tao Feng

**Affiliations:** ^1^ School of Pharmaceutical Sciences, South-Central Minzu University, Wuhan, China; ^2^ State Key Laboratory of Phytochemistry and Plant Resources in West China, Kunming Institute of Botany, Chinese Academy of Sciences, Kunming, China; ^3^ Institute for Organic Chemistry and Biomolekulares Wirkstoffzentrum (BMWZ), Hannover, Germany

**Keywords:** endophytic fungus, *Bipolaris* sp., sesquiterpenoids, sesterterpenoids, antibacterial activity

## Abstract

A chemical investigation on the kiwi endophytic fungus *Bipolaris* sp. Resulted in the isolation of eight new terpenoids (**1**–**8**) and five known analogues (**9**–**13**). Compounds **1**–**5** are novel sativene sesquiterpenoids containing three additional skeletal carbons, while compounds **4** and **5** are rare dimers. Compounds **6**–**8** and **13** are sesterterpenoids that have been identified from this species for the first time. Compounds **4** and **5** showed antibacterial activity against kiwifruit canker pathogen *Pseudomonas syringae* pv. *Actinidiae* (Psa) with MIC values of 32 and 64 *μ*g/ml, respectively.

## 1 Introduction

Kiwifruit is an important global food source produced at a scale of 4 million tons per year (Richardson et al., 2018; Dolly et al., 2021). However, the kiwi plant (*Actinidia chinensis* Planch.) is severely attacked by canker caused by the pathogenic bacterium *Pseudomonas syringae* pv. *Actinidiae* (Psa) (Renzi et al., 2012; Scortichini et al., 2012). As one of the major countries in the kiwifruit industry, China’s kiwifruit has also suffered extensive damage from canker disease, causing huge economic losses (Serizawa et al., 1989; McCann et al., 2017; Vanneste, 2017). Traditional Psa inhibitors such as copper-based preparations and streptomycin are not friendly to the environment and even cause drug resistance (Bardas et al., 2010; Colombi et al., 2017; Scortichini, 2018; Wicaksono et al., 2018). Therefore, the development of new antibacterial agents is highly desireable.

**FIGURE 1:**
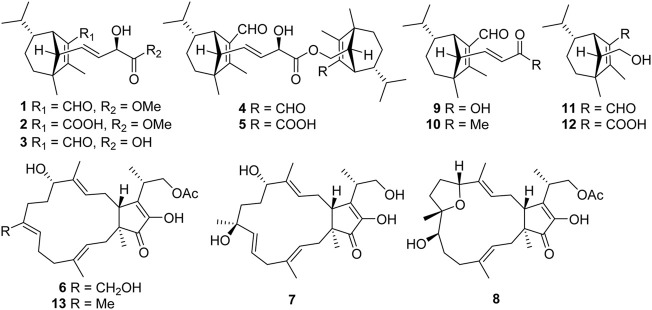
Structures of compounds **1**–**13**.

Endophytes and hosts have formed a close interrelationship in the long-term evolution process, making endophytes an excellent resource for the production of natural antibacterial ingredients (Kusari et al., 2012; Gouda et al., 2016; Gupta et al., 2020). Our strategy intends to explore the active substances against Psa from the metabolites of the endophytic bacteria of the kiwifruit itself. Some progress has been made in our previous research. For example, 3-decalinoyltetramic acids and cytochalasins from the kiwifruit endophytic fungus *Zopfiella* sp. Showed anti-Psa activity (Yi et al., 2021; Zhang et al., 2021), while imidazole alkaloids from *Fusarium tricinctum* were characterized as anti-Psa agents (Ma et al., 2022). *Bipolaris* sp. Is also an endophytic fungus that was characterized from health kiwi plant. Our previous chemical investigation on this fungus yielded a series sesquiterpenoids (bipolarisorokins A–I) and xanthones with anti-Psa properties from the liquid fermented extract (Yu et al., 2022). In order to search for more anti-Psa agents from this fungus, we further investigated the fermentation products from the culture grown on rice medium. As a result, eight new terpenoids including five sesquiterpenoids (**1**–**5**) and three new sesterterpenoids (**6**–**8**), as well as five known analogues (**9**–**13**), have been obtained (Figure 1). Their structures have been identified by extensive spectroscopic methods, as well as quantum chemical calculations. All compounds were evaluated for their anti-Psa activity. Herein, the isolation, structure elucidation and anti-Psa activity of these isolates are reported.

## 2 Experimental section

### 2.1 General experimental procedures

Optical rotations were measured with an Autopol IV polarimeter (Rudolph, Hackettstown, NJ, United States ). UV spectra were obtained using a double beam spectrophotometer UH5300 (Hitachi High-Technologies, Tokyo, Japan). 1D and 2D NMR spectra were run on a Bruker Avance III 600 MHz spectrometer with TMS as an internal standard. Chemical shifts (*δ*) were expressed in ppm with references to the solvent signals. High resolution electrospray ionization mass spectra (HR-ESIMS) were recorded on a LC-MS system consisting of a Q Exactive™ Orbitrap mass spectrometer with an HRESI ion source (ThermoFisher Scientific, Bremen, Germany) used in ultra-high-resolution mode (140,000 at m/z 200) and a UPLC system (Dionex UltiMate 3000 RSLC, ThermoFisher Scientific, Bremen, Germany). Column chromatography (CC) was performed on silica gel (200–300 mesh, Qingdao Marine Chemical Ltd., Qingdao, China), RP-18 gel (20–45 μm, Fuji Silysia Chemical Ltd., Kasugai, Japan), and Sephadex LH-20 (Pharmacia Fine Chemical Co., Ltd., Sweden). Medium-pressure liquid chromatography (MPLC) was performed on a Büchi Sepacore System equipped with a pump manager C-615, pump modules C-605, and a fraction collector C-660 (Büchi Labortechnik AG, Flawil, Switzerland). Preparative high-performance liquid chromatography (prep-HPLC) was performed on an Agilent 1,260 liquid chromatography system equipped with Zorbax SB-C18 columns (5 μm, 9.4 mm × 150 mm, or 21.2 mm × 150 mm) and a DAD detector. Fractions were monitored by TLC (GF 254, Qingdao Haiyang Chemical Co., Ltd., Qingdao, China), and spots were visualized by heating silica gel plates sprayed with 10% H_2_SO_4_ in EtOH.

### 2.2 Fungal material

The fungus *Bipolaris* sp. Was isolated from fresh and healthy stems of kiwifruit plants (*Actinidia chinensis* Planch, Actinidiaceae), which were collected from the Cangxi county of the Sichuan Province (GPS: N 31°12′, E 105°76′) in July 2018. Each fungus was obtained simultaneously from at least three different healthy tissues. The strain was identified as one species of the genus *Bipolaris* by observing the morphological characteristics and analysis of the internal transcribed spacer (ITS) regions. A living culture (internal number HFG-20180727-HJ32) has been deposited at the School of Pharmaceutical Sciences, South-Central Minzu University, China.

### 2.3 Fermentation, extraction, and isolation

The fungus *Bipolaris* sp. Was cultured on potato dextrose agar (PDA) medium for 7 days, which was used as “seed” to incubate in rice medium. The 500 ml Erlenmeyer flasks containing 100 g rice and 80 ml distilled water in each were sterilized at 120°C for 15 min. Then the pieces of *Bipolaris* sp. PDA medium was inoculated into Erlenmeyer flasks. A total of a hundred 500 ml Erlenmeyer flasks were incubated statically in dark place at 25°C for 28 days.

The cultures of *Bipolaris* sp. Were extracted with 5 L methanol four times, and the total residue was obtained by reduced pressure evaporation. Then, the remaining aqueous phase was further extracted four times by EtOAc to afford a crude extract (45.0 g). The latter was subjected to silica gel CC (200–300 mesh) eluted with a gradient of CHCl_3_-MeOH (from 1:0 to 0:1, *v/v*) to obtain five fractions, A–E. Fraction B was separated by CC over silica gel with a gradient elution of the CHCl_3_-MeOH system (from 15:1 to 0:1, *v/v*) to give five fractions (Fr. B1–B5). Fraction B2 was applied to Sephadex LH-20 eluting with CHCl_3_-MeOH (1:1, *v/v*) and was separated by HPLC with MeCN-H_2_O (21:79, *v/v*, 4.0 ml/min) to obtain **6** (4.3 mg, retention time (*t*_R_) = 26.3 min), and **13** (5.2 mg, *t*_R_ = 29.4 min). Fraction B3 was subjected to Sephadex LH-20 (MeOH) and then further repeatedly purified by semipreparative HPLC with MeCN-H_2_O (32:68, *v/v*, 3.0 ml/min) to afford **11** (86.2 mg, retention time (*t*_R_) = 24.2 min) and **12** (94.3 mg, *t*_R_ = 27.2 min). Fraction B4 was purified using semipreparative HPLC with MeCN-H_2_O (20:80, *v*/*v*, 4.0 ml/min) to afford **10** (7.8 mg, *t*_R_ = 16.8 min) and **9** (9.6 mg, *t*_R_ = 20.6 min). Fraction C was purified by semipreparative HPLC with MeCN-H_2_O (26:74, *v/v*, 4.0 ml/min) to afford **7** (4.8 mg, *t*_R_ = 24.6 min) and **8** (7.3 mg, *t*_R_ = 27.5 min). Fraction D was separated by CC over silica gel with a gradient elution of PE-acetone (from 50:1 to 0:1, *v/v*), and then was purified by semipreparative HPLC with MeCN-H_2_O (18:82, *v/v*, 4.0 ml/min) to obtain **1** (6.4 mg, *t*_R_ = 29.6 min), **2** (3.8 mg, *t*_R_ = 24.3 min), and **3** (7.6 mg, *t*_R_ = 18.5 min). Fraction E was purified over Sephadex LH-20 eluted with MeOH, and was further separated using semipreparative HPLC with MeOH-H_2_O (78:22, *v/v*, 3.0 ml/min) to afford **4** (8.7 mg, *t*_R_ = 38.2 min) and **5** (12.8 mg, *t*_R_ = 34.3 min).

Bipolarisorokin J (**1**): colorless oil; ${\mathrm{[\alpha ]}}_{D}^{20}$ – 99.8 (*c* 0.05, MeOH); UV (MeOH) *λ*_max_ (log *ε*) 260 (3.86); ^1^H NMR (600 MHz, CDCl_3_) and ^13^C NMR (150 MHz, CDCl_3_) data, see Table 1; positive ion HR-ESI-MS *m/z* 343.18790, [M + Na]^+^, (calculated for C_19_H_28_NaO_4_^+^, 343.18798).

**TABLE 1:** ^1^H and ^13^C NMR data for **1**–**3**.

No.	1*^a^*		2*^b^*		3*^b^*	
	*δ*_C_, type	*δ*_H_ (*J* in Hz)	*δ*_C_, type	*δ*_H_ (*J* in Hz)	*δ*_C_, type	*δ*_H_ (*J* in Hz)
1	137.8, C		128.2, C		138.8, C	
2	165.9, C		160.0, C		169.1, C	
3	52.4, C		52.8, C		53.6, C	
4a	33.8, CH_2_	1.43, d (5.9)	34.5, CH_2_	1.33, ddd (13.2, 11.9, 5.8)	34.6, CH_2_	1.51, dd (13.3, 6.3)
4b		1.35, dd (12.4, 5.9)		1.44, m		1.42, td (12.8, 5.9)
5a	25.4, CH_2_	1.75, dd (11.7, 6.6)	26.4, CH_2_	1.76, dt (13.6, 5.2)	26.4, CH_2_	1.78, m
5b		0.87, m		1.00, overlap		0.92, dd (10.3, 6.4)
6	44.4, CH	1.02, overlap	46.2, CH	1.06, m	45.8, CH	1.09, m
7	44.9, CH	3.00, br s	48.5, CH	2.93, br s	46.2, CH	2.96, br s
8	19.4, CH_3_	0.93, s	20.3, CH_3_	0.92, s	19.6, CH_3_	0.98, s
9	31.7, CH	1.03, overlap	32.8, CH	1.18, m	32.7, CH	1.01, overlap
10	20.8, CH_3_	0.75, d (6.0)	21.2, CH_3_	0.81, d (6.6)	21.1, CH_3_	0.78, d (6.2)
11	21.8, CH_3_	1.04, overlap	22.1, CH_3_	1.00, overlap	22.1, CH_3_	1.03, d (6.2)
12	11.0, CH_3_	2.02, s	12.6, CH_3_	2.01, s	10.9, CH_3_	2.07, s
13	63.5, CH	2.06, d (9.5)	65.5, CH	2.06, d (9.3)	64.7, CH	2.14, d (9.2)
14	134.5, CH	5.68, ddd (15.4, 9.5, 1.4)	135.2, CH	5.79, ddd (15.4, 9.3, 1.4)	134.8, CH	5.69, ddd (15.5, 9.3, 1.4)
15	188.2, CH	10.04, s	169.9, C		190.1, CH	10.03, s
16	127.5, CH	5.51, dd (15.3, 5.8)	129.8, CH	5.62, dd (15.3, 6.1)	129.8, CH	5.60, dd (15.4, 5.9)
17	71.5, CH	4.58, dd (5.8, 1.4)	73.2, CH	4.52, d (6.1, 1.4)	72.6, CH	4.52, dd (5.9, 1.4)
18	174.2, C		177.0, C		176.3, C	
19	53.0, CH_3_	3.77, s	49.8, CH_3_	3.35, s		

a Measured in CDCl_3_.

b Measured in CD_3_OD.

Bipolarisorokin K (**2**): colorless oil; ${\mathrm{[\alpha ]}}_{D}^{20}$ – 58.9 (*c* 0.05, MeOH); UV (MeOH) *λ*_max_ (log *ε*) 245 (3.82); ^1^H NMR (600 MHz, CD_3_OD) and ^13^C NMR (150 MHz, CD_3_OD) data, see Table 1; positive ion HR-ESI-MS *m/z* 359.18268, [M + Na]^+^, (calculated for C_19_H_28_NaO_5_^+^, 359.18290).

Bipolarisorokin L (**3**): colorless oil; ${\mathrm{[\alpha ]}}_{D}^{17}$ – 83.3 (*c* 0.05, MeOH); UV (MeOH) *λ*_max_ (log *ε*) 265 (3.78); ^1^H NMR (600 MHz, CD_3_OD) and ^13^C NMR (150 MHz, CD_3_OD) data, see Table 1; positive ion HR-ESI-MS *m/z* 307.19046, [M + H]^+^, (calculated for C_18_H_27_O_4_^+^, 307.19039).

Bipolarisorokin M (**4**): colorless oil; ${\mathrm{[\alpha ]}}_{D}^{17}$ – 48.9 (*c* 0.05, MeOH); UV (MeOH) *λ*_max_ (log *ε*) 265 (4.15); ^1^H NMR (600 MHz, CDCl_3_) and ^13^C NMR (150 MHz, CDCl_3_) data, see Table 2; positive ion HR-ESI-MS *m/z* 525.35706, [M + H]^+^, (calculated for C_33_H_49_O_5_^+^, 525.35745).

**TABLE 2:** ^1^H and ^13^C NMR data for **4** and **5** in CDCl_3_.

No.	4		5	
	*δ*_C_, type	*δ*_H_ (*J* in Hz)	*δ*_C_, type	*δ*_H_ (*J* in Hz)
1	137.7, C		137.7, C	
2	165.9, C		165.9, C	
3	52.3, C		52.3, C	
4a	33.8, CH_2_	1.36, td (12.8, 5.8)	33.8, CH_2_	1.37, d (5.6)
4b		1.45, dd (13.3, 6.3)		1.45, dd (13.3, 6.3)
5a	25.4, CH_2_	0.86, m	25.4, CH_2_	0.88, m
5b		1.74, overlap		1.75, overlap
6	44.5, CH	1.05, overlap	44.5, CH	1.04, overlap
7	45.0, CH	2.98, br s	45.0, CH	2.99, br s
8	19.6, CH_3_	0.95, s	19.6, CH_3_	0.95, s
9	31.7, CH	1.03, overlap	31.7, CH	1.02, overlap
10	20.9, CH_3_	0.76, overlap	20.9, CH_3_	0.75, d (5.8)
11	21.9, CH_3_	1.05, overlap	21.9, CH_3_	1.04, overlap
12	10.8, CH_3_	2.02, s	11.0, CH_3_	2.02, s
13	63.6, CH	2.08, d (9.5)	63.6, CH	2.08, d (9.5)
14	134.3, CH	5.70, dd (15.3, 9.5)	134.4, CH	5.70, dd (15.2, 9.6)
15	188.2, CH	10.05, s	188.2, CH	10.05, s
16	127.2, CH	5.52, dd (15.3, 5.5)	127.4, CH	5.53, dd (15.3, 5.6)
17	71.2, CH	4.56, d (5.5)	71.3, CH	4.58, d (5.4)
18	173.8, C		173.9, C	
1′	137.3, C		126.0, C	
2′	165.1, C		161.7, C	
3′	51.0, C		50.8, C	
4'	34.0, CH_2_	1.42, overlap	33.8, CH_2_	1.34, dd (13.0, 5.6)
5′a	25.2, CH_2_	0.89, overlap	25.0, CH_2_	0.99, overlap
5′b		1.77, overlap		1.76, overlap
6′	44.9, CH	1.01, overlap	45.2, CH	0.99, overlap
7′	41.7, CH	3.09, br s	43.6, CH	3.06, br s
8′	18.7, CH_3_	1.04, s	19.2, CH_3_	0.99, s
9′	31.7, CH	1.03, m	31.6, CH	1.22, m
10′	20.8, CH_3_	0.76, overlap	21.0, CH_3_	0.8, d (6.5)
11′	21.8, CH_3_	1.05, overlap	21.8, CH_3_	1.04, overlap
12′	11.0, CH_3_	2.02, s	12.8, CH_3_	2.03, s
13′	57.7, CH	1.81, dd (8.7, 5.9)	58.2, CH	1.76, overlap
14′a	66.0, CH_2_	3.80, dd (10.9, 8.9)	66.3, CH_2_	3.91, m
14′b		4.25, dd (11.0, 5.8)		4.27, dd (10.8, 5.6)
15′	188.1, CH	10.05, s	171.3, C	

Bipolarisorokin N (**5**): colorless oil; ${\mathrm{[\alpha ]}}_{D}^{17}$ – 45.6 (*c* 0.05, MeOH); UV (MeOH) *λ*_max_ (log *ε*) 255 (4.07); ^1^H NMR (600 MHz, CDCl_3_) and ^13^C NMR (150 MHz, CDCl_3_) data, see Table 2; positive ion HR-ESI-MS *m/z* 541.35254, [M + H]^+^, (calculated for C_33_H_49_O_6_^+^, 541.35237).

Bipolariterpene A (**6**): colorless oil; ${\mathrm{[\alpha ]}}_{D}^{20}$ ‒ 25.3 (*c* 0.05, MeOH); UV (MeOH) *λ*_max_ (log *ε*) 260 (3.96); ^1^H NMR (600 MHz, CD_3_OD) and ^13^C NMR (150 MHz, CD_3_OD) data, see Table 3; positive ion HR-ESI-MS *m/z* 483.27176, [M + Na]^+^, (calculated for C_27_H_40_NaO_6_^+^, 483.27171).

**TABLE 3:** ^1^H and ^13^C NMR data for **6**–**8** (*δ* in ppm).

no.	6^a^		7^a^		8^b^	
	*δ*_C_, type	*δ*_H_ (*J* in Hz)	*δ*_C_, type	*δ*_H_ (*J* in Hz)	*δ*_C_, type	*δ*_H_ (*J* in Hz)
1	50.2, C		49.8, C		49.0, C	
2a	40.5, CH_2_	1.69, overlap	39.9, CH_2_	1.94, m	39.3, CH_2_	2.23, dd (14.8, 8.0)
2b		2.35, m		2.07, m		1.78, m
3	123.3, CH	5.33, overlap	120.9, CH	5.26, m	119.6, CH	5.12, m
4	138.6, C		138.4, C		139.2, C	
5a	41.3, CH_2_	2.31, m	42.9, CH_2_	2.78, m	35.3, CH_2_	2.29, dd (14.5, 7.2)
5b		2.06, m				2.11, m
6a	24.3, CH_2_	2.44, overlap	126.1, CH	5.75, m	30.1, CH_2_	1.81, m
6b		2.22, m				1.60, m
7	128.7, CH	5.35, overlap	138.0, CH	5.48, d (15.5)	75.7, CH	3.38, dd (8.1, 3.7)
8	137.6, C		74.3, C		74.8, C	
9a	31.4, CH_2_	2.42, overlap; 1.77, m	39.0, CH_2_	1.74, m	35.3, CH_2_	1.65, overlap
9b				1.64, m		1.43, m
10a	31.0, CH_2_	1.79, m	30.6, CH_2_	1.62, m	29.5, CH_2_	1.76, overlap
10b		1.61, overlap		1.52, m		1.51, m
11	77.1, CH	4.01, dd (9.3, 4.3)	80.5, CH	3.88, m	79.1, CH	4.09, m
12	137.6, C		139.1, C		138.1, C	
13	130.0, CH	5.39, d (5.4)	127.4, CH	5.20, d (8.1)	126.2, CH	5.43, d (5.3)
14a	30.2, CH_2_	2.44, overlap	30.0, CH_2_	2.34, d (15.7)	29.6, CH_2_	2.36, d (16.1)
14b		1.95, dt (17.9, 9.4)		1.87, m		1.93, m
15	50.7, CH	2.72, d (10.6)	50.9, CH	2.50, d (9.5)	49.8, CH	2.54, d (8.7)
16	149.9, C		152.2, C		147.4, C	
17	149.5, C		148.8, C		146.6, C	
18	209.0, C		210.1, C		207.8, C	
19	16.8, CH_3_	0.96, s	16.5, CH_3_	0.99, s	16.0, CH_3_	1.00, s
20	15.5, CH_3_	1.66, s	18.0, CH_3_	1.67, s	17.8, CH_3_	1.63, s
21a	59.5, CH_2_	4.20, d (12.0)	30.4, CH_3_	1.26, s	24.6, CH_3_	1.24, s
21b		4.08, d (12.0)				
22	10.5, CH_3_	1.57, s	11.4, CH_3_	1.56, s	12.3, CH_3_	1.62, s
23	35.2, CH	2.81, q (7.1)	38.8, CH	2.59, q (6.9)	34.1, CH	2.77, q (7.2)
24a	67.6, CH_2_	4.31, m	65.8, CH_2_	3.82, m	66.5, CH_2_	4.27, dd (10.5, 7.8)
24b		4.26, m		3.68, dd (10.4, 6.5)		4.22, dd (10.6, 7.0)
25	14.7, CH_3_	1.30, d (7.0)	14.6, CH_3_	1.24, d (7.1)	14.7, CH_3_	1.31, d (7.1)
26	172.7, C				171.1, C	
27	20.8, CH_3_	2.00, s			21.0, CH_3_	2.02, s

a Measured in CD_3_OD.

b Measured in CDCl_3_.

Bipolariterpene B (**7**): colorless oil; ${\mathrm{[\alpha ]}}_{D}^{20}$ ‒ 28.9 (*c* 0.04, MeOH); UV (MeOH) *λ*_max_ (log *ε*) 265 (3.92); ^1^H NMR (600 MHz, CD_3_OD) and ^13^C NMR (150 MHz, CD_3_OD) data, see Table 3; positive ion HR-ESI-MS *m/z* 441.26044, [M + Na]^+^, (calculated for C_25_H_38_NaO_5_^+^, 441.26115).

Bipolariterpene C (**8**): colorless oil; ${\mathrm{[\alpha ]}}_{D}^{20}$ + 26.3 (*c* 0.035, MeOH); UV (MeOH) *λ*_max_ (log *ε*) 205 (4.05); ^1^H NMR (600 MHz, CDCl_3_) and ^13^C NMR (150 MHz, CDCl_3_) data, see Table 3; positive ion HR-ESI-MS *m/z* 461.28955, [M + H]^+^, (calculated for C_27_H_41_O_6_^+^, 461.28977).

### 2.4 Preparation of (*S*)-MTPA and (*R*)-MTPA esters of 1

The samples of **1** (1.5 mg each) were dissolved in pyridine (500 μl), and added with DMAP (2 mg) and (*R*)- or (*S*)-MTPA-Cl (10 μl) to the solution. The reaction was stirred at room temperature for 12 h. The productions were individually purified by semipreparative HPLC and eluted with MeCN-H_2_O (78:22, *v*/*v*, 4.0 ml/min) to obtain the (*S*)-MTPA ester **1a** (1.0 mg, *t*_*R*_ = 14.0 min) and (*R*)-MTPA ester **1b** (0.8 mg, *t*_*R*_ = 14.0 min), respectively.

(*S*)-MTPA ester (**1a**). ^1^H NMR (600 MHz, CDCl_3_): 1.44 (1H, d, *J* = 6.9 Hz, H-4a), 1.35 (1H, m, H-4b), 1.75 (1H, dd, *J* = 12.0, 6.7 Hz, H-5a), 0.88 (1H, m, H-5b), 1.06 (1H, overlap, H-6), 3.00 (1H, br s, H-7), 0.92 (3H, s, H-8), 1.03 (1H, overlap, H-9), 0.76 (3H, d, *J* = 5.8 Hz, H-10), 1.05 (3H, overlap, H-11), 2.01 (3H, s, H-12), 2.09 (1H, d, *J* = 9.4 Hz, H-13), 5.79 (1H, dd, *J* = 15.3, 9.4 Hz, H-14), 10.05 (1H, s, H-15), 5.62 (1H, dd, *J* = 15.3, 7.4 Hz, H-16), 5.55 (1H, d, *J* = 7.5 Hz, H-17), 3.74 (3H, s, H-19); positive ion HR-ESI-MS *m/z* 537.24640, [M + H]^+^, (calculated for C_29_H_36_F_3_O_6_^+^, 537.24585).

(*R*)-MTPA ester (**1b**). ^1^H NMR (600 MHz, CDCl_3_): 1.43 (1H, m, H-4a), 1.34 (1H, m, H-4b), 1.74 (1H, m, H-5a), 0.83 (1H, m, H-5b), 1.01 (1H, overlap, H-6), 2.93 (1H, br s, H-7), 0.86 (3H, s, H-8), 1.00 (1H, overlap, H-9), 0.75 (3H, d, *J* = 5.3 Hz, H-10), 1.02 (3H, overlap, H-11), 1.98 (3H, s, H-12), 2.04 (1H, d, *J* = 9.4 Hz, H-13), 5.67 (1H, m, H-14), 10.02 (1H, s, H-15), 5.56 (1H, overlap, H-16), 5.57 (1H, overlap, H-17), 3.77 (3H, s, H-19); positive ion HR-ESI-MS *m/z* 559.22748, [M + Na]^+^, (calculated for C_29_H_35_F_3_NaO_6_^+^, 559.22779).

### 2.5 NMR calculations

The NMR calculations were carried out using the Gaussian 16 software package (Frisch et al., 2010). Systematic conformational analyses were performed *via* SYBYL-X 2.1 using the MMFF94 molecular mechanics force field calculation with 10 kcal/mol of cutoff energy (Hehre, 2003; Shao et al., 2006). The optimization and frequency of conformers were calculated on the B3LYP/6-31G(d) level in the Gaussian 16 program package. All the optimized conformers in an energy window of 5 kcal/mol (with no imaginary frequency) *w*ere subjected to gauge-independent atomic orbital (GIAO) calculations of their ^13^C NMR chemical shifts, using density functional theory (DFT) at the mPW1PW91/6-311 + G (d,p) level with the PCM model. The calculated NMR data of these conformers were averaged according to the Boltzmann distribution theory and their relative Gibbs free energy. The ^13^C NMR chemical shifts for TMS were also calculated by the same procedures and used as the reference. After the calculation, the experimental and calculated data were evaluated by the improved probability DP4^+^ method (Grimblat et al., 2015).

### 2.6 Antibacterial activity assay

All compounds were evaluated for their antibacterial activity against *Pseudomonas syringae* pv. *Actinidae.* The antibacterial assay was conducted by the previously described method (Yu et al., 2022). The sample to be tested was added into a 96-well culture plate, and the final compound concentration range from 4 to 256 *μ*g/ml. Bacteria liquid was added to each well until the final concentration is 5 × 10^5^ CFU/ml. It was then incubated at 27°C for 24 h, and the minimum inhibitory concentration (MIC, with an inhibition rate of ≥90%) was determined by the microplate reader at OD_600_ nm. The medium blank control was used in the experiment. Streptomycin was used as the positive control.

## 3 Results and Discussion

### 3.1 Structure characterizations

**FIGURE 2:**
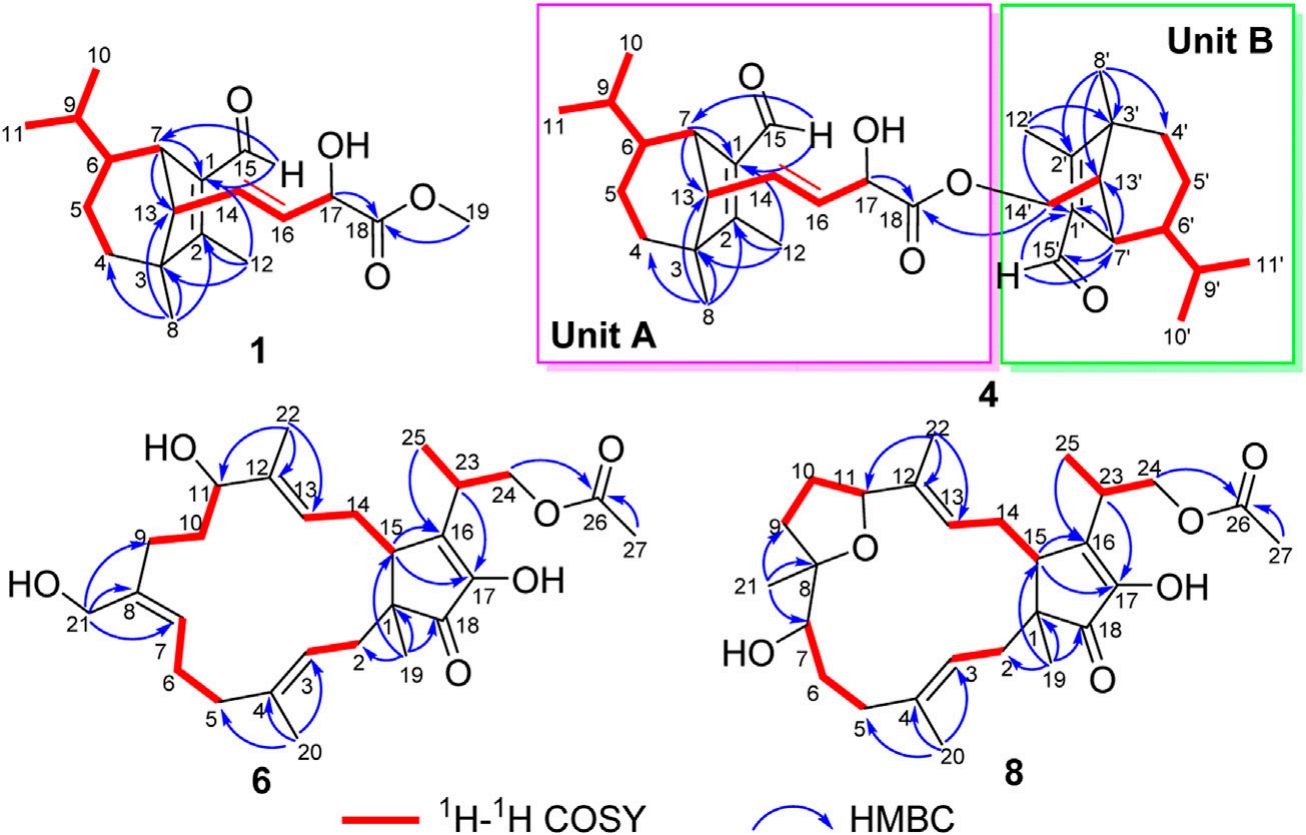
Key ^1^H−^1^H COSY and HMBC correlations for **1**, **4**, **6,** and **8**.

**FIGURE 3:**
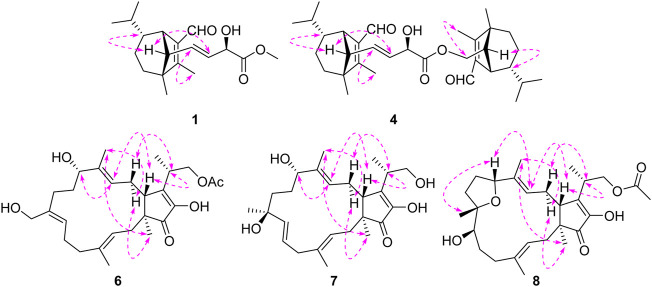
Key ROESY correlations for **1**, **4**, **6**, **7**, and **8**.

**FIGURE 4:**
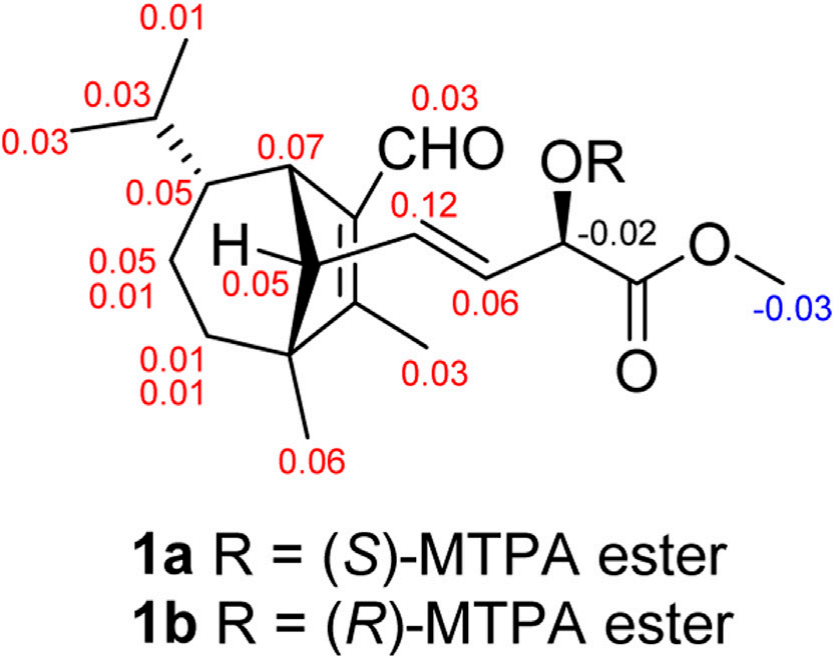
Δ*δ* = *δ*_S_ − *δ*_R_ values in ppm obtained from the MTPA esters of **1**.

Bipolarisorokin J **1**) was isolated as a colorless oil. The molecular formula was determined as C_19_H_28_O_4_ with six degrees of unsaturation based on the HRESIMS data (measured at *m/z* 343.18790 [M + Na]^+^, calcd for C_19_H_28_NaO_4_^+^ 343.18798). The ^13^C NMR data of **1** displayed 19 carbon signals, which were assigned as five methyls, two methylenes, eight methines, and four quaternary carbons in association with the HSQC data (Table 1). The ^1^H NMR data of **1** showed five methyl signals at *δ*_H_ 0.93 (3H, s, H-8), 0.75 (3H, d, *J* = 6.0 Hz, H-10), 1.04 (3H, overlap, H-11), 2.02 (3H, s, H-12), and 3.77 (3H, s, H-19), two olefinic protons at *δ*_H_ 5.68 (1H, ddd, *J* = 15.4, 9.5, 1.4 Hz, H-14) and 5.51 (1H, dd, *J* = 15.3, 5.8 Hz, H-16), and an aldehyde proton at *δ*_H_ 10.04 (H, s, H-15) (Table 1). The characteristic signals of 1D NMR, together with the data of analogues from the same origin, suggested that **1** was most likely a seco-sativene type sesquiterpenoid derivative. According to ^1^H−^1^H COSY spectrum, two structural fragments were deduced as shown with bold lines in Figure 2. Based on this, the HMBC correlations from *δ*_H_ 2.02 (3H, s, Me-12) to *δ*_C_ 165.9 (s, C-2), 52.4 (s, C-3) and *δ*_C_ 137.8 (s, C-1), from *δ*_H_ 0.93 (3H, s, Me-8) to C-2, C-3, 33.8 (t, C-4) and 63.5 (d, C-13), from *δ*_H_ 3.00 (1H, br s, H-7) to C-1, C-13, and *δ*_C_ 134.5 (d, C-14) established a seco-sativene type sesquiterpene backbone. In addition, one aldehyde group was connected to C-1, which was deduced from HMBC correlations from *δ*_H_ 10.04 (H, s, H-15) to *δ*_C_ 44.9 (d, C-7) and C-1. Furthermore, the HMBC correlations from *δ*_H_ 3.77 (3H, s, Me-19) and 4.58 (1H, dd, *J* = 5.8, 1.4 Hz, H-17) to *δ*_C_ 174.2 (s, C-18) suggested that the connections among C-17, C-18 and C-19. The planar structure of **1** was thus deduced as shown in Figure 2, resembling bipolarisorokin G (**10**) (Yu et al., 2022). The ROESY correlations (Figure 3) of H-13/H-8, H-13/H-6 and H-12/H-14 revealed that H-3, H-6, H-7 and H-8, were co-facial and assigned to be *β*-oriented. Correlations between H-13 and H-16, as well as large coupling constants (*J* = 15.4 Hz), confirmed the double bonds (C-14 and C-16) to be *E*-geometry. However, the geometry of H-17 cannot be determined by using the NOESY correlation. Regarding the same origin of **1** and **10**, the absolute configuration of **1** thus was suggested to be the same as that of **10**, except for C-17. However, the stereo-chemistry at C-17 was determined using a modifie Mosher’s method (Hoye et al., 2007). The observed differences of chemical shifts (Δ*δ* = *δ*_S_ − *δ*_R_) (Figure 4) indicated that the C-17 absolute configuration is *R*. Consequently, the absolute configuration of **1** was assigned as 3*R*, 6*R*, 7*S*, 13*S*, 17*R*.

**FIGURE 5:**
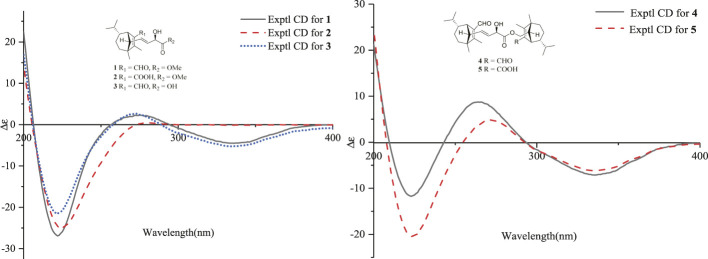
ECD spectra of compounds **1**–**5** in methanol.

Bipolarisorokin K (**2**), a colorless oil, was assigned the molecular formula of C_19_H_28_O_5_ with six degrees of unsaturation based on HRESIMS data (measured at *m/z* 359.18268 [M + Na]^+^, calcd for C_19_H_28_NaO_4_^+^ 359.18290). The ^1^H and ^13^C NMR data of **2** (Table 1) are closely similar to those of **1**. The significant difference was the presence of a carboxyl group at C-15 (*δ*_C_ 169.9, s) in **2**, instead of the aldehyde group in **1**. This deduction was identified by the HMBC corrections from *δ*_H_ 2.93 (1H, br s, H-7) to *δ*_C_ 128.2 (s, C-1), *δ*_C_ 160.0 (s, C-2) and C-15, together with its HRESIMS data. Moreover, the absolute configuration of **2** was suggested to be the same with that of **1** based on the nearly identical NMR data, the biosynthetic pathway, and the consistent experimental ECD data of these two compounds (Figure 5).

Bipolarisorokin L **3**) was obtained as a colorless oil. Its molecular formula was determined to be C_18_H_26_O_4_ based on HRESIMS data (measured at *m/z* 307.19046 [M + H]^+^, calcd for C_18_H_27_O_4_^+^ 307.19039). Comparing its 1D and 2D NMR data with those of **1** indicated that they shared almost the same chemical construction. However, the major difference was that the methyl ester group in **1** was replaced by a carboxyl group at C-18 in **3**. The loss of a methoxy signal in the ^13^C NMR spectrum, the HMBC correlation from *δ*_H_ 4.52 (1H, dd, *J* = 5.9 Hz, 1.4 Hz, H-17) to *δ*_C_ 176.3 (s, C-18), and the mass data analysis confirmed the above deduction. The relative configurations of in **3** should be in agreement with the configuration of **1** based on the nearly identical NMR data. Finally, the experimental ECD curve of **3** matched well with that of **1** (Figure 5), suggesting that the absolute configuration of **3** was identical to that of **1**.

Bipolarisorokin M **4**) was obtained as a colorless oil. Its molecular formula of C_33_H_48_O_5_, together with ten degrees of unsaturation, were established by its HRESIMS data (measured at *m/z* 525.35706 [M + H]^+^, calcd for C_33_H_49_O_5_^+^ 525.35745). The ^1^H NMR data of **4** displayed signals for two olefinic protons, eight methyl groups, and two protons of aldehyde group (Table 2). The ^13^C NMR and DEPT data of **4** exhibited 33 carbon resonances, including eight methyls, five methenes (one oxygenated), twelve methines (one oxygenated, two olefinic and two aldehyde carbons), seven nonprotonated carbons (four olefinic and one ester carbonyl) (Table 2). After literature investigations, the aforementioned NMR data indicated that compound **4** should comprise of two different seco-sativene sesquiterpenoid units. Interpretation of the ^1^H−^1^H COSY spectrum of **4** revealed the presence of four discrete proton−proton spin systems as shown with bold lines in Figure 2. Further analysis of its HMBC spectra demonstrated the existence of two building blocks of units A and B, which were highly similar to **3** and **11**, respectively. The above deduction was confirmed by the HMBC correlations as shown in Figure 2, together with comparison of the ^1^H and ^13^C NMR spectroscopic data. Meanwhile, the key HMBC correlation from *δ*_H_ 3.80 (1H, dd, *J* = 10.9 Hz, 8.9 Hz, H-14′a) and 4.25 (1H, dd, *J* = 11.0 Hz, 5.8 Hz, H-14′b) to *δ*_C_ 173.8 (s, C-18), along with analysis of the HRESIMS data, suggested the connection by an ester bond between units A and B. Therefore, considering similar NMR data and coupling constants, as well as their concurrent biogenetic relationship, the absolute configurations of **4** should agree with those of **3** and **11**, respectively. Finally, the structure of **4** was established as depicted in Figure 1.

Bipolarisorokin N **5**) was also isolated as a colorless oil. Its molecular formula was established as C_33_H_48_O_6_ based on the HRESIMS ion peak at *m*/*z* 541.35254 [M + H]^+^ (calcd for C_33_H_49_O_6_^+^, 541.35237), corresponding to ten degrees of unsaturation. The 1D NMR data of **5** closely resembled those of **4** (Table 2), except for the obviously shifted signal of C-15′ (−16.8 ppm) and the absence of aldehyde hydrogen proton signal at H-15′. The HMBC correlations from *δ*_H_ 3.06 (1H, br s, H-7′) to *δ*_C_ 126.0 (s, C-1′) and 171.3 (s, C-15′), as well as the HRESIMS data analysis, led to the location of a carboxyl group (C-15′) at C-1′. Furthermore, the similar ROESY data and experimental ECD curves of **4**–**5** (Figure 5) suggested that they shared the same absolute configuration. Therefore, the structure of **5** was finally established as shown in Figure 1.

Bipolariterpene A **6**) was assigned a molecular formula of C_27_H_40_O_6_ with eight degrees of unsaturation based on its HRESIMS data (measured at *m/z* 483.27176 [M + Na]^+^, calcd for C_27_H_40_NaO_6_^+^ 483.27171). The ^1^H and ^13^C NMR data (Table 3) showed 27 carbon resonances comprising five methyls (*δ*_C_ 16.8, 15.5, 10.5, 14.7, and 20.8); eight methylenes including six aliphatic ones (*δ*_C_ 40.5, 41.3, 24.3, 31.4, 31.0, and 30.2) and two oxygenated ones at *δ*_C_ 59.5 and 67.6; six methines including two aliphatic ones at *δ*_C_ 50.7 and 35.2, three olefinic ones (*δ*_C_ 123.3, *δ*_C_ 128.7 and *δ*_C_130.0), and a oxygenated one at *δ*_C_ 77.1; eight non-protonated carbons with a aliphatic one at *δ*_C_ 50.2, five olefinic ones (*δ*_C_ 138.6, *δ*_C_ 137.6 × 2, *δ*_C_ 149.9, *δ*_C_ 149.5), a carbonyl one at *δ*_C_ 209.9, and a ester carbonyl one at *δ*_C_ 172.7. The general features of its NMR data closely resembled those of the co-isolated known bicyclic sesterterpene fusaproliferin **13**) (Nihashi et al., 2002; Gao et al., 2020). The major difference was that an additional hydroxy group was substituted at C-21 in **6**, which could be fully established through the HMBC corrections from *δ*_H_ 4.20 (1H, d, *J* = 12.0 Hz, H-21a) and 4.08 (1H, d, *J* = 12.0 Hz, H-21b) to *δ*_C_ 128.7 (d, C-7), 137.6 (s, C-8) and 31.4 (t, C-9). The ROESY correlations between Me-20/H-2b, H-3/H-5b, H-21a/H-6a, H-7/H-9a, H-11/H-13, Me-22/H-14b indicated that the configurations of C-3/C-4, C-7/C-8, and C-12/C-13 double bonds were assigned as *E*, *Z*, and *E*, respectively. Furthermore, as indicated by its ROESY spectrum, the cross-peaks of H-11/H-13, H-13/H-15, H-15/H-23 suggested that H-11, H-15 and H-23 is *β*-oriented. Meanwhile, the key interaction of H-14b/Me-19, Me-22/Me-19 and H-14b/Me-22, along with the lack of H-15/Me-19, implied that Me-19 was *α*-oriented. Thus, compound **6** was determined to share the same stereochemistry with that of **13** for which the absolute configuration had been earlier confirmed by X-ray crystallographic analysis (Santini et al., 1996) and total enantioselective synthesis (Myers et al., 2002). By comparing the specific rotation of **6** ( ${\mathrm{[\alpha ]}}_{D}^{20}$ ‒25.3) with **13** ( ${\mathrm{[\alpha ]}}_{D}^{25}$ ‒35) (Santini et al., 1996), as well as the close biogenetic relationship, the absolute configuration of **6** was further identified to be the same as that of **13**, as depicted in Figure 1.

**FIGURE 6:**
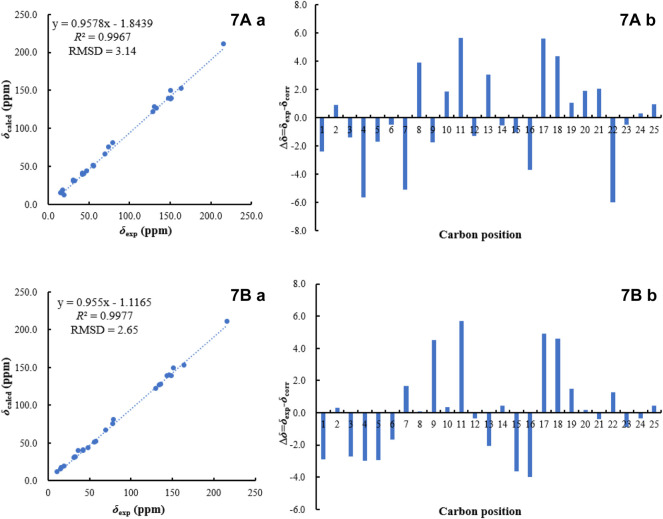
^13^C NMR calculation results of two possible isomers of **7 (A)**: Linear correlation plots of predicted versus experimental ^13^C NMR chemical shift. **(B)** Relative errors between the predicted *δ*_C_ of two potential structures and recorded *δ*_C._).

Bipolariterpene B **7**) was obtained as a colorless oil. The molecular formula of **7** was assigned as C_25_H_38_O_5_ based on its HRESIMS spectrum (measured at *m/z* 441.26044 [M + Na]^+^, calcd for C_25_H_38_NaO_5_^+^ 441.26115), which showed two fewer carbon atoms than fusaproliferin (**13**) (Nihashi et al., 2002). Additionally, the ^1^H and ^13^C NMR data of **7** were similar to those of **13** (Table 3). The significant difference between **7** and **13** was the absence of an acetyl group in **7**, which was confirmed by the HMBC correlations from *δ*_H_ 3.82 (1H, m, H-24a) and 3.68 (1H, dd, *J* = 10.4, 6.5 Hz, H-24b) to *δ*_C_ 14.6 (q, C-25), 38.8 (d, C-23) and 152.2 (s, C-16), together with its HRESIMS data. Moreover, the HMBC correlations from *δ*_H_ 1.26 (3H, s, H-21) to *δ*_C_ 138.0 (d, C-7), 74.3 (s, C-8) and 39.0 (t, C-9), as well as chemical shift of C-8, indicated that an additional hydroxy group was located at C-8. Furthermore, the HMBC correlations from *δ*_H_ 5.75 (1H, m, H-6) and 5.48 (1H, d, *J* = 15.5 Hz, H-7) to *δ*_C_ 42.9 (t, C-5) and C-8, along with the observed ^1^H−^1^H COSY cross-peak of *δ*_H_ 2.78 (2H, m, H-5)/H-6/H-7, confirmed that one double bond between C-7 and C-8 in **13** migrated to C-6 and C-7 in **7**. Based on the biogenetic and NOESY data consideration, the absolute configuration of **7** was proposed to be consistent with the known compound **13**, except for C-8. To determine its absolute configuration, the NMR calculations with DP4+ analysis for two possible isomers (1*S*, 8*R*, 11*S*, 15*R*, 23*S*)-**7**A and (1*, 8*, 11*S*, 15*R*, 23*S*)-**7**B were carried out using the GIAO method at the mPW1PW91/6-311+G (d,p) level with the PCM model. As a result, the calculated chemical shifts of **7**B matched well with the experimental ones (Figure 6), showing a better correlation coefficient (*R* ^2^ = 0.9977) and a low root-mean-square deviation value (RMSD = 2.65), together with a high DP4+ probability of 100% (all data) probability (Supplementary Tables S3 in the Supporting Information). Hence, compound **7** was identified as shown in Figure 1 and named as bipolariterpene B.

Bipolariterpene C **8**) was also isolated as a colorless oil. Its molecular formula was established as C_27_H_40_O_6_ based on the HRESIMS ion peak at *m*/*z* 461.28955 [M + H]^+^ (calcd for C_27_H_41_O_6_^+^, 461.28977), suggesting eight degrees of unsaturation. Analyses of NMR spectra (Table 3) indicated that the structure of **8** was explicitly similar to that of fusaprolifin A (Liu et al., 2013). However, the signals for two olefinic methines were replaced by signals of a methylene *δ*_C_ 30.1 (t, C-6) and an oxygenated methine *δ*_C_ 75.7 (d, C-7). These observations indicated the hydration of the double bond in fusaprolifin A, leading to the location of a hydroxy group at C-7 in **8**. It was supported by HMBC correlations from *δ*_H_ 3.38 (1H, dd, *J* = 8.1, 3.7 Hz, H-7) to *δ*_C_ 35.3 (t, C-5), 30.1 (t, C-6), 74.8 (s, C-8), 35.3 (t, C-9) and 24.6 (q, C-21), along with the ^1^H–^1^H COSY cross peaks of H-5/H-6/H-7. Furthermore, the chiral centers in **8**, except for C-7, were found to be identical to that of fusaprolifin A based on their highly similar coupling constant and ROESY data. In order to confirm the assigned chemical architecture of **8** and its the stereochemistry, the ^13^C NMR calculations and DP4+ analysis of (1*S*, 7*R*, 8*S*, 11*R*, 15*R*, 23*S*)-**8**A and (1*, 7*, 8*S*, 11*R*, 15*R*, 23*S*)-**8**B were carried out at the mPW1PW91/6-311+G (d,p) level. The results showed that **8**A was the most likely structure based on a better correlation coefficient (*R* ^2^ = 0.9970) and a low root-mean-square deviation value (RMSD = 3.05), as well as a high DP4 + probability of 100% (all data) probability (Supplementary Figure S6 and Supplementary Table S6 in the Supporting Information). Finally, the absolute configuration of **8** was defined.

In addition, the structures of five known compounds were identified as bipolarisorokin H (**9**), bipolarisorokin G (**10**), helminthosporol (**11**), helminthosporic acid (**12**) and fusaproliferin (**13**), by comparing the spectral data with those reported in the literature (Osterhage et al., 2002; Abdel-Lateff et al., 2013; Liu et al., 2013; Yu et al., 2022). In this study, compounds **1** and **2** were isolated as methyl esters, which could be derived from the separation process since methanol was used as the solvent. To verify whether these compounds are of natural origin, we analyzed the ethanol extract of the fermentation broth of the fungus by HPLC (see Supporting Information). As a results, all compounds could be confirmed their natural attributes.

**SCHEME 1:**
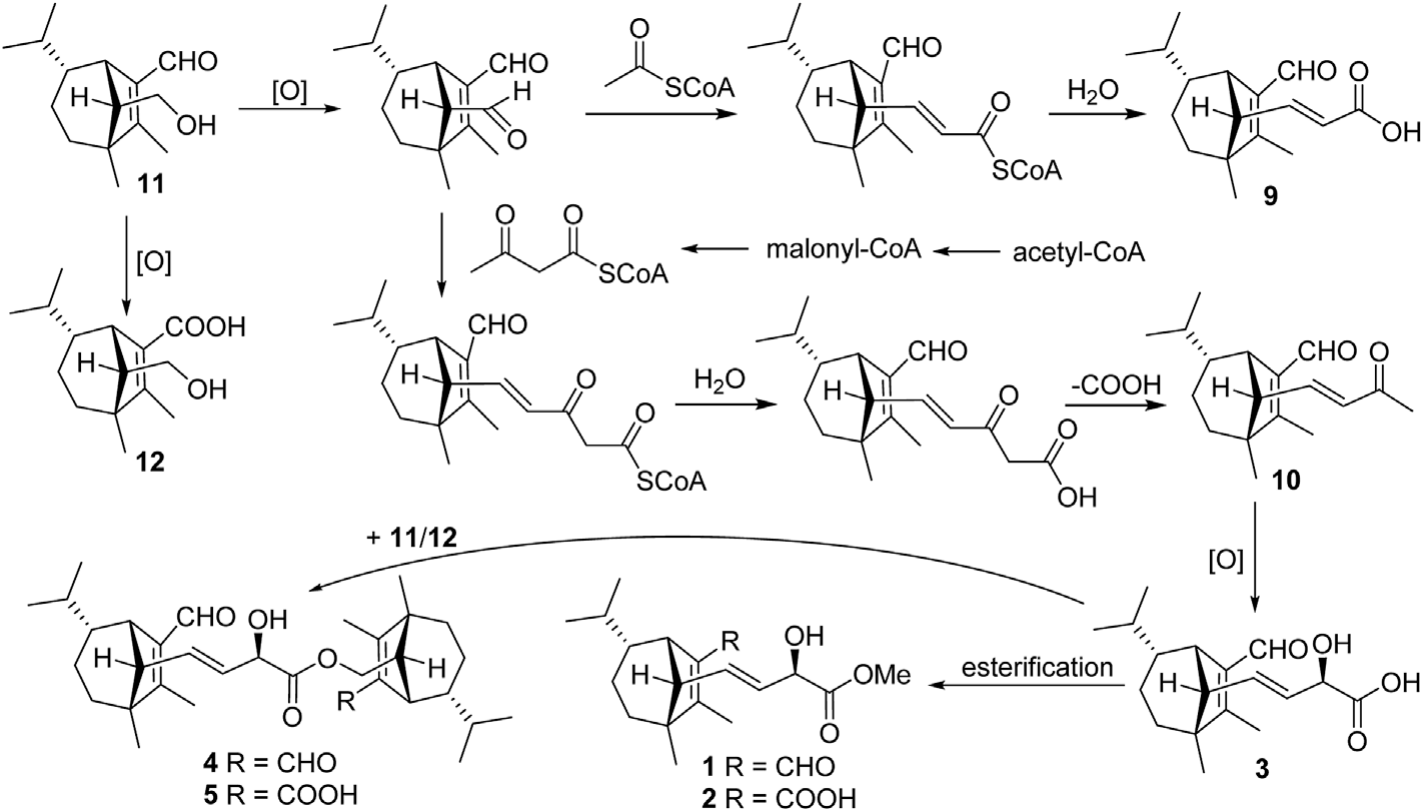
Plausible biogenetic pathway for seco-sativene sesquiterpenoids of *Bipolaris* sp.

Structurally, compound **9** possessed two additional skeletal carbons, while compounds **1**–**5** and **10** possessed three additional skeletal carbons, which might be derived from acetyl-CoA or acetoacetyl-CoA. Compounds **11** and **12** were isolated as major components, which were most probably employed as the original precursor to assemble the above compounds. The hydroxyl group at C-14 in **11** was oxidized to produce an aldehyde product, which then underwent aldol condensation with the acetyl-CoA to give **9**. Similarly, the aldehyde product combined an acetoacetyl-CoA to give compounds **1**–**3** and **10**. Finally, additional esterification happened between **3** and **11** or **12** led to the formation of **4** or **5**, respectively Scheme 1.

### 3.2 Anti-Psa activity

All compounds (**1**–**13**) were evaluated for their anti-Psa activity by using the method as described previously (Yu et al., 2022). Streptomycin was used as the positive control. As a result, compounds **4** and **5** showed certain inhibitory activity, with MICs of 32 and 64 *μ*g/ml, respectively. Additionally, compounds **1–3**, and **10** showed weak activity, with MICs of 128 *μ*g/ml (Table 4). The results demonstrated that the additional skeletal carbons of seco-sativene sesquiterpenoids may be vital for Psa inhibitory activity.

**TABLE 4:** Inhibitory effects of the isolates against Psa (MIC, *μ*g/mL).

Compd	Psa
**1**	128
**2**	128
**3**	128
**4**	32
**5**	64
**6**	NA ^b^
**7**	NA
**8**	256
**9**	256
**10**	128
**11**	256
**12**	NA
**13**	NA
Streptomycin^a^	8

a Positive control;

b NA = no activity at 256 *μ*g/ml.

## 4 Conclusion

In conclusion, eight new terpenoids (**1**–**8**), along with five known analogues (**9**–**13**) was identified from the culture medium of an endophyte *Bipolaris* sp, a fungus isolated from fresh and healthy stems of kiwifruit plants. Compounds **1**–**3**, together with the known compound **10**, represented novel structures of seco-sativene sesquiterpenoids possessing three additional skeletal carbons, which were only found in this fungus. In addition, compounds **4** and **5** were rare seco-sativene/seco-sativene adducts. In anti-Psa activity assay, compounds **4** and **5** displayed certain inhibitory activity against Psa. This study, together with our previous work (Yu et al., 2022), further supported that it is an effective approach to search for anti-Psa agents from endophytic fungi of kiwi plant itself. The endophyte *Bipolaris* sp. Could be a potential antibacterial strain, while its sativene sesquiterpene products could be potential anti-Psa agents.

## Data Availability

The original contributions presented in the study are included in the article/Supplementary Material, further inquiries can be directed to the corresponding authors.
