# Supplementary material for: Terpenoids from Kiwi endophytic fungus Bipolaris sp. and their antibacterial activity against Pseudomonas syringae pv. actinidiae
Source: Front Chem. 2022 Sep 1;10:990734. doi: 10.3389/fchem.2022.990734 (PMC9475172; doi:10.3389/fchem.2022.990734)
Supplement: Supplementary file 1 [file DataSheet1.PDF]

Supporting information for

Terpenoids from Kiwi Endophytic Fungus *Bipolaris* sp. and Their Antibacterial Activity *Pseudomonas syringae* pv. *actinidiae*

Jun-Jie Yu[1†], Wen-Ke Wei[1†], Yu Zhang[2], Russell J Cox[3], Juan He[1*], Ji-Kai Liu[1*], Tao Feng[1*]

[1]*School of Pharmaceutical Sciences, South-Central Minzu University, Wuhan, China*
[2]*State Key Laboratory of Phytochemistry and Plant Resources in West China, Kunming Institute of Botany, Chinese Academy of Sciences, Kunming, China*
[3]*Institute for Organic Chemistry and Biomolekulares Wirkstoffzentrum (BMWZ), Schneiderberg 38, 30167 Hannover, Germany*

*** Correspondence:**
Tao Feng
tfeng@mail.scuec.edu.cn

Ji-Kai Liu
liujikai@mail.scuec.edu.cn

Juan He
2015049@mail.scuec.edu.cn

[†]These authors contributed equally to this work

Content

Sections S1. Supplementary of NMR, HRESIMS and CD spectra

S1.1 NMR, HRESIMS and CD spectra of bipolarisorokin J

S1.2 NMR, HRESIMS and CD spectra of bipolarisorokin K

S1.3 NMR, HRESIMS and CD spectra of bipolarisorokin L

S1.4 NMR, HRESIMS and CD spectra of bipolarisorokin M

S1.5 NMR, HRESIMS and CD spectra of bipolarisorokin N

S1.6 NMR and HRESIMS spectra of bipolariterpene A

S1.7 NMR and HRESIMS spectra of bipolariterpene B

S1.8 NMR and HRESIMS spectra of bipolariterpene C

S1.9 ^{1}H NMR, ^{1}H–^{1}H COSY and HRESIMS spectra of (*S*)-MTPA ester (1a)

S1.10 ^{1}H NMR, ^{1}H–^{1}H COSY and HRESIMS spectra of (*R*)-MTPA ester (1b)

S1.11 HPLC analysis of ethanol extract of *Bipolaris* sp.

Sections S2. Computational details

S2.1 Computational details for bipolarisorokin B (NMR)

S2.2 Computational details for bipolariterpene C (NMR)

Sections S1. Supplementary of NMR, HRESIMS and CD spectra

S1.1 NMR, HRESIMS and CD spectra of bipolarisorokin J

^{1}H NMR spectrum

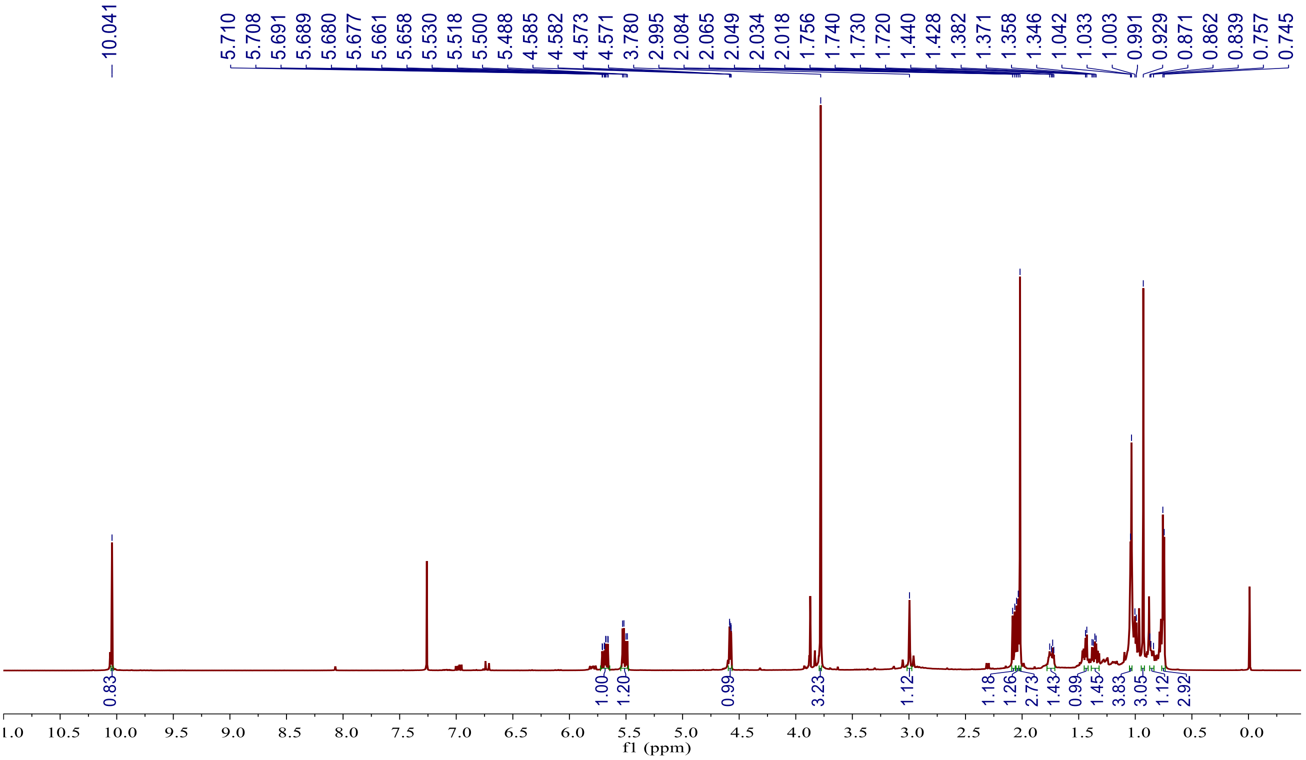

^{13}C NMR and DEPT spectra

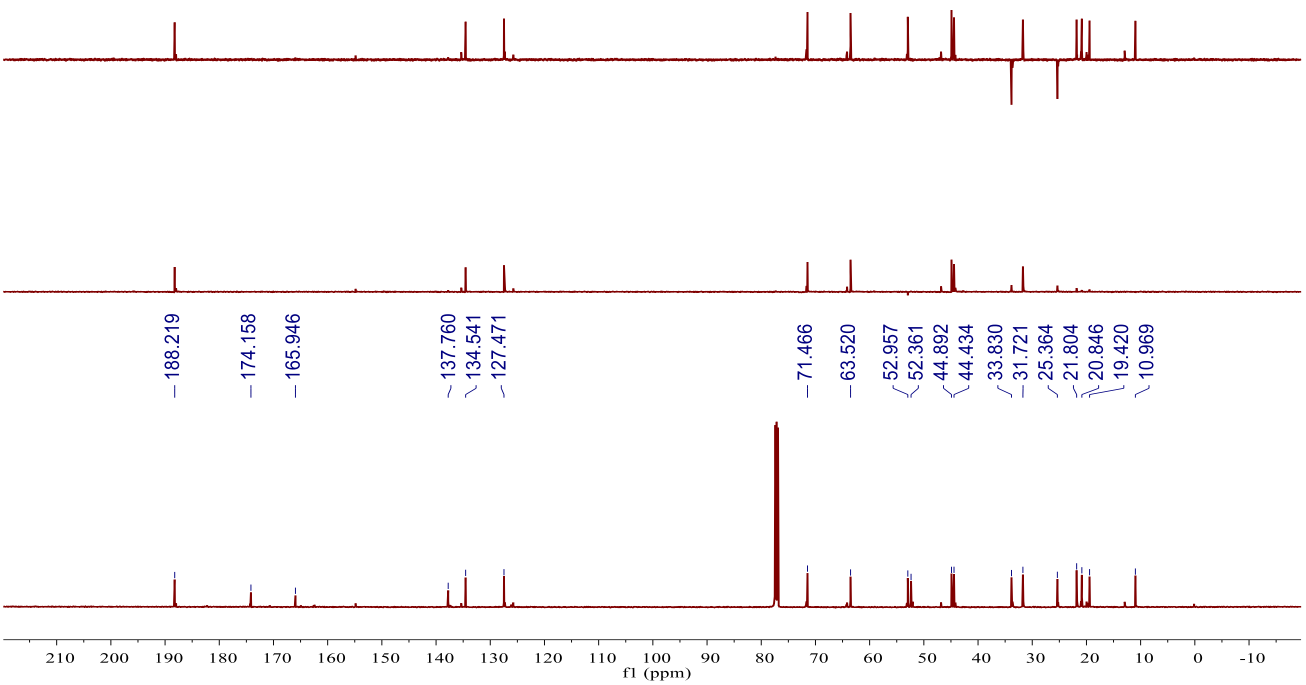

HSQC spectrum

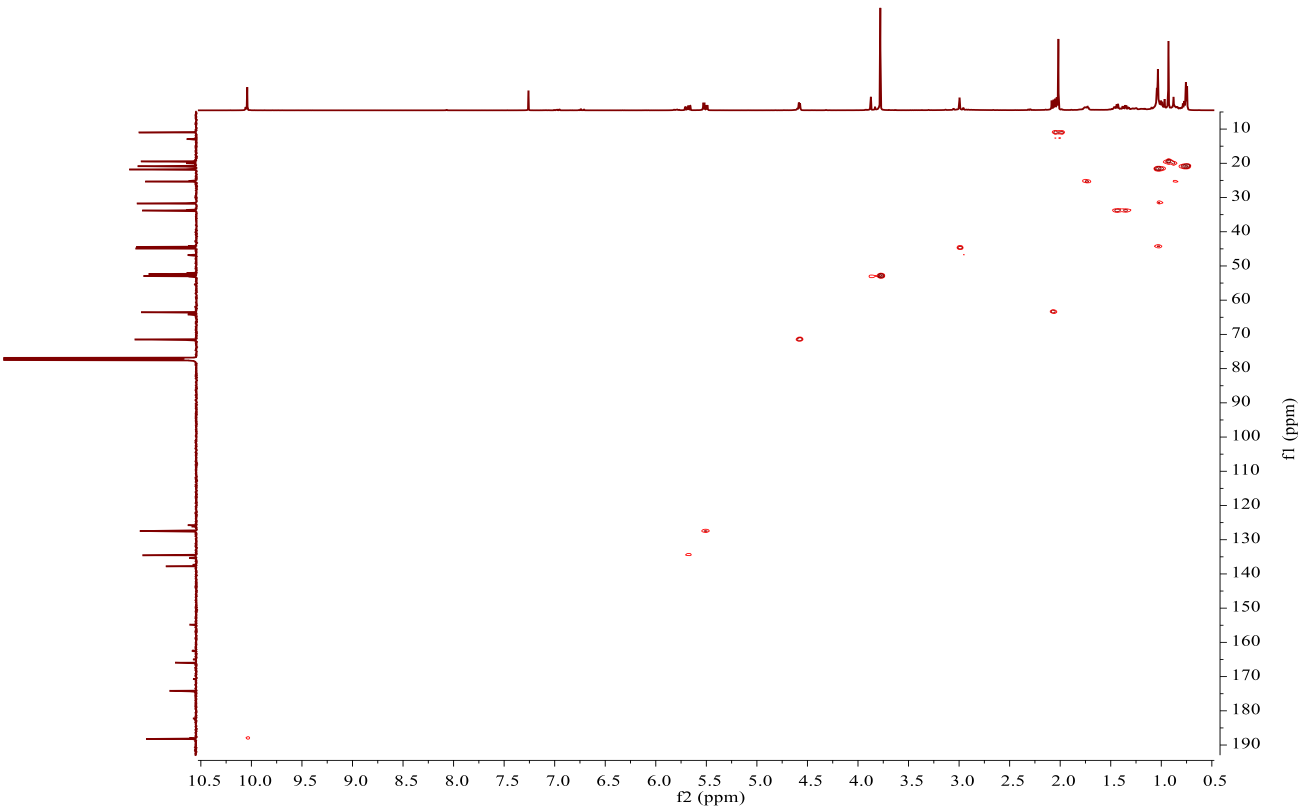

HMBC spectrum

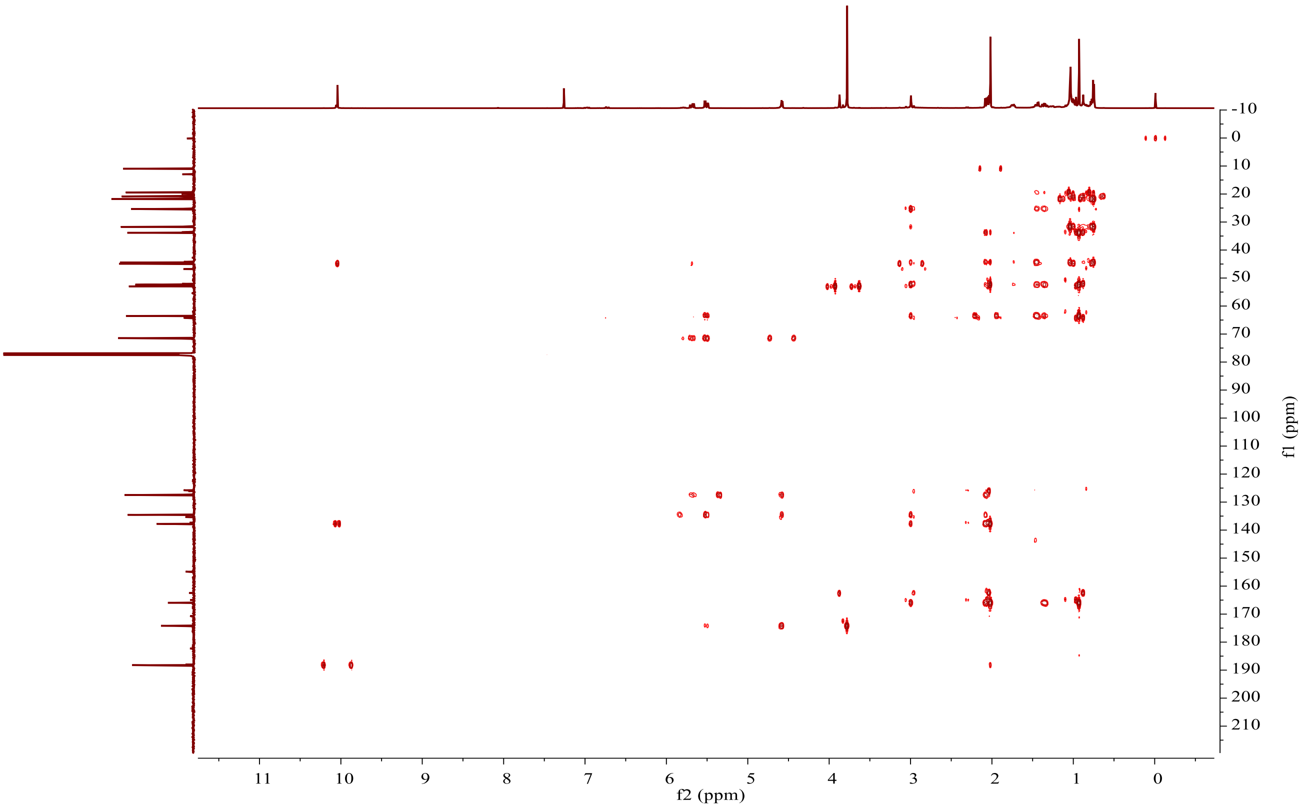

^1H-^1H COSY spectrum

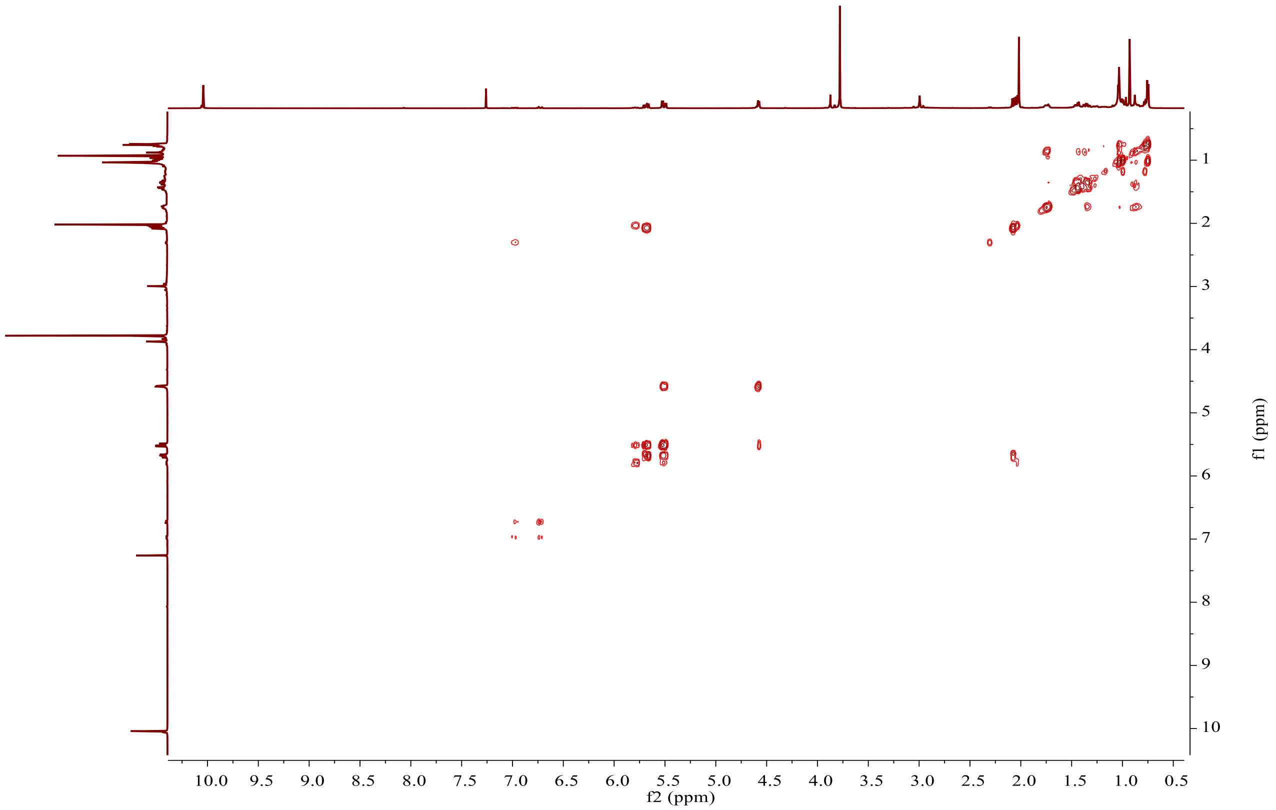

ROESY spectrum

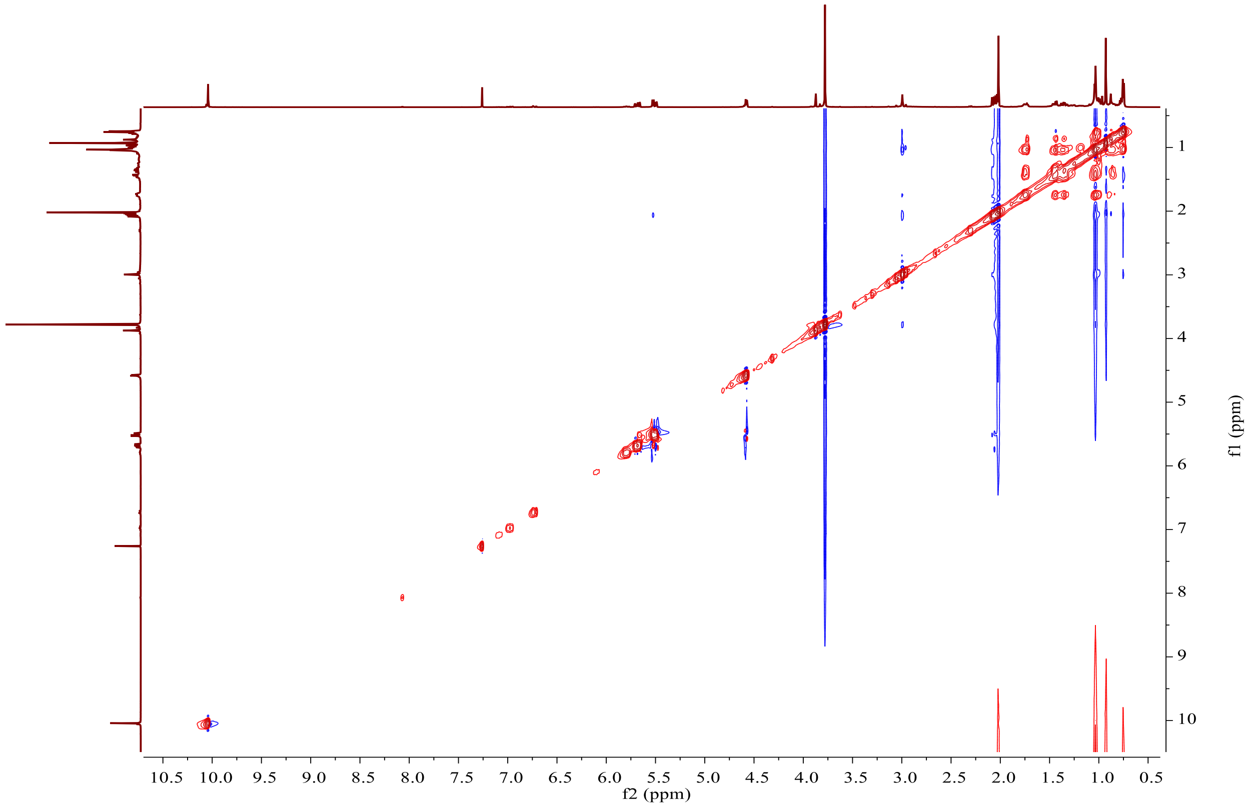

HRESIMS

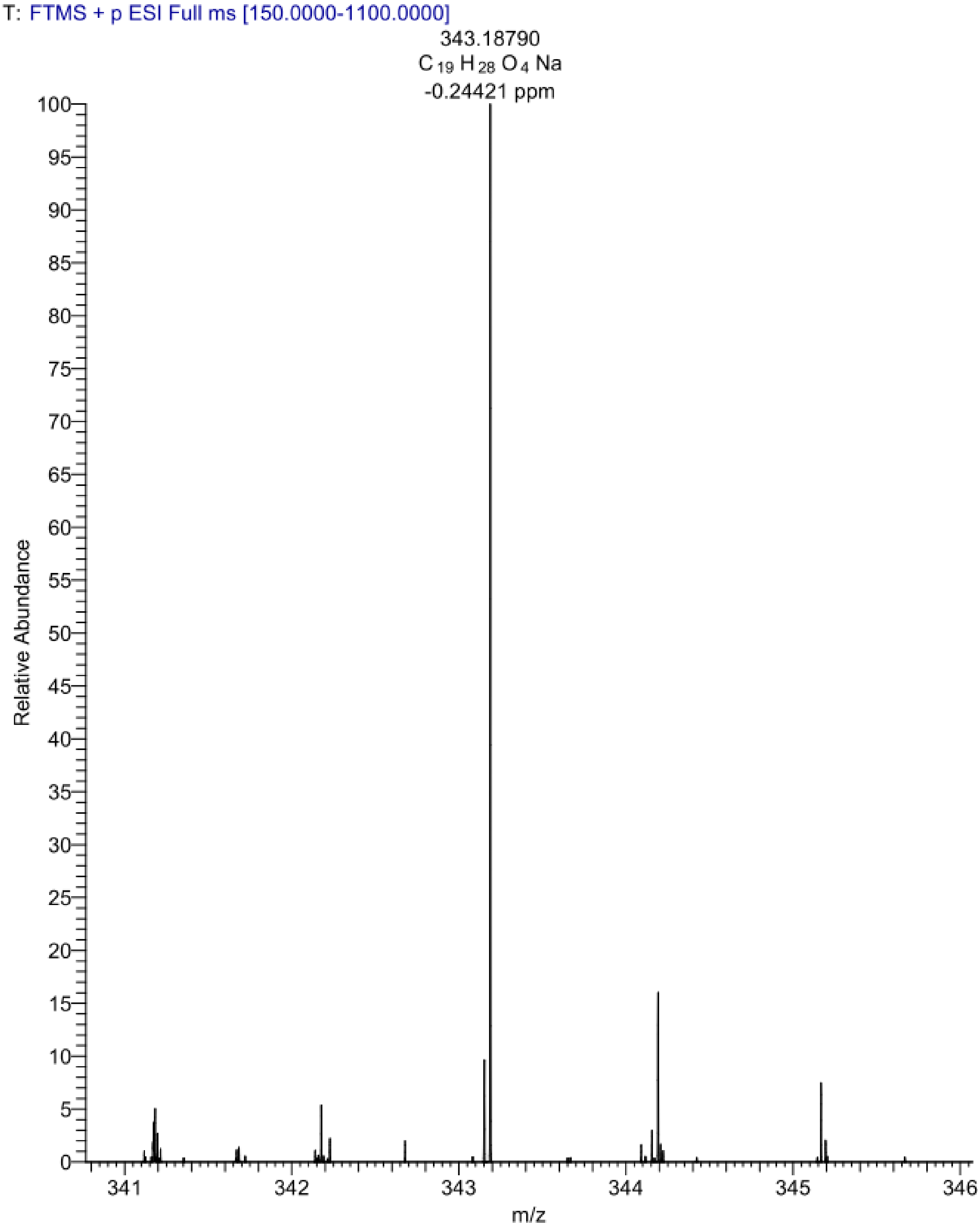

T: FTMS + p ESI Full ms [150.0000-1100.0000]
343.18790
C 19 H 28 O 4 Na
-0.24421 ppm
Relative Abundance
100
95
90
85
80
75
70
65
60
55
50
45
40
35
30
25
20
15
10
5
0
341
342
343
344
345
346
m/z

CD spectrum

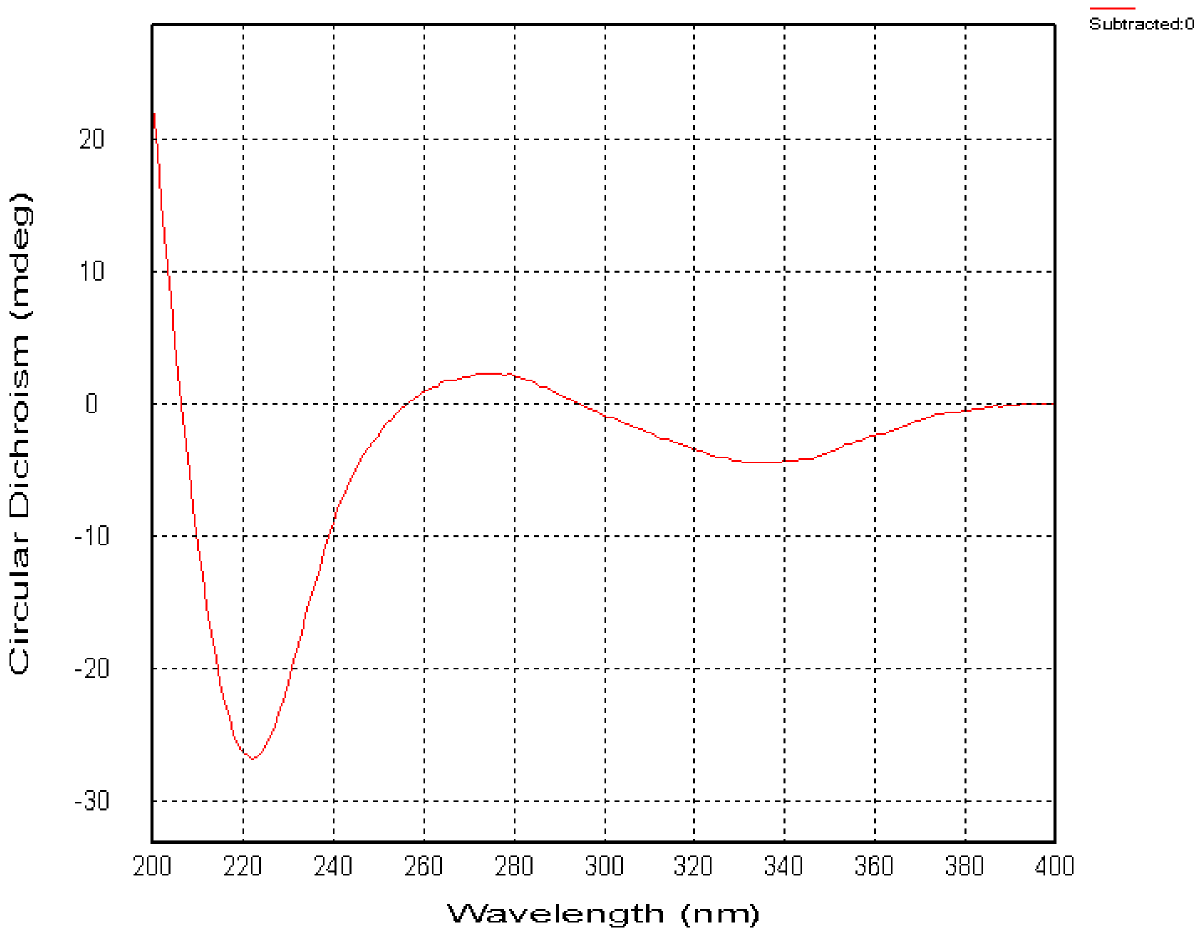

Subtracted:0
Circular Dichroism (mdeg)
20
10
0
-10
-20
-30
200
220
240
260
280
300
320
340
360
380
400
Wavelength (nm)

S1.2 NMR, HRESIMS and CD spectra of bipolarisorokin K

^{1}H NMR spectrum

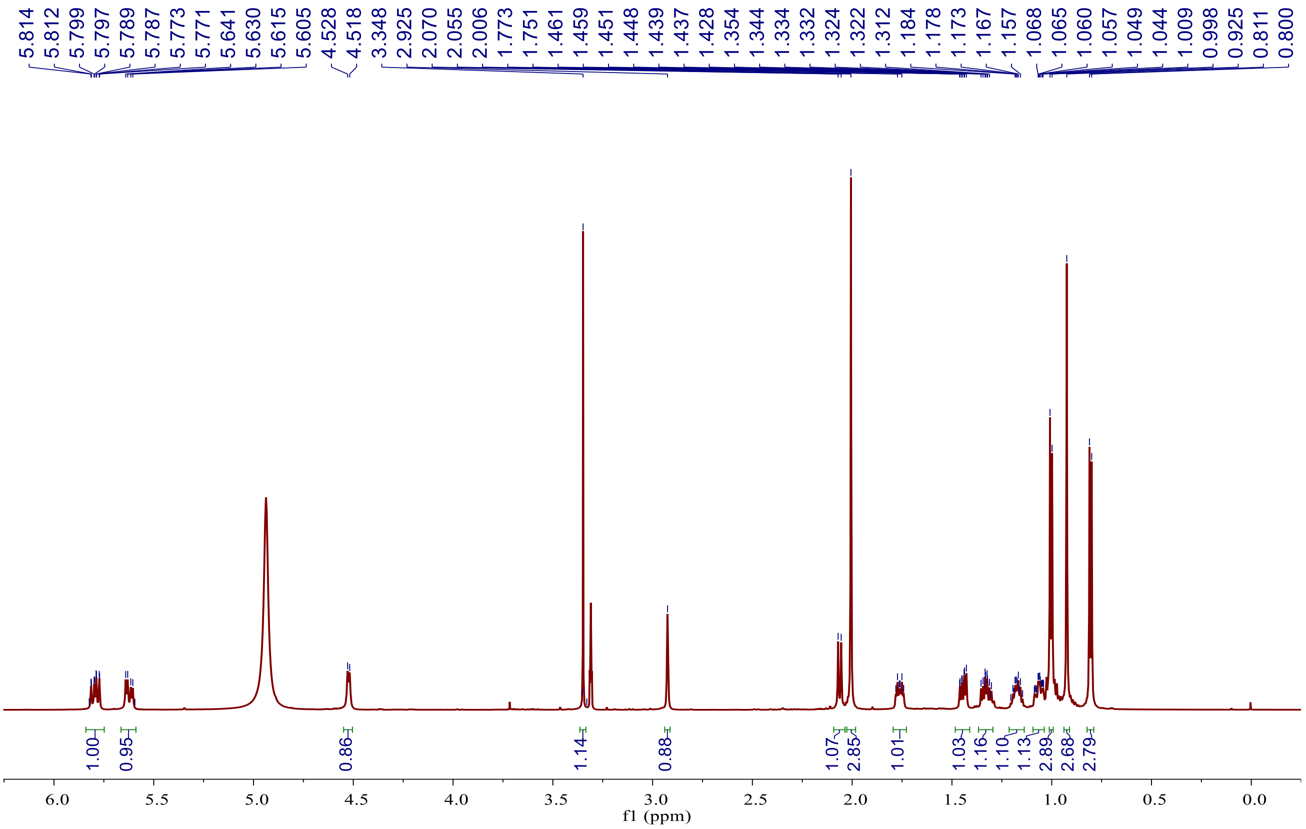

^{13}C NMR and DEPT spectra

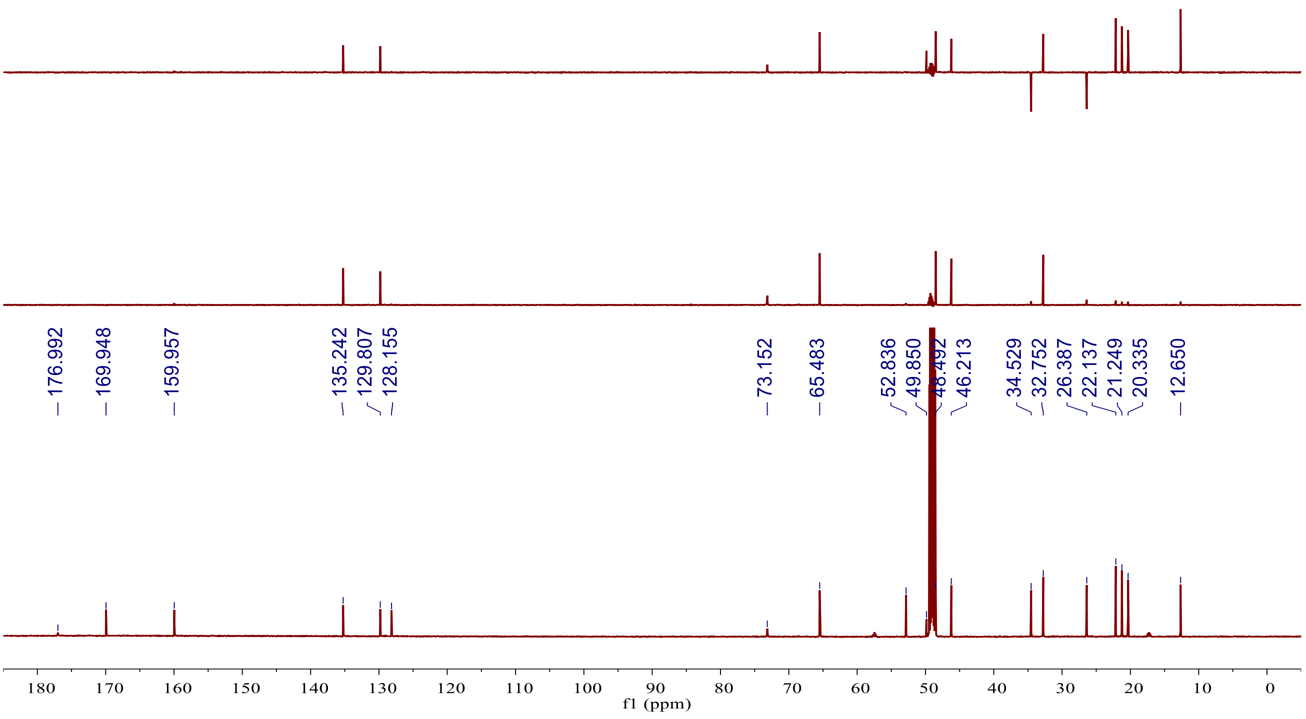

HSQC spectrum

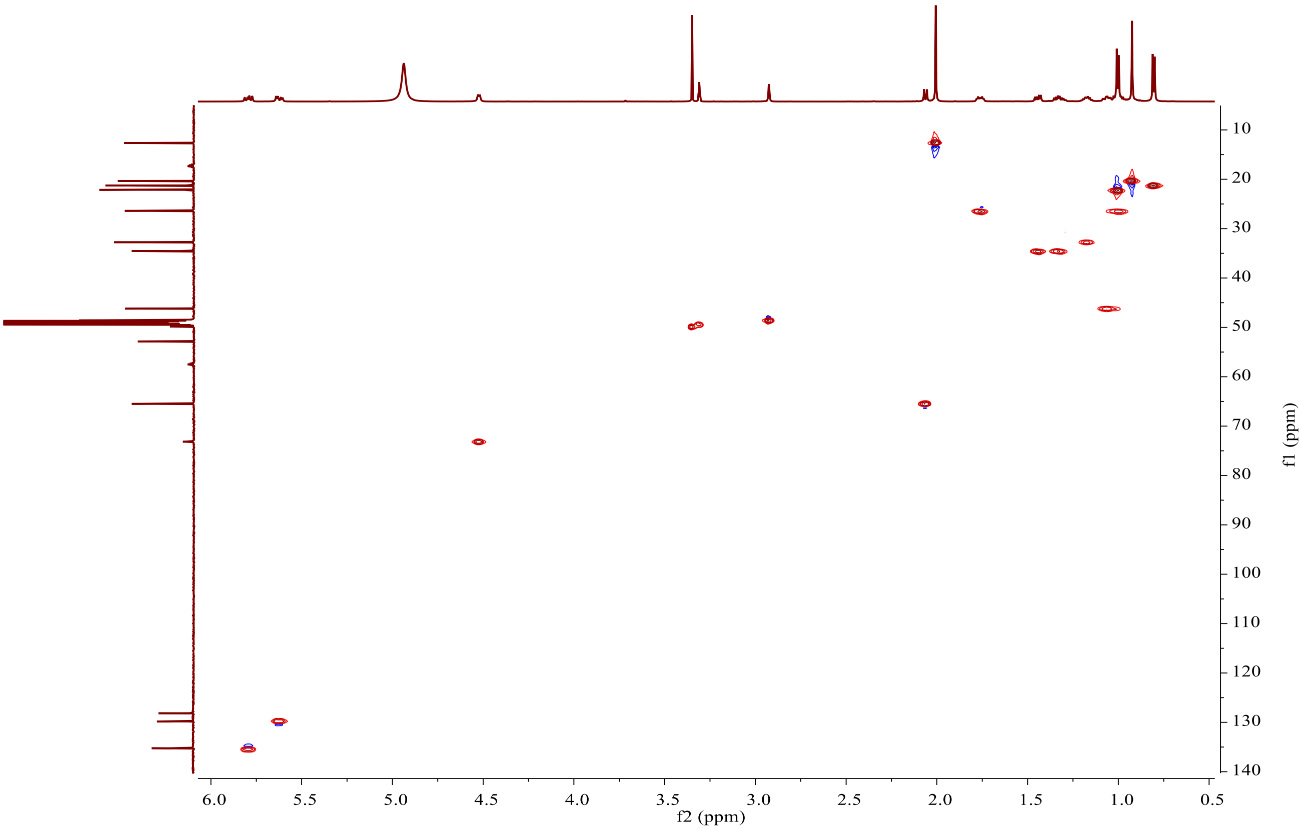

HMBC spectrum

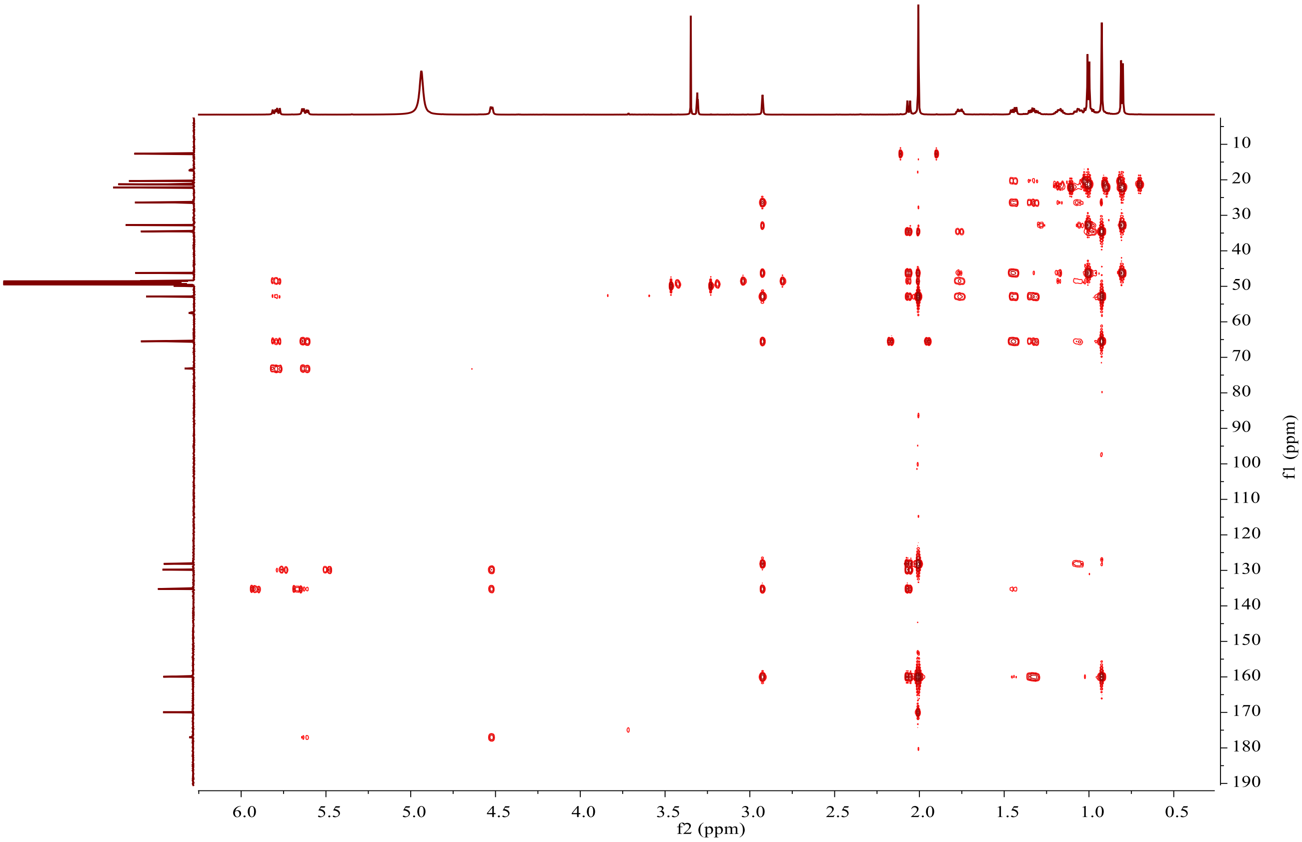

^{1}H-^{1}H COSY spectrum

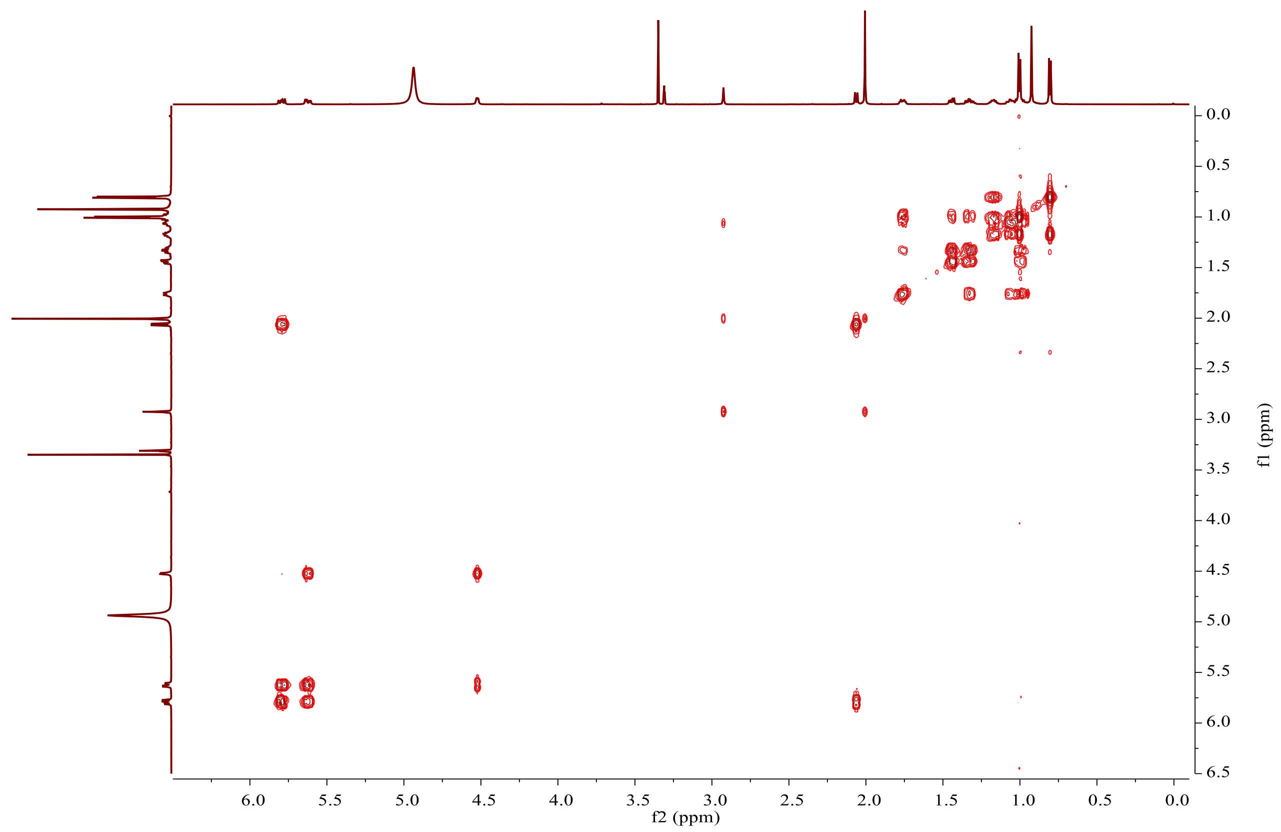

ROESY spectrum

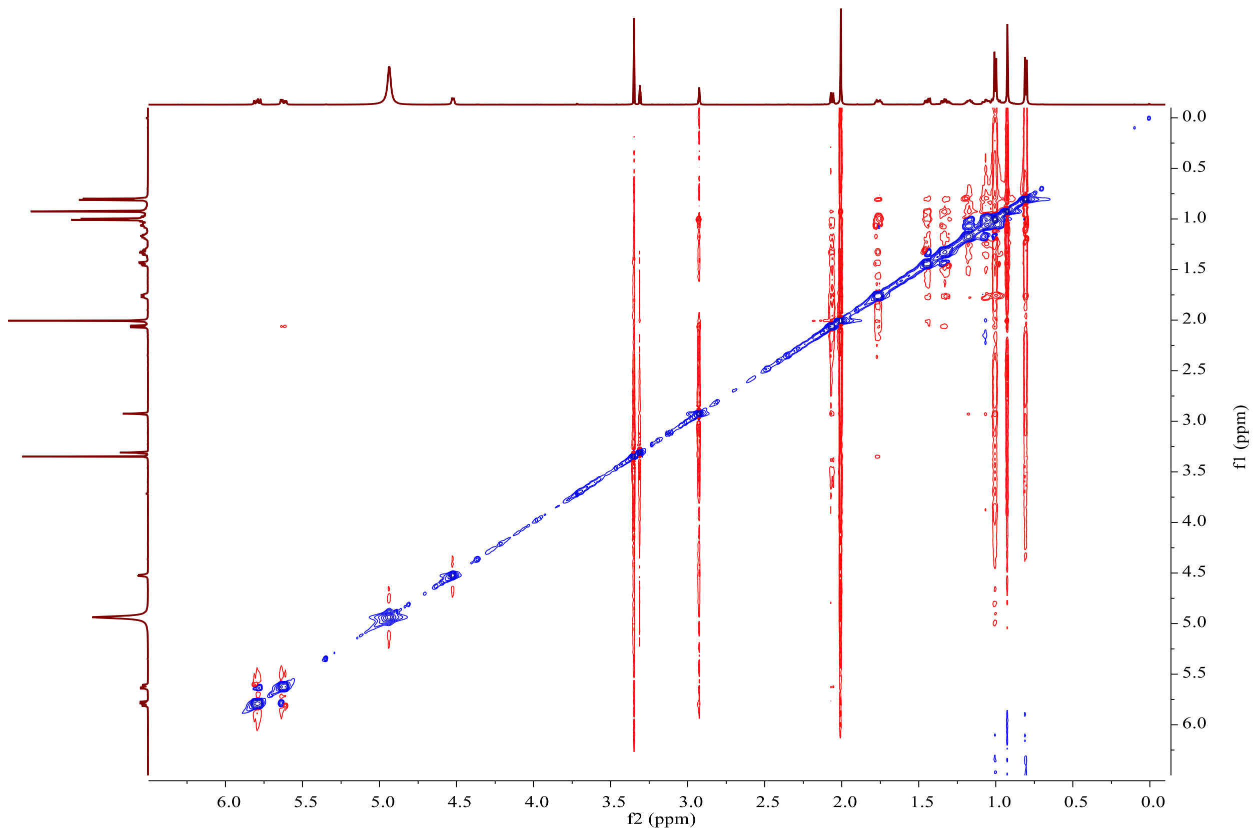

HRESIMS

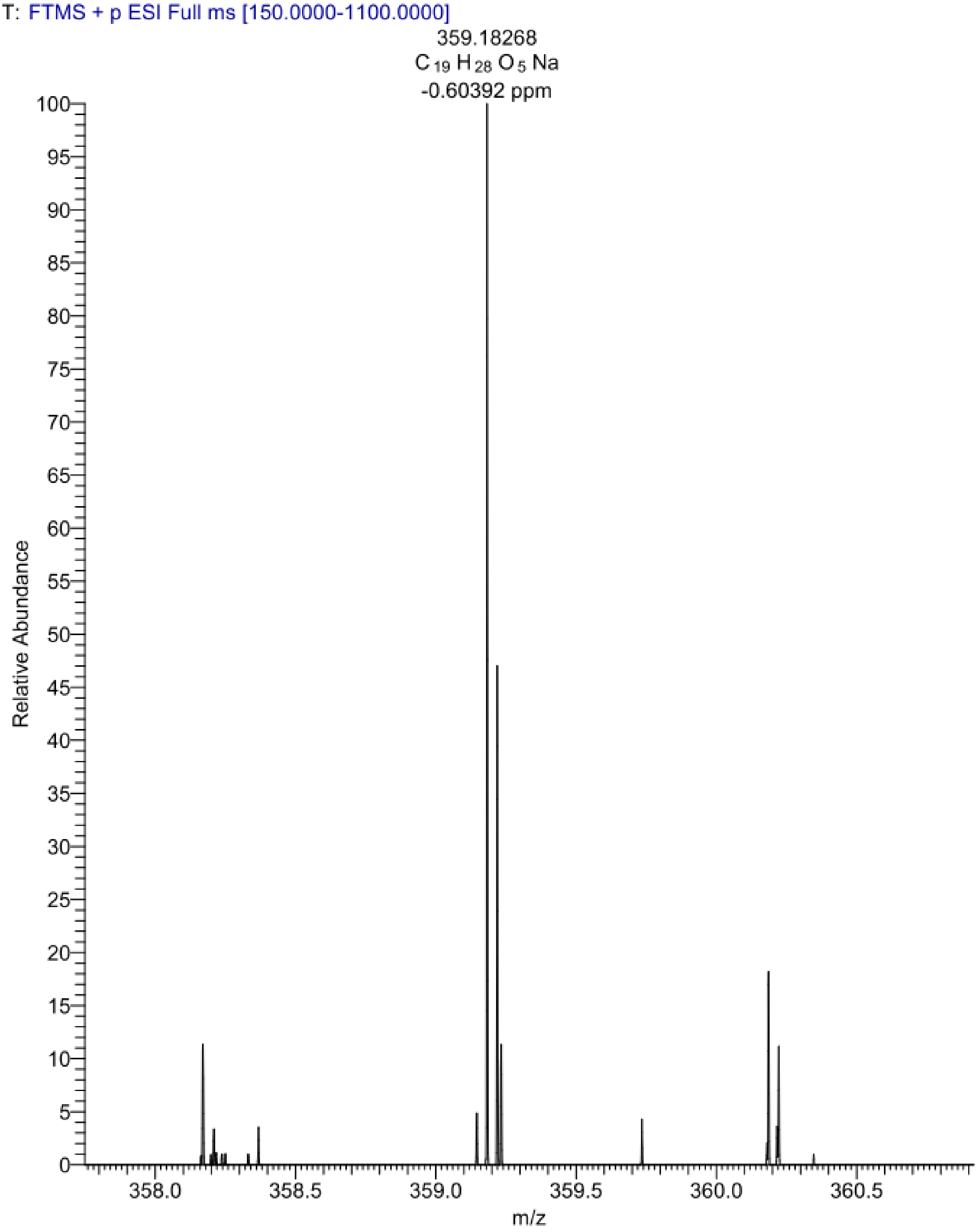

T: FTMS + p ESI Full ms [150.0000-1100.0000]
359.18268
C 19 H 28 O 5 Na
-0.60392 ppm
Relative Abundance
100
95
90
85
80
75
70
65
60
55
50
45
40
35
30
25
20
15
10
5
0
358.0
358.5
359.0
359.5
360.0
360.5
m/z

CD spectrum

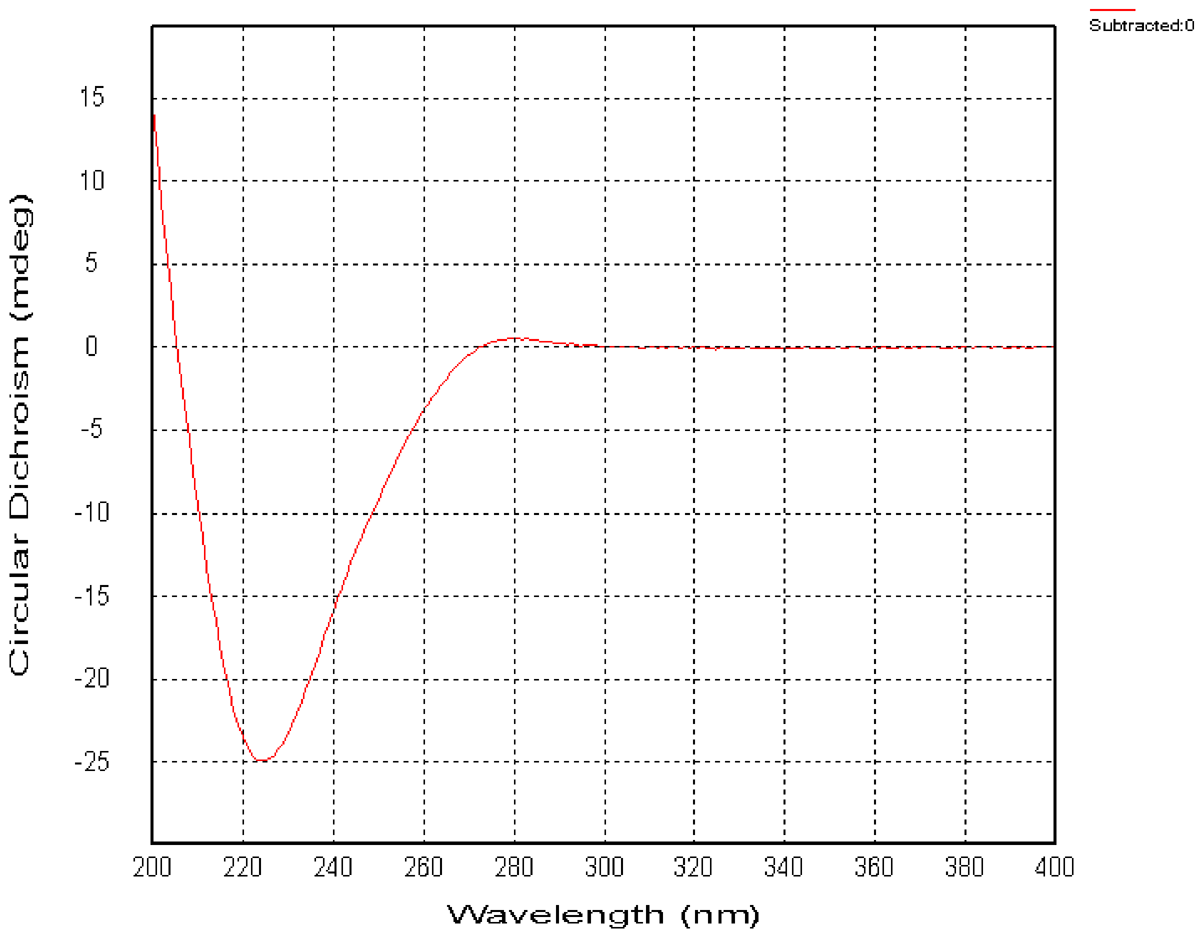

Subtracted:0
15
10
5
0
-5
-10
-15
-20
-25
Circular Dichroism (mdeg)
200
220
240
260
280
300
320
340
360
380
400
Wavelength (nm)

S1.3 NMR, HRESIMS and CD spectra of bipolarisorokin L

^{1}H NMR spectrum

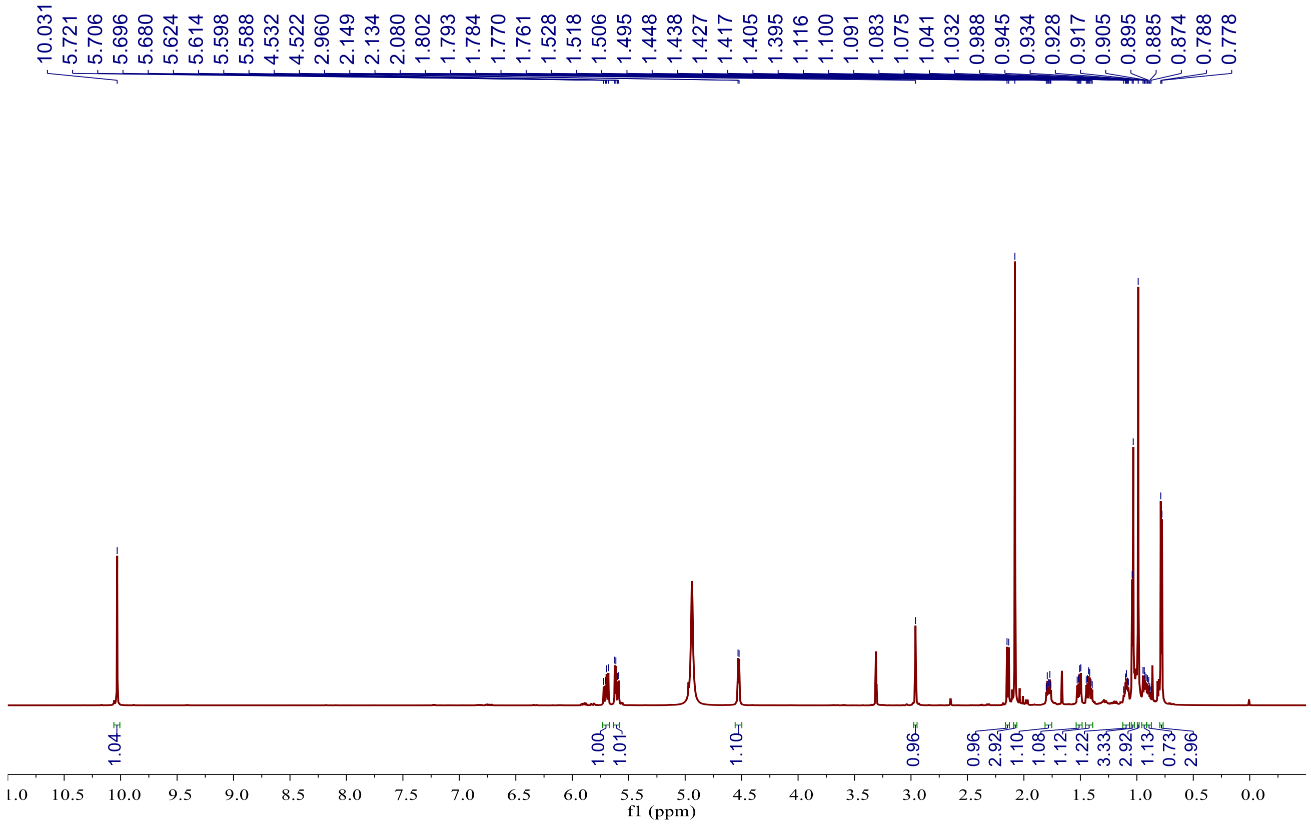

^{13}C NMR and DEPT spectra

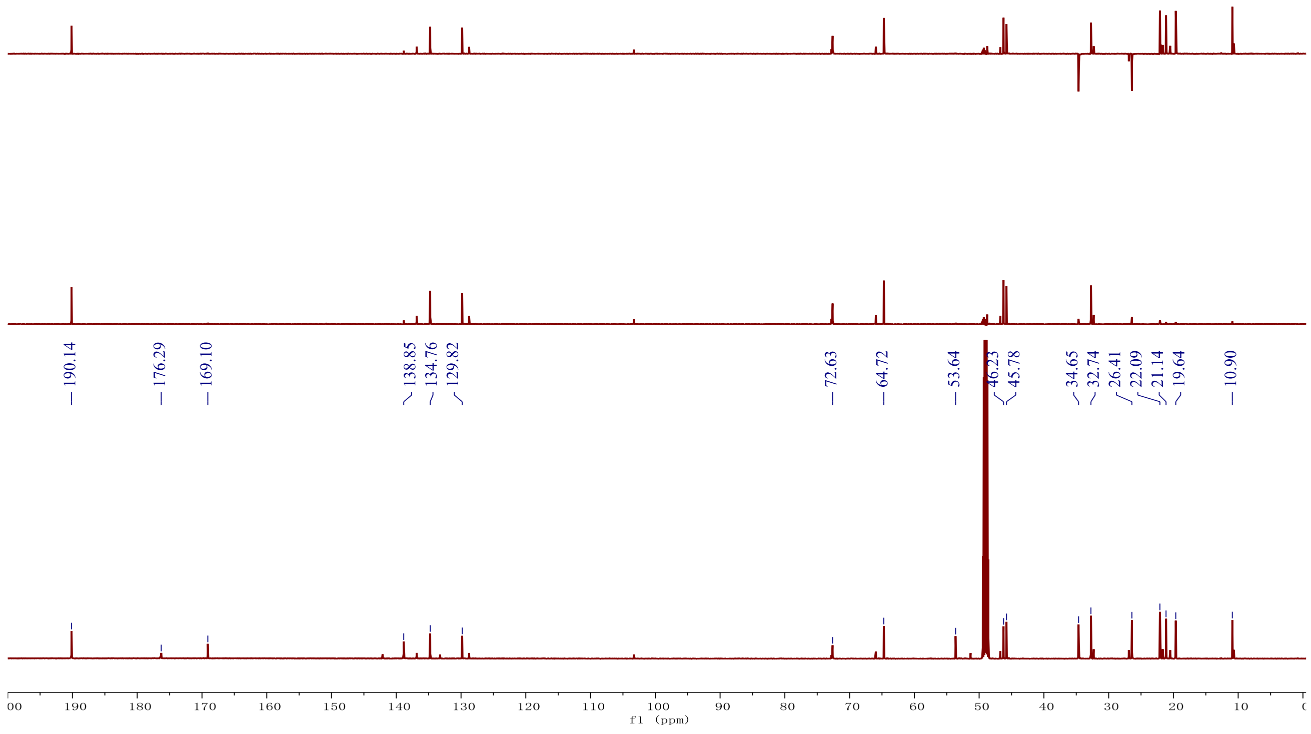

HSQC spectrum

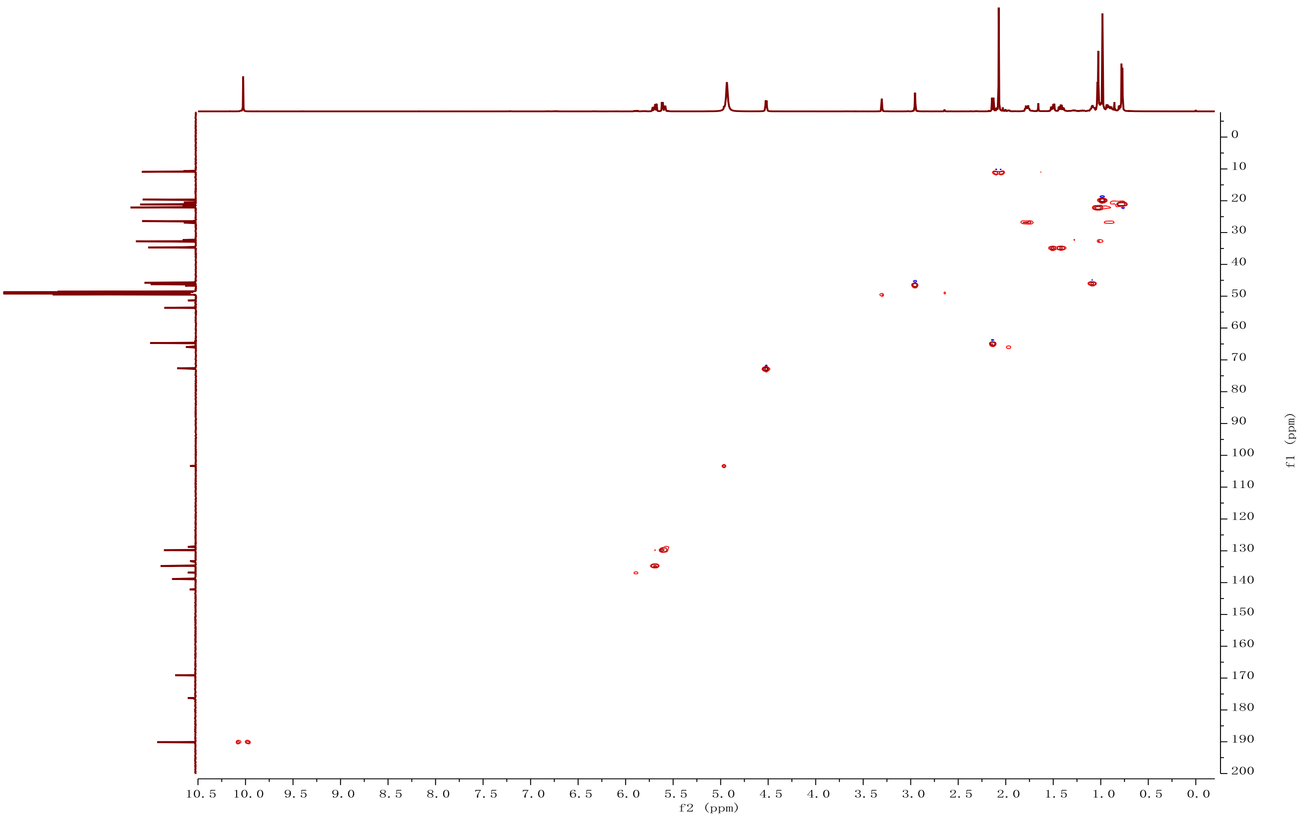

HMBC spectrum

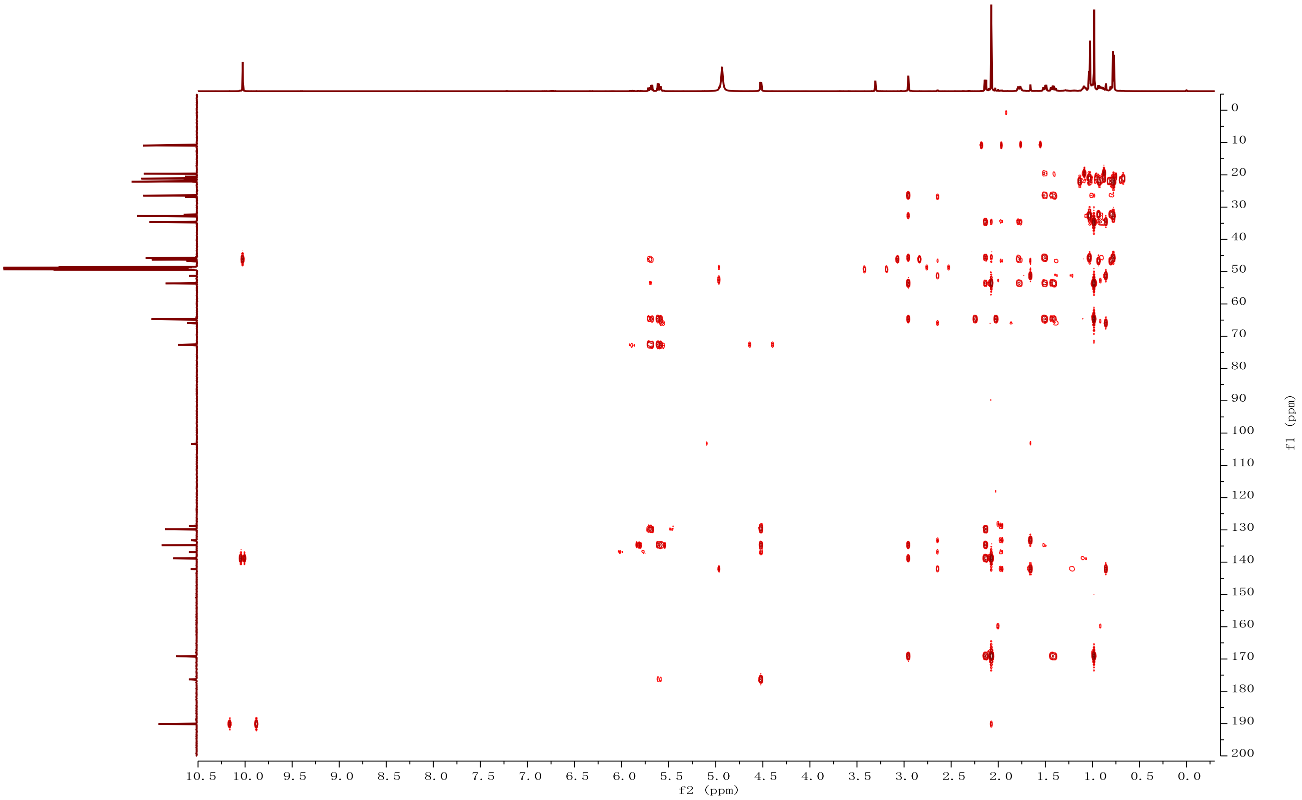

^{1}H-^{1}H COSY spectrum

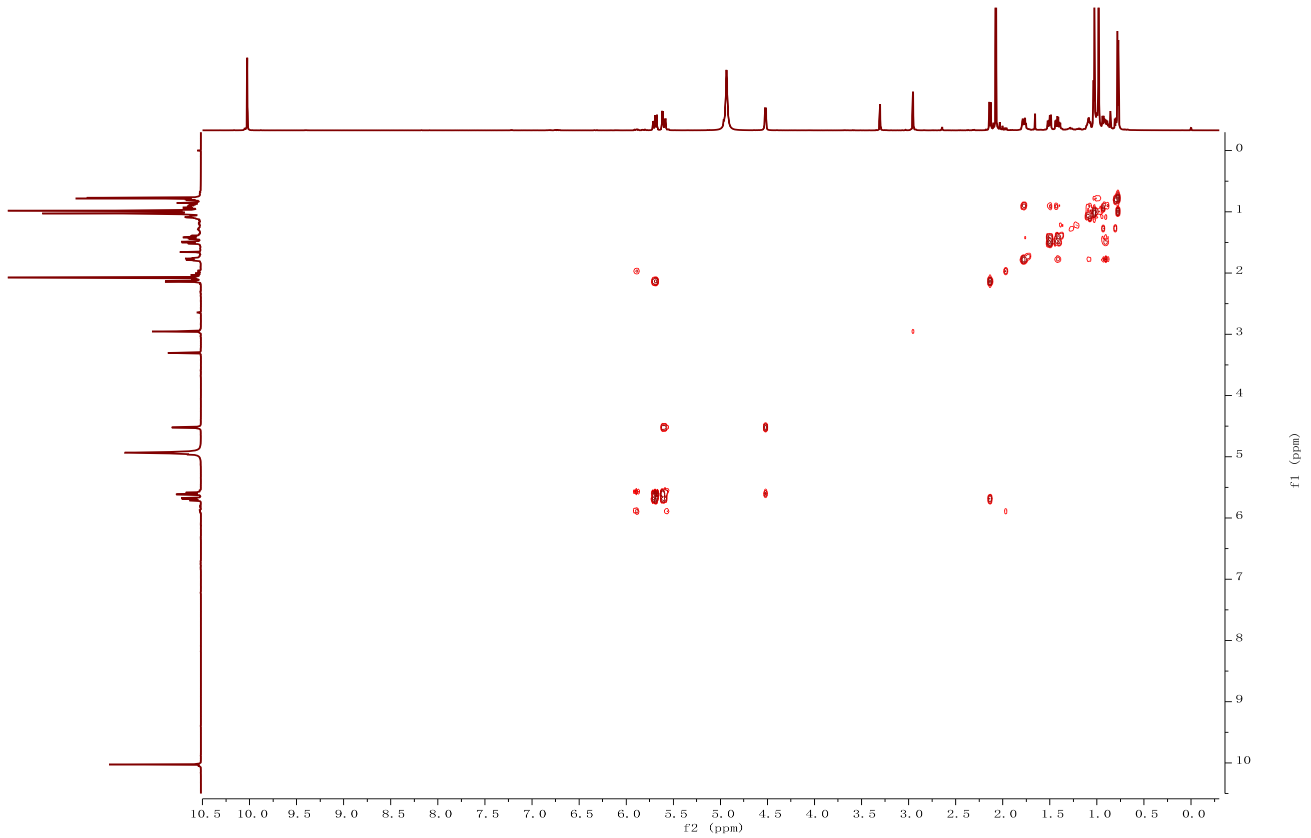

ROESY spectrum

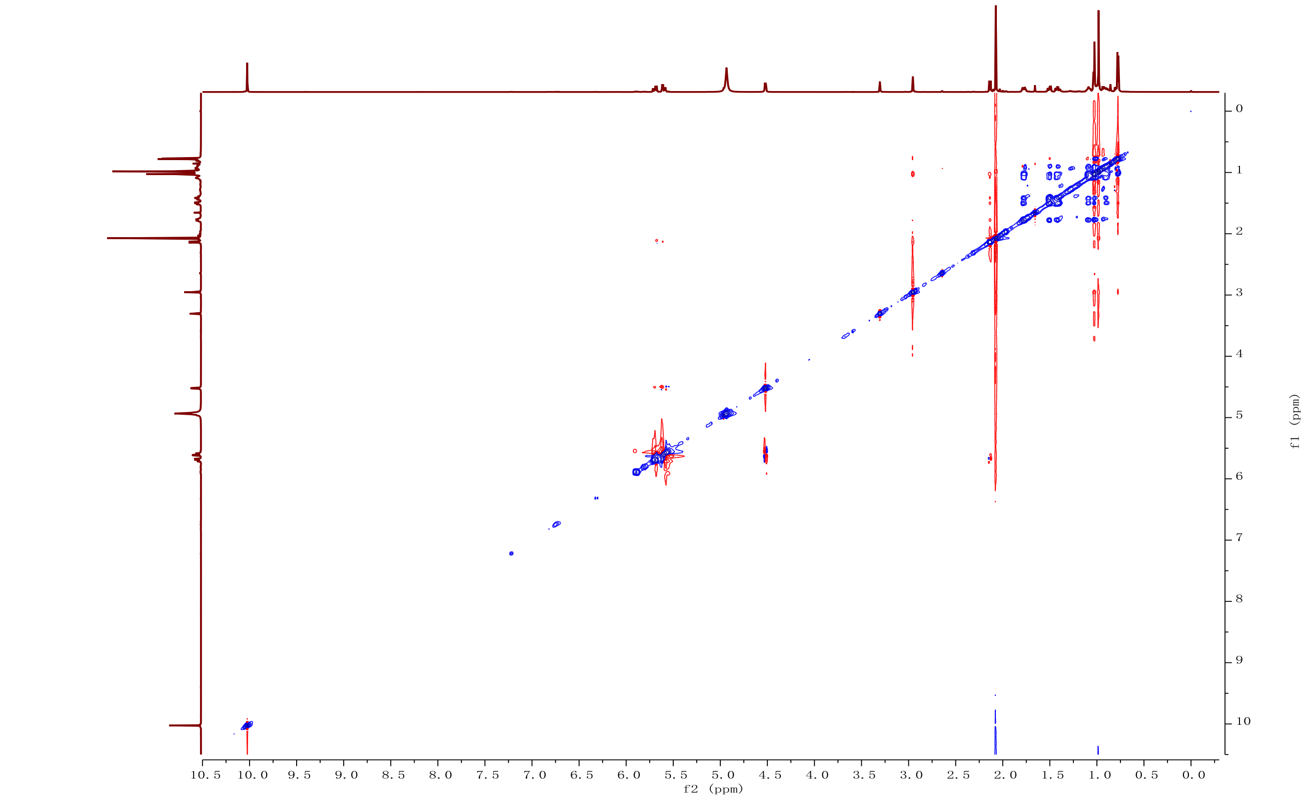

HRESIMS

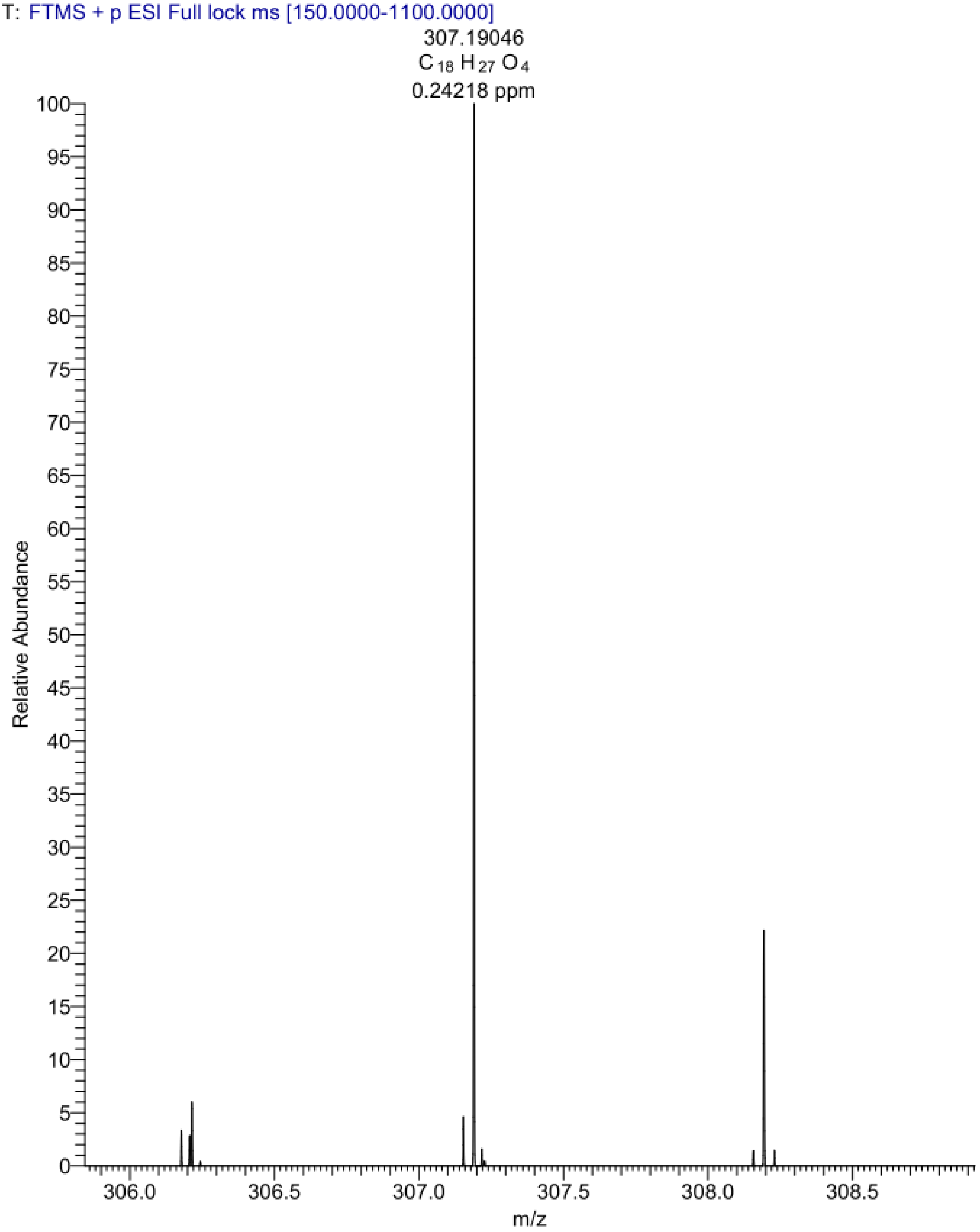
T: FTMS + p ESI Full lock ms [150.0000-1100.0000]
307.19046
C 18 H 27 O 4
0.24218 ppm
Relative Abundance
m/z
306.0
306.5
307.0
307.5
308.0
308.5

CD spectrum

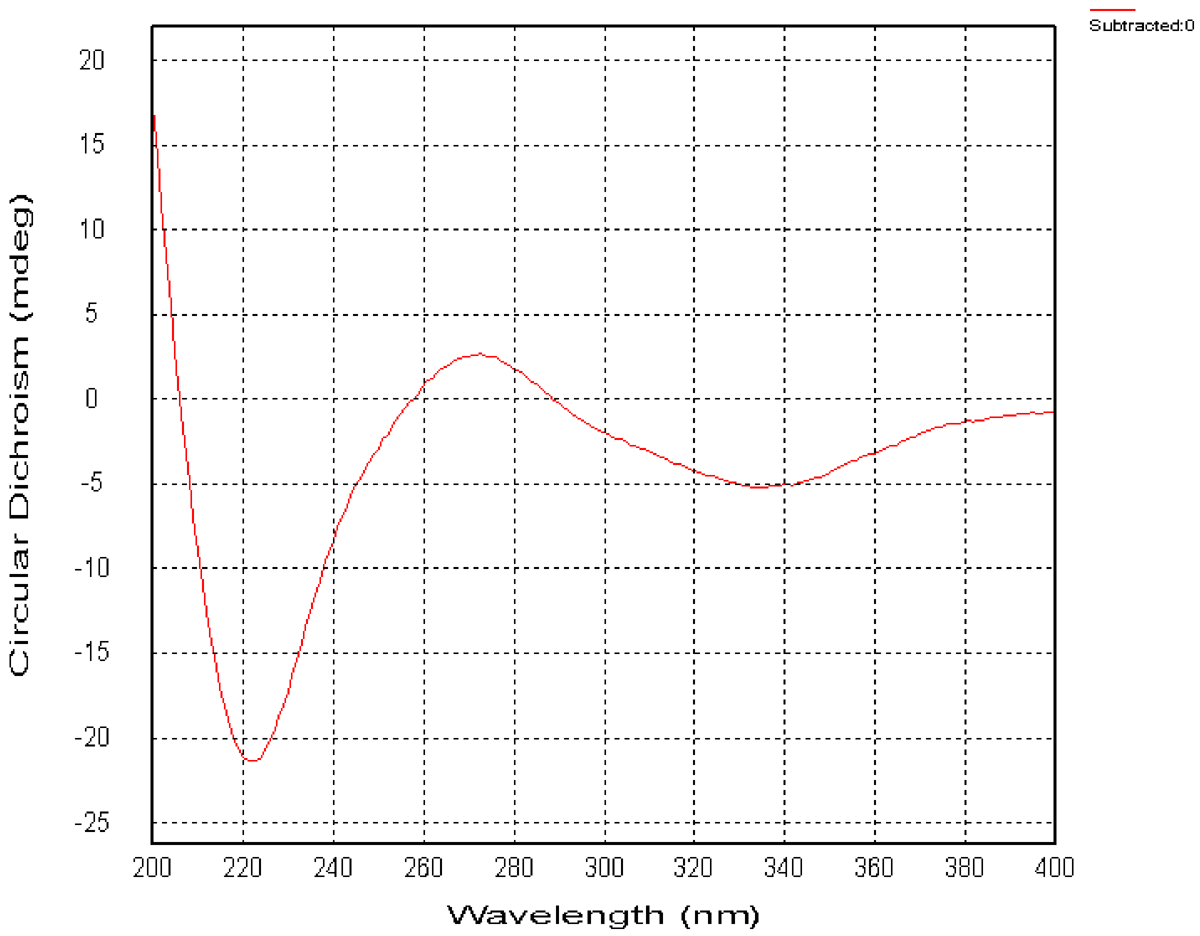
Subtracted:0
20
15
10
5
0
-5
-10
-15
-20
-25
Circular Dichroism (mdeg)
200
220
240
260
280
300
320
340
360
380
400
Wavelength (nm)

S1.4 NMR, HRESIMS and CD spectra of bipolarisorokin M

^{1}H NMR spectrum

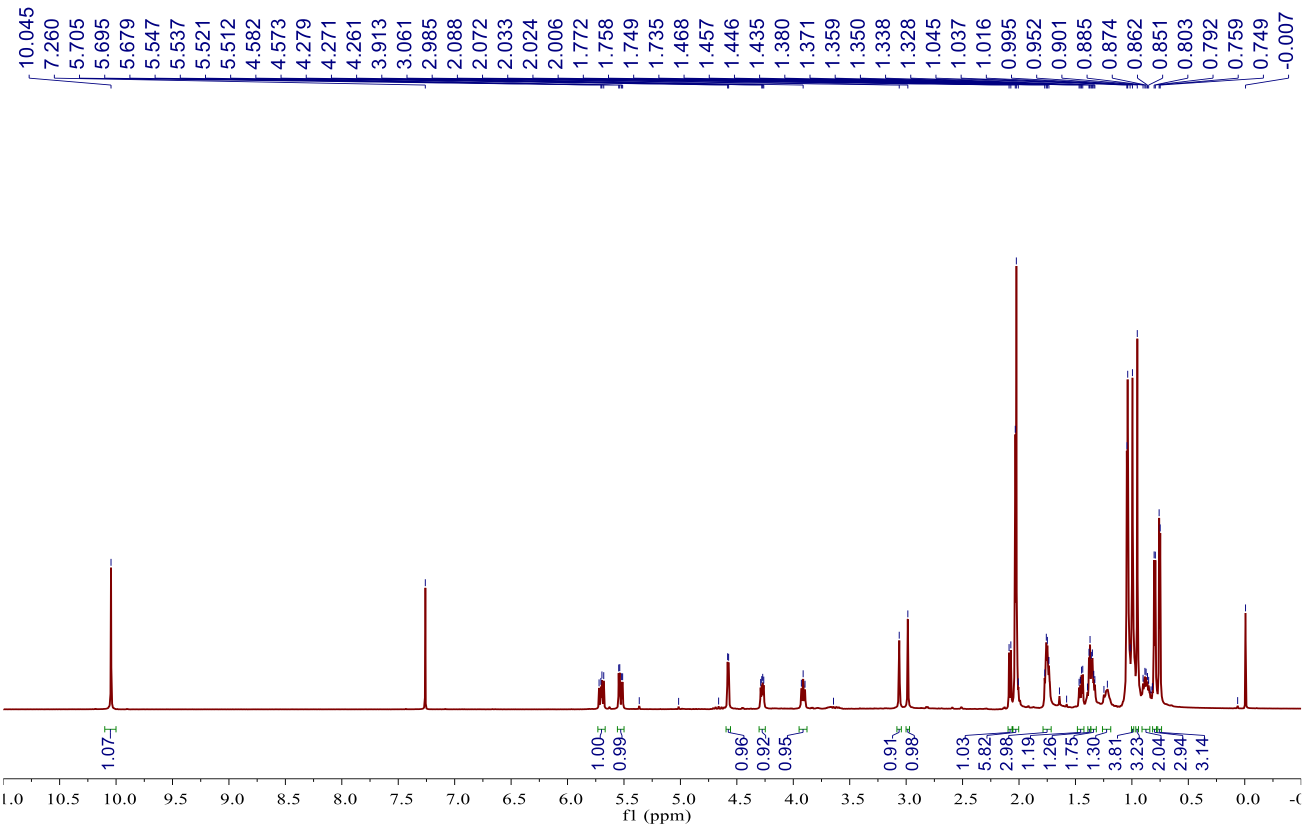

^{13}C NMR and DEPT spectra

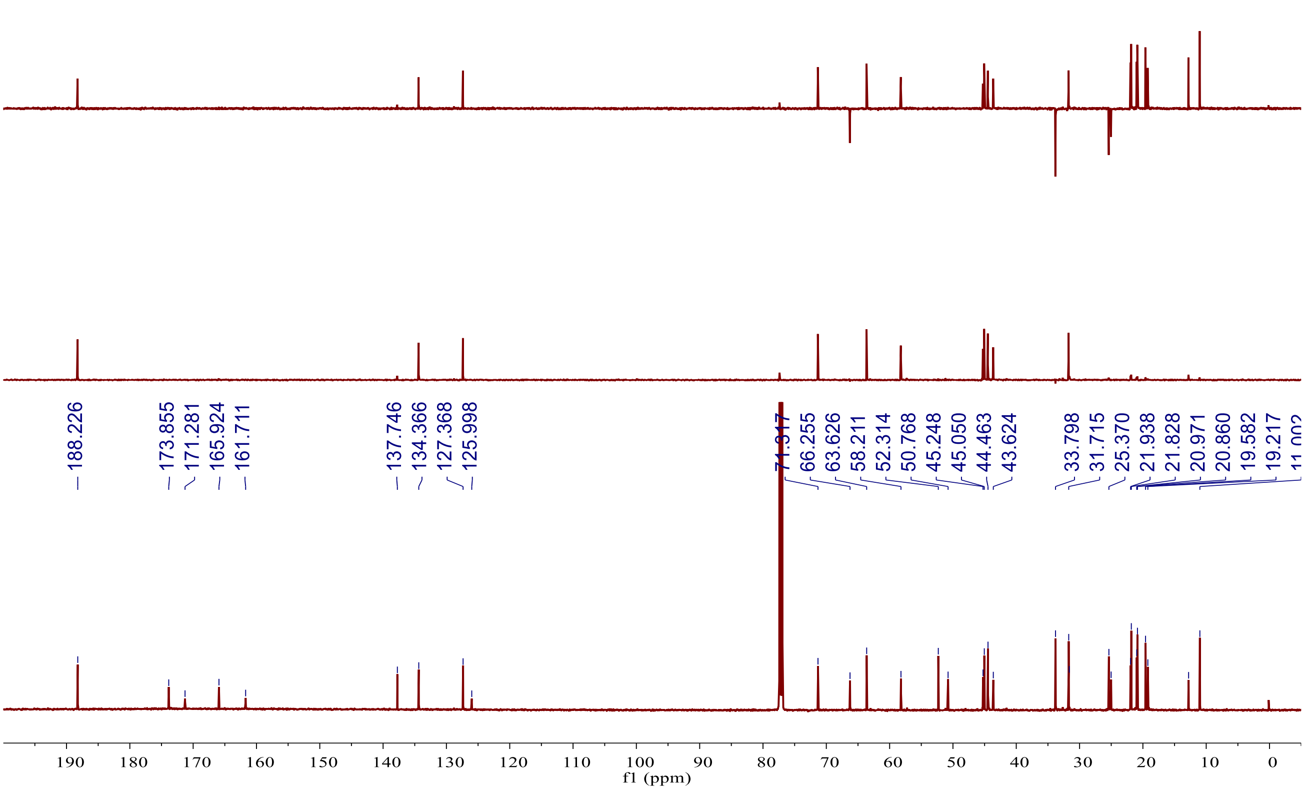

HSQC spectrum

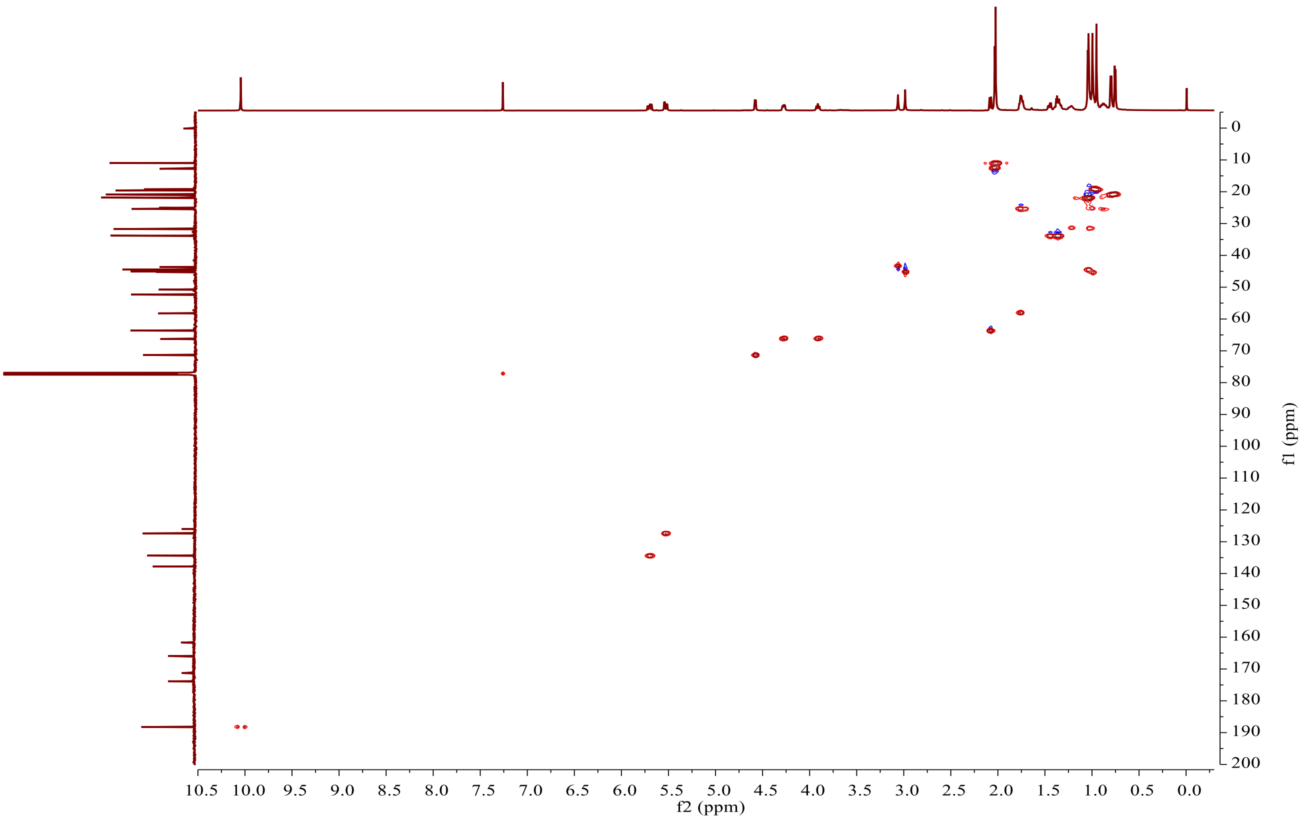

HMBC spectrum

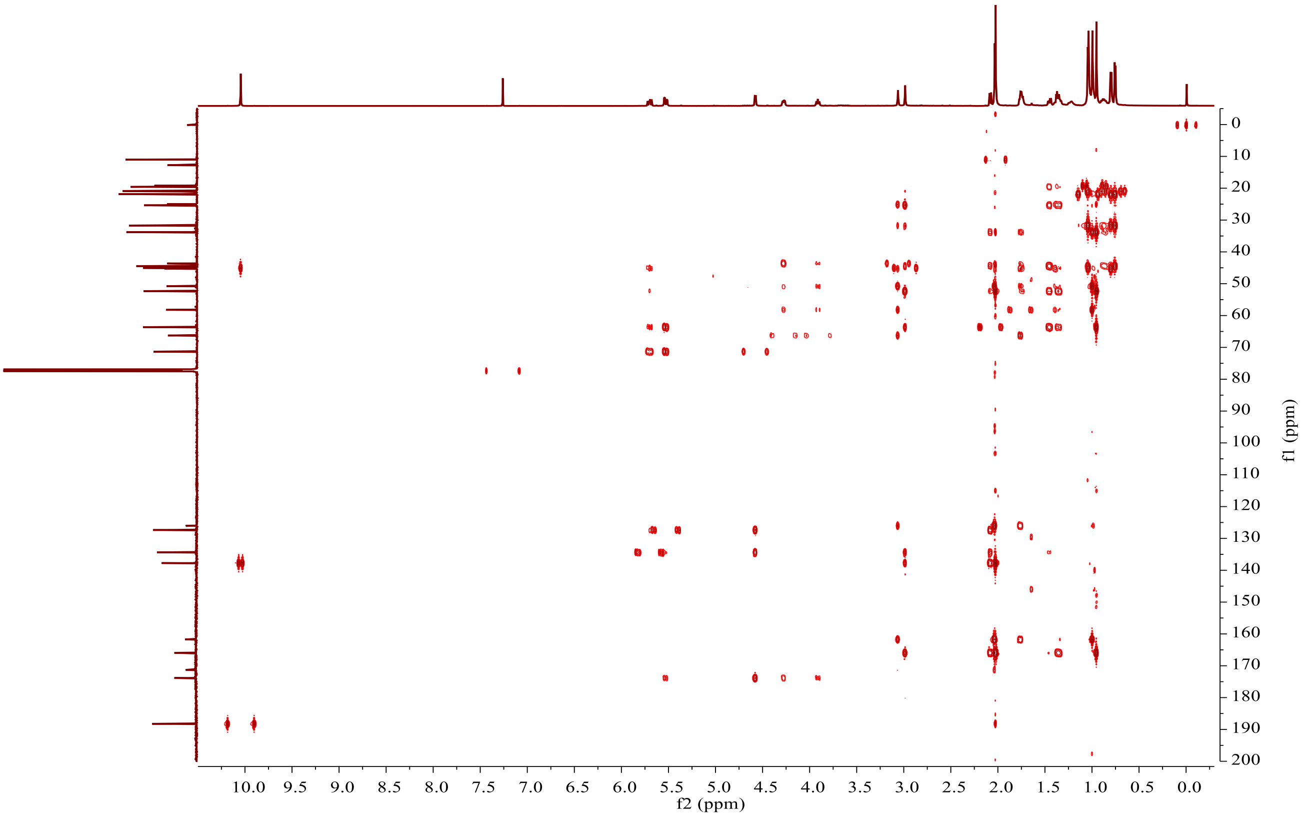

^{1}H-^{1}H COSY spectrum

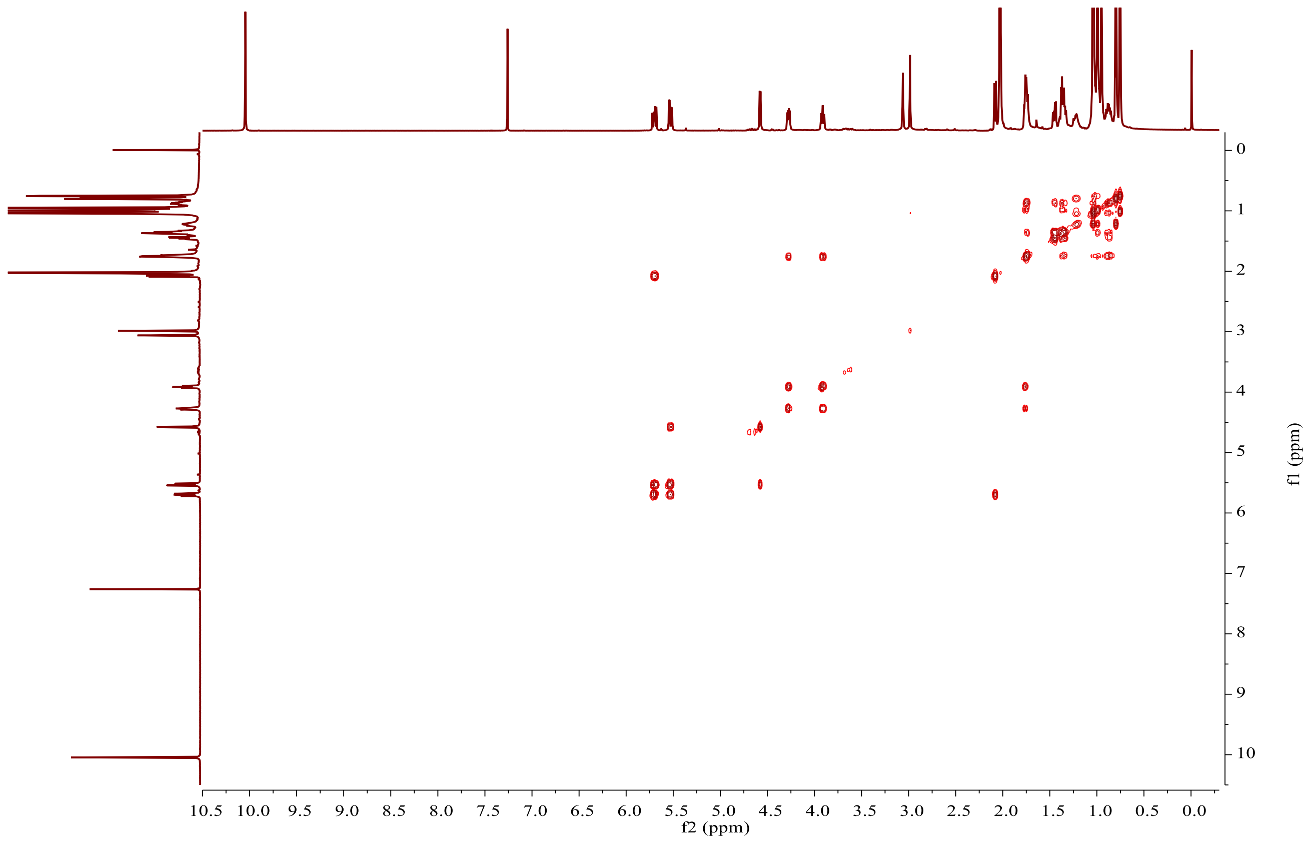

ROESY spectrum

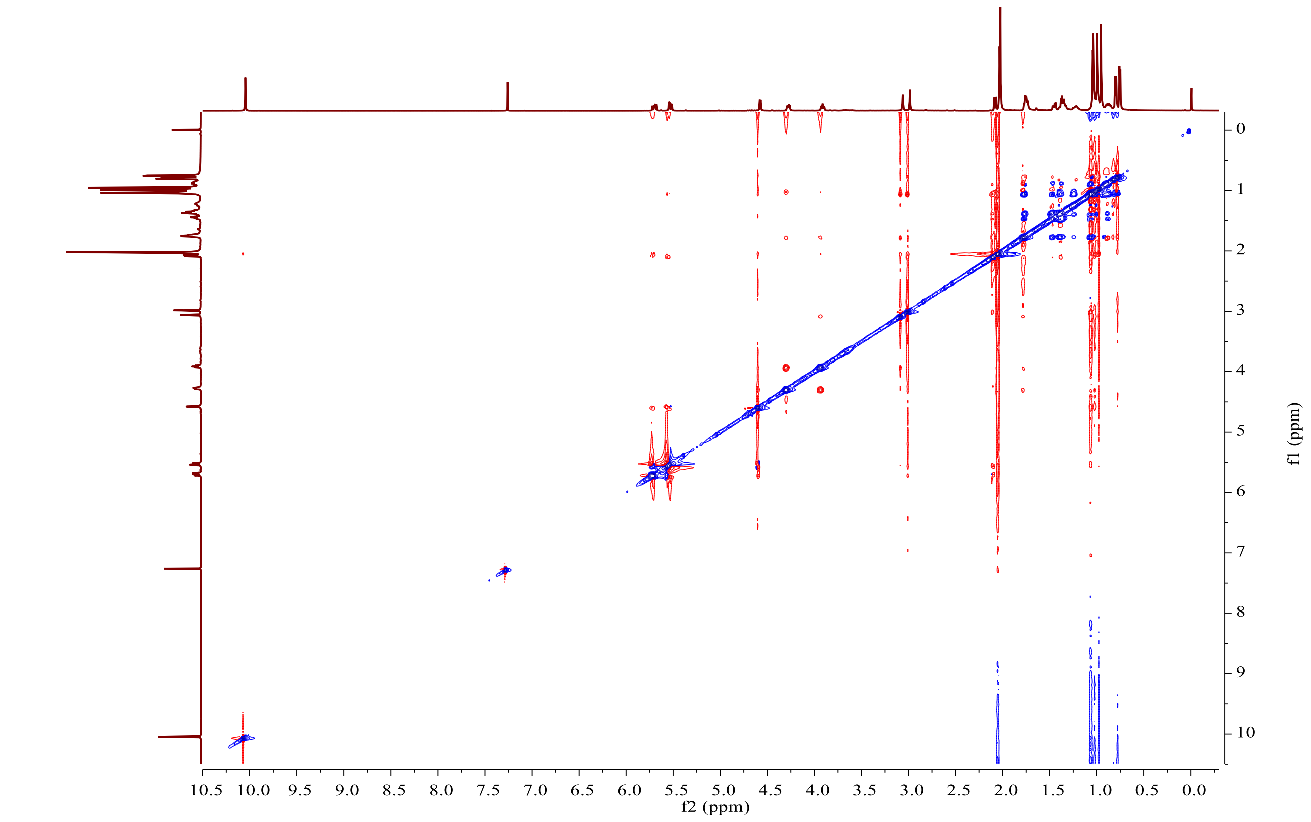

HRESIMS

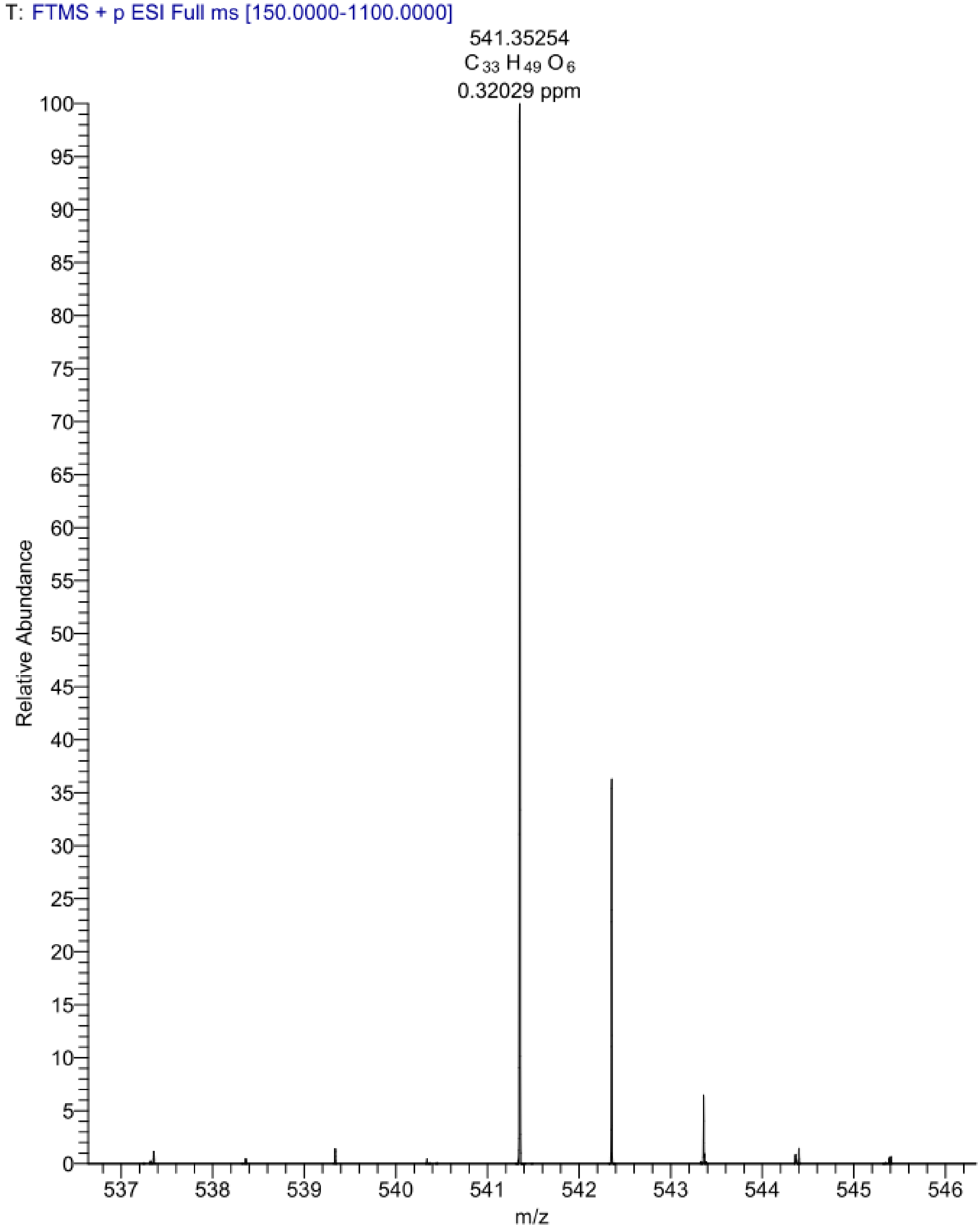

T: FTMS + p ESI Full ms [150.0000-1100.0000]
541.35254
C 33 H 49 O 6
0.32029 ppm
Relative Abundance
m/z

CD spectrum

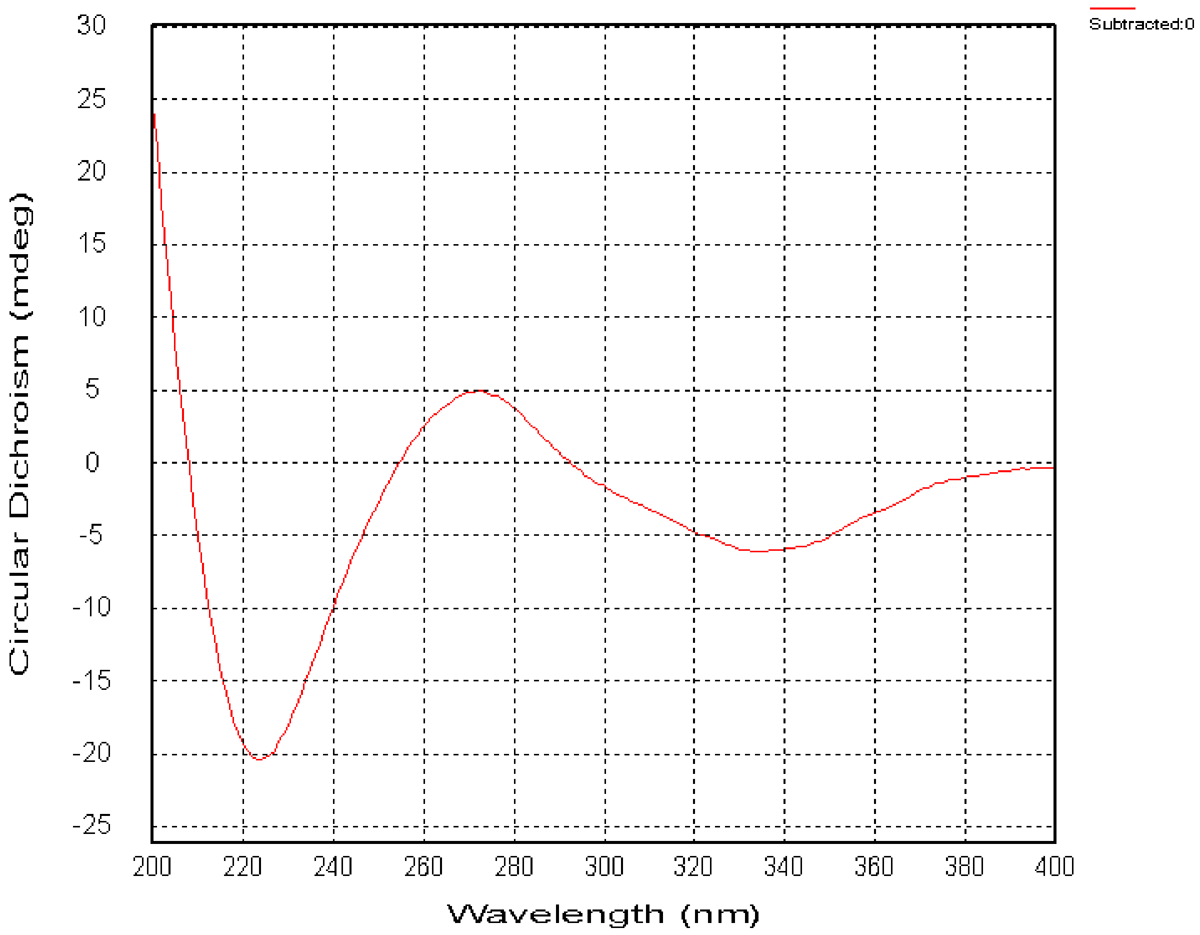
Subtracted:0
Circular Dichroism (mdeg)
30
25
20
15
10
5
0
-5
-10
-15
-20
-25
200
220
240
260
280
300
320
340
360
380
400
Wavelength (nm)

S1.5 NMR, HRESIMS and CD spectra of bipolarisorokin N

^{1}H NMR spectrum

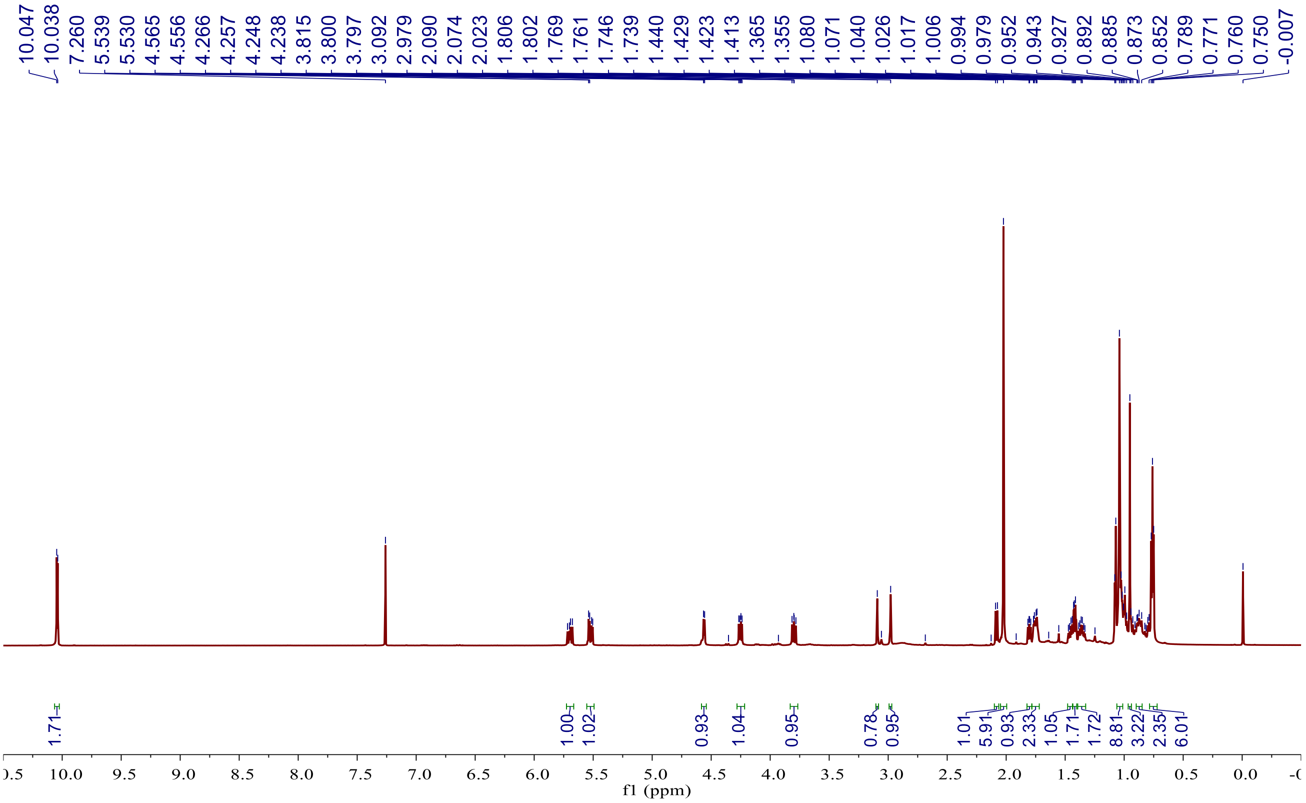

^{13}C NMR and DEPT spectra

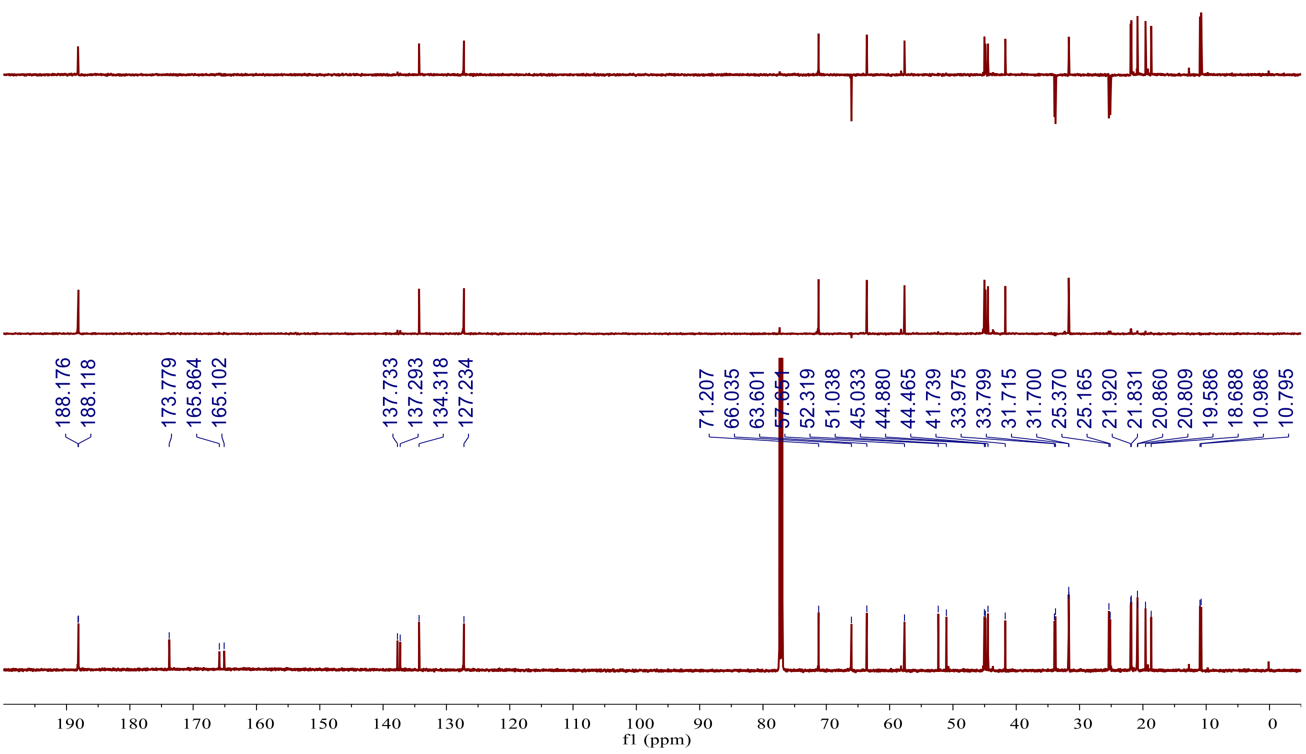

HSQC spectrum

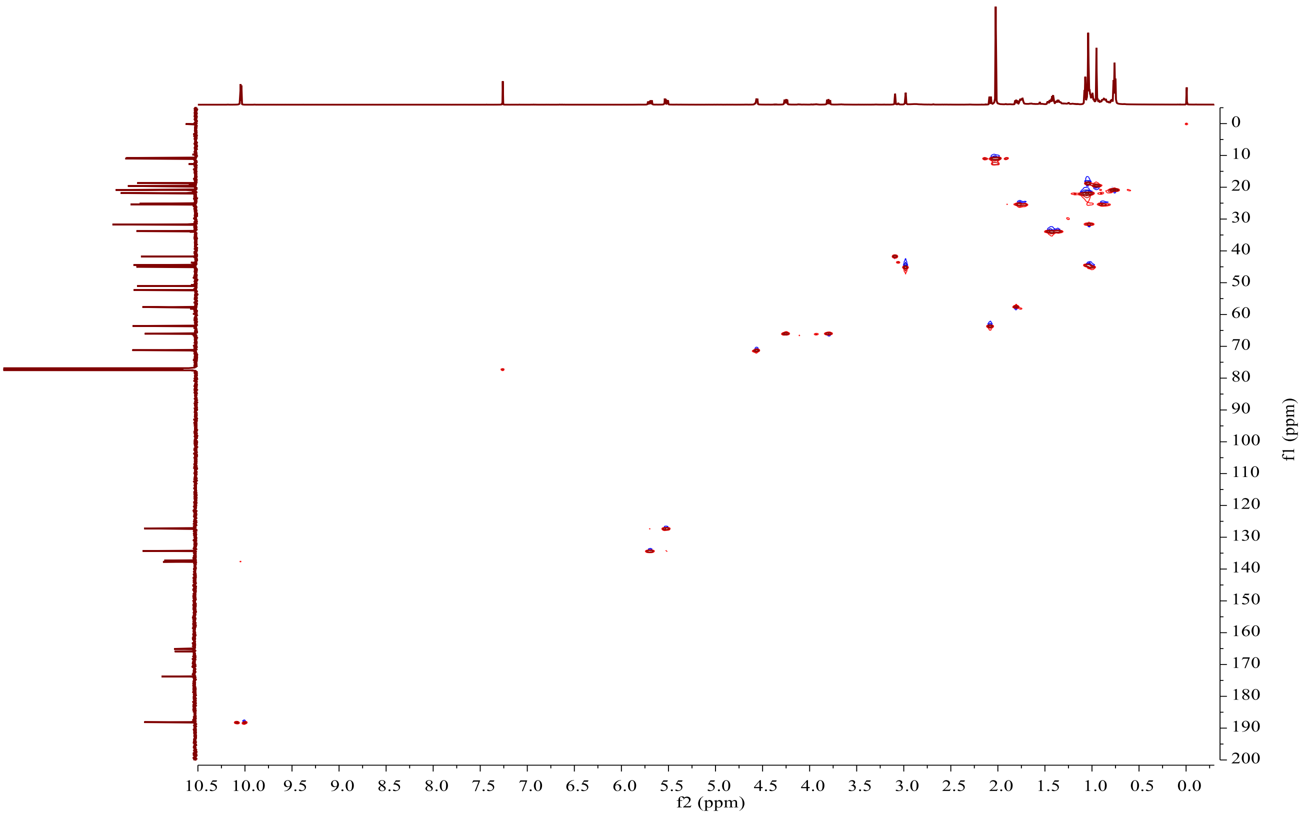

HMBC spectrum

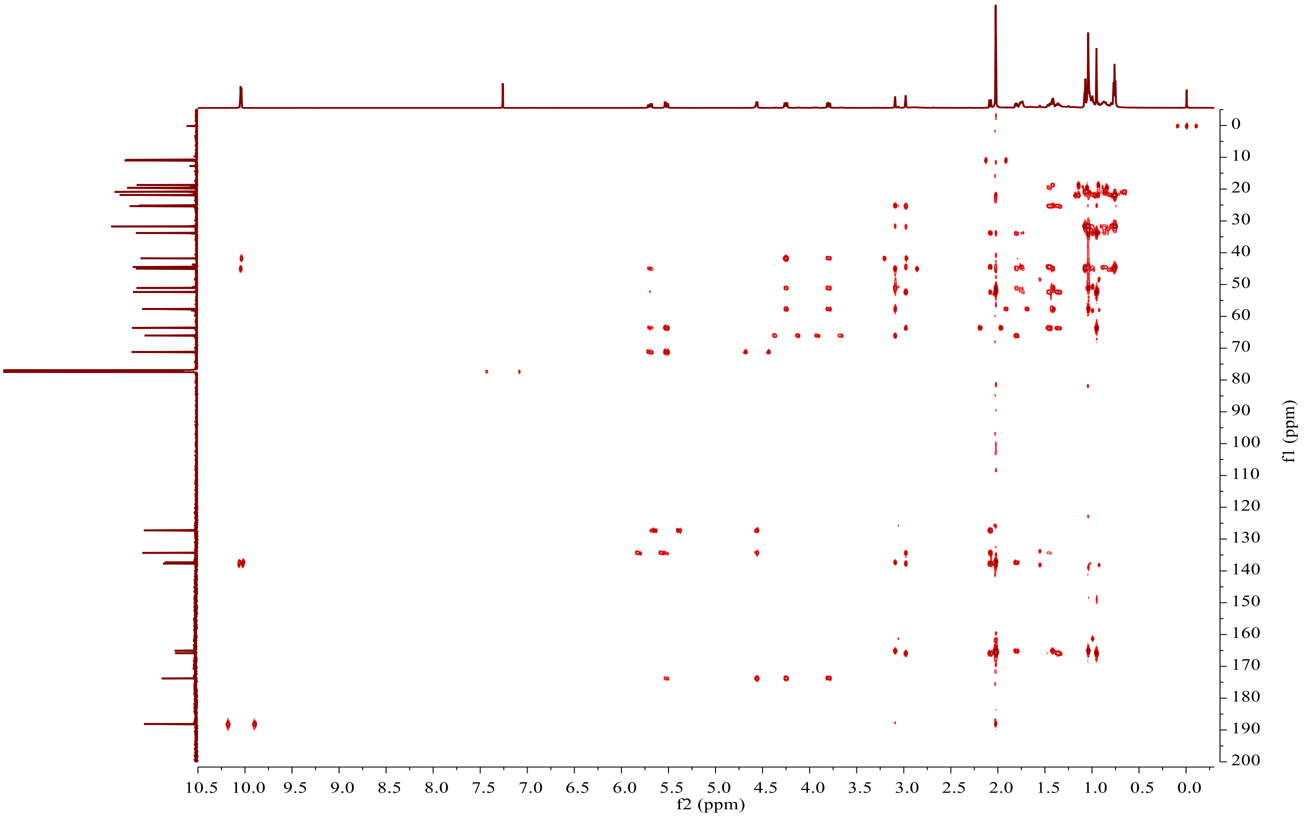

^{1}H-^{1}H COSY spectrum

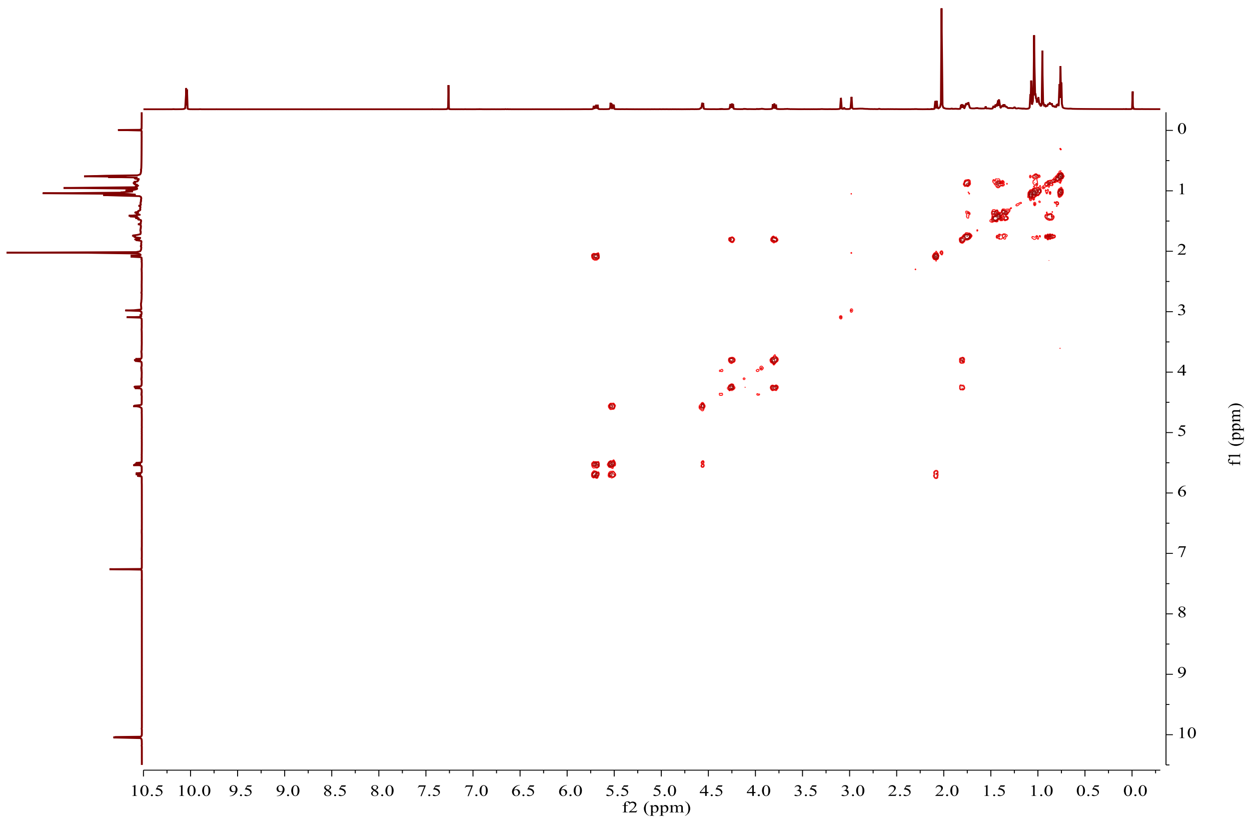

ROESY spectrum

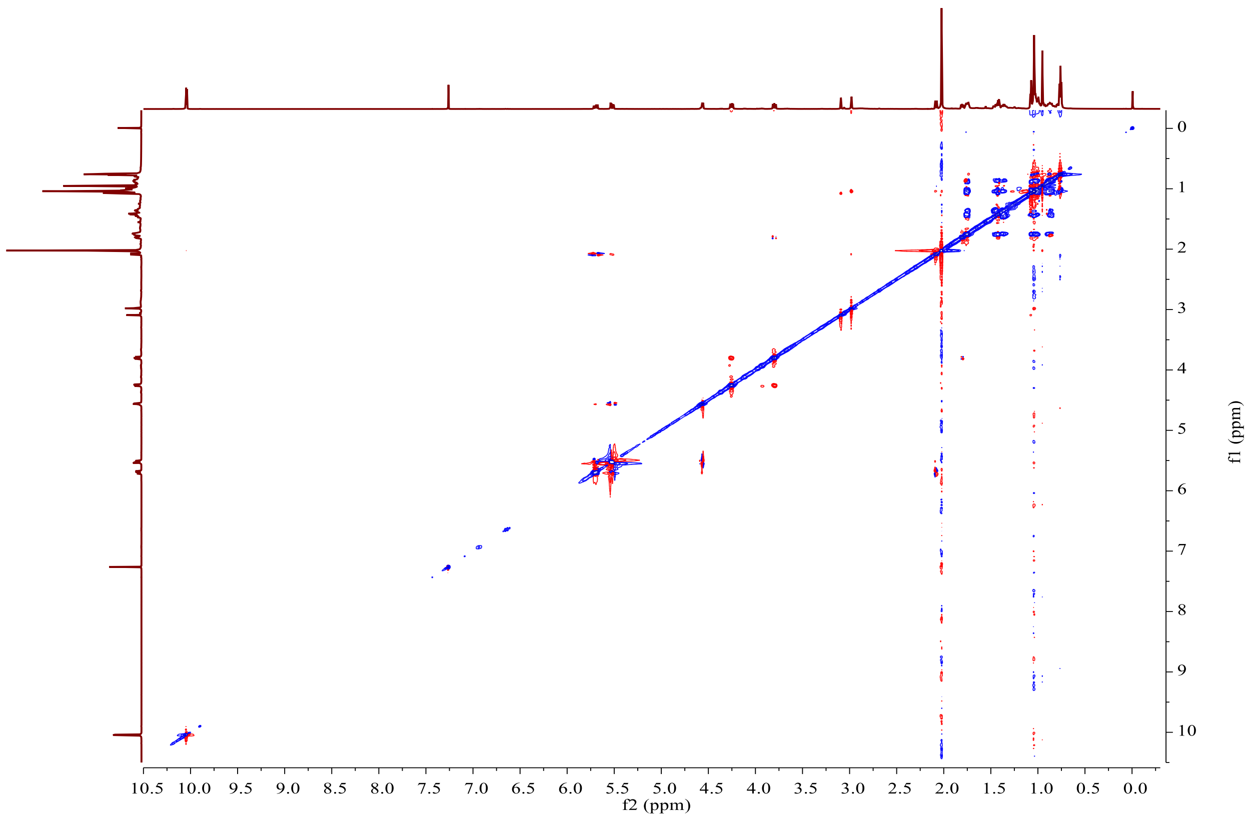

HRESIMS

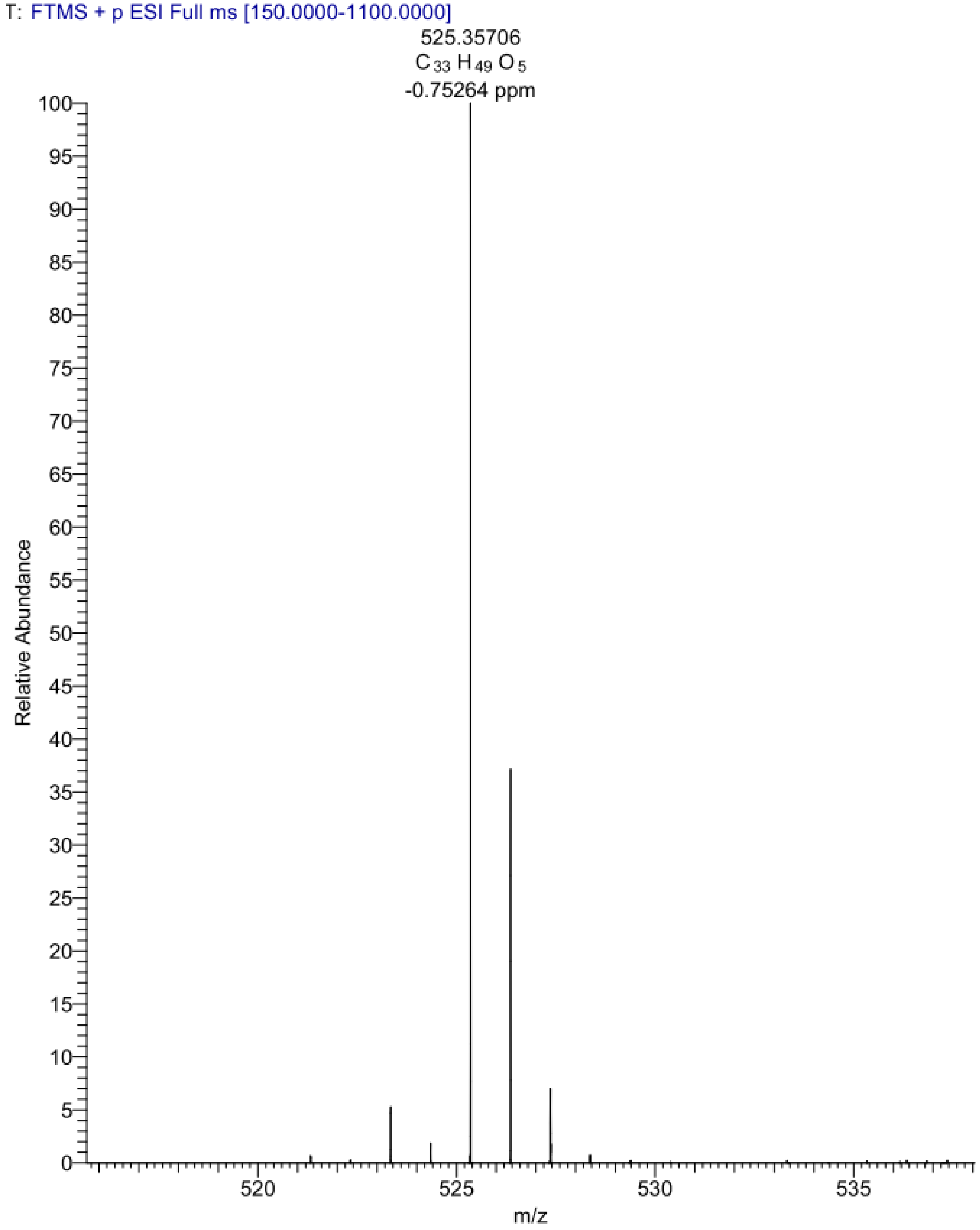
T: FTMS + p ESI Full ms [150.0000-1100.0000]
525.35706
C 33 H 49 O 5
-0.75264 ppm
Relative Abundance
m/z
100
95
90
85
80
75
70
65
60
55
50
45
40
35
30
25
20
15
10
5
0
520
525
530
535

CD spectrum

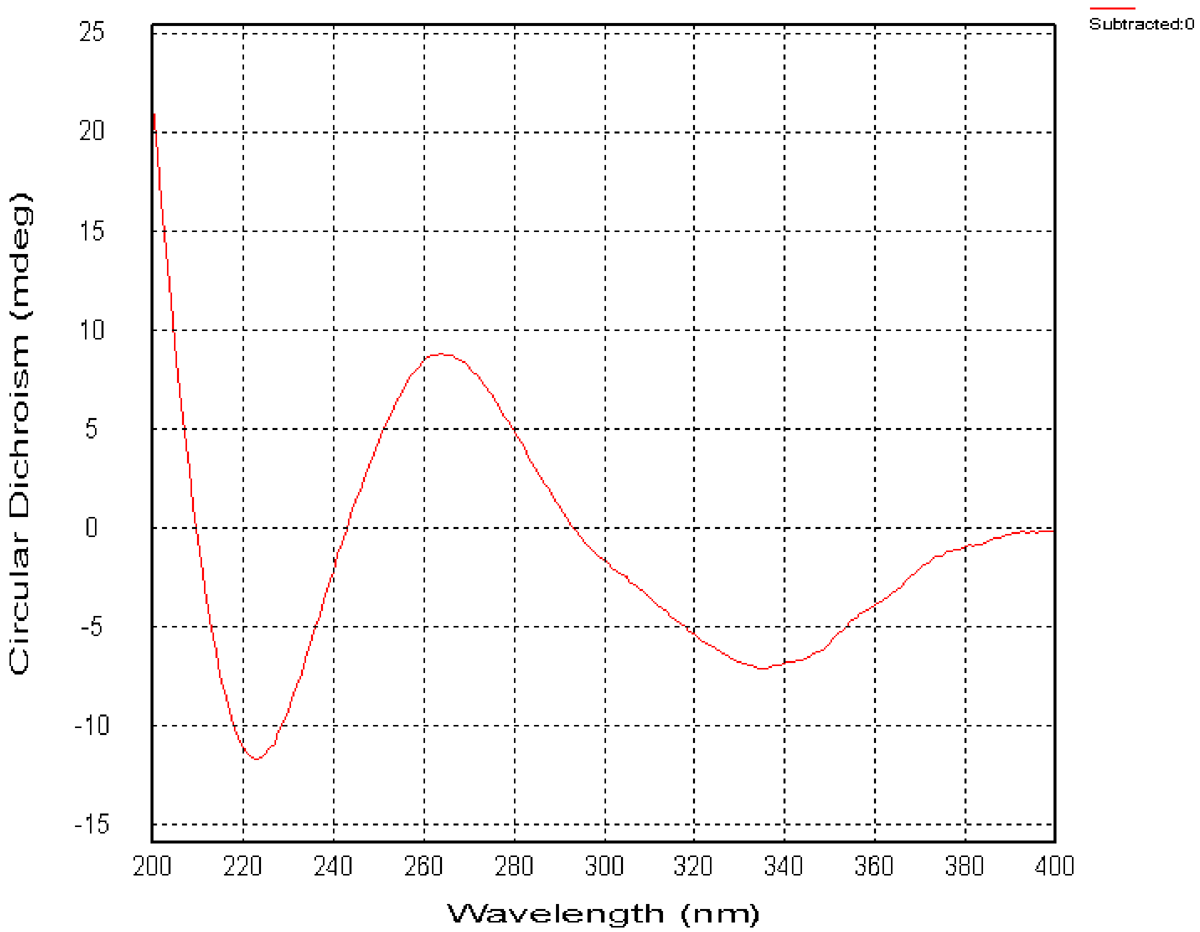

Subtracted:0
Circular Dichroism (mdeg)
25
20
15
10
5
0
-5
-10
-15
200
220
240
260
280
300
320
340
360
380
400
Wavelength (nm)

S1.6 NMR and HRESIMS spectra of bipolariterpene A

^{1}H NMR spectrum

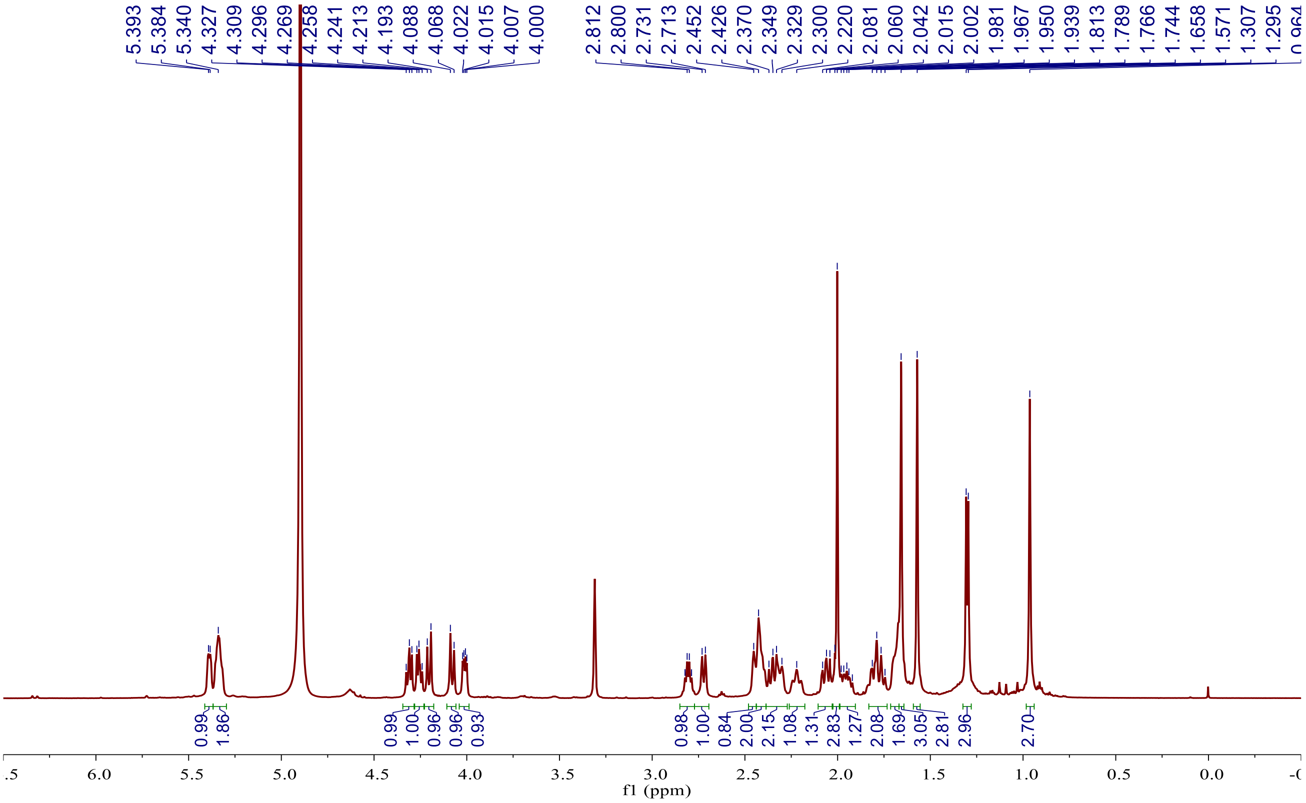

^{13}C NMR and DEPT spectra

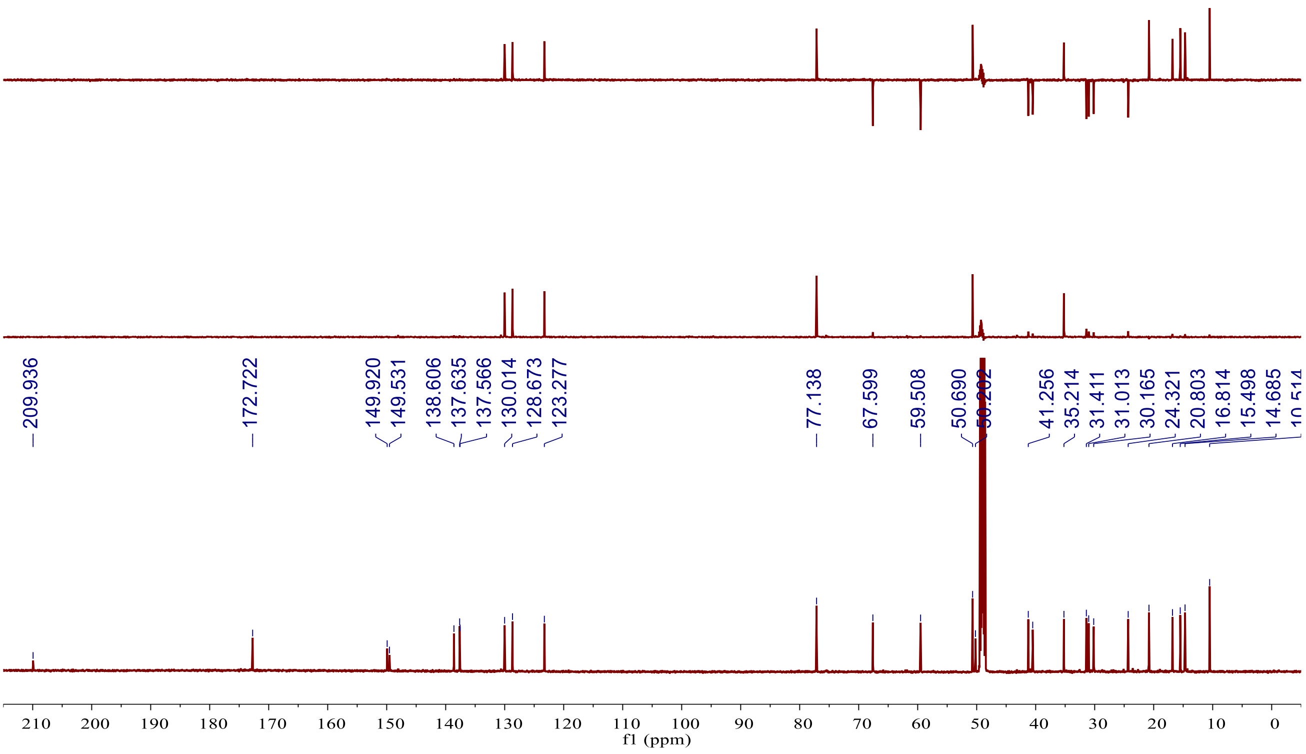

HSQC spectrum

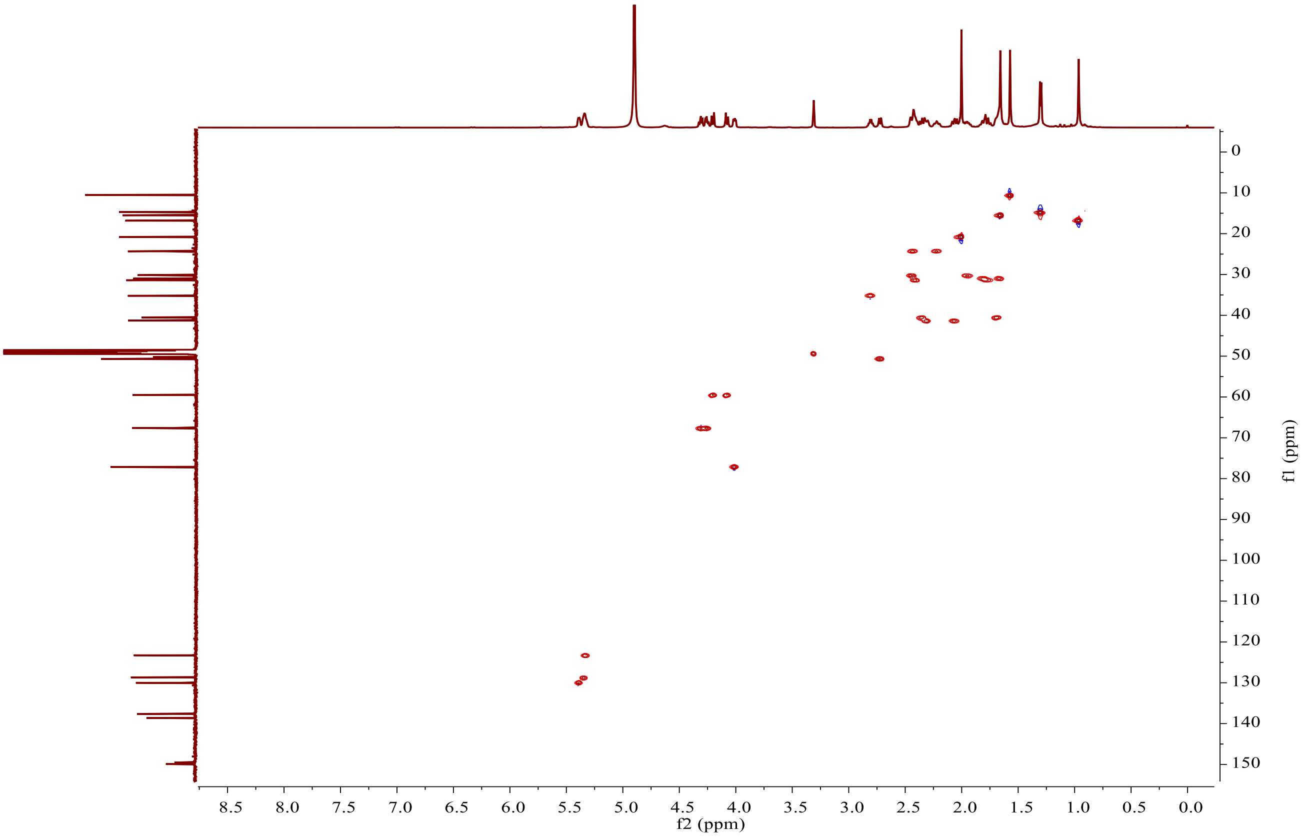

HMBC spectrum

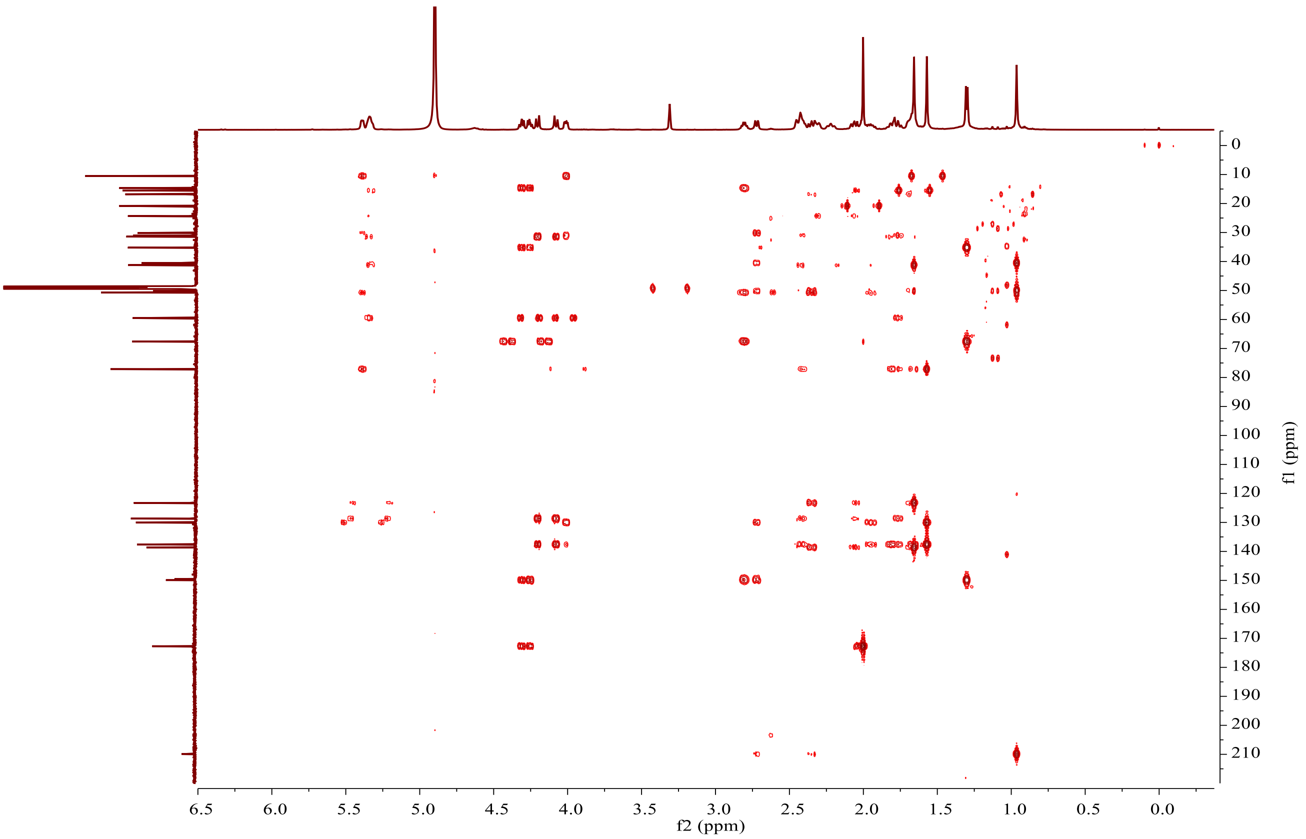

^1H-^1H COSY spectrum

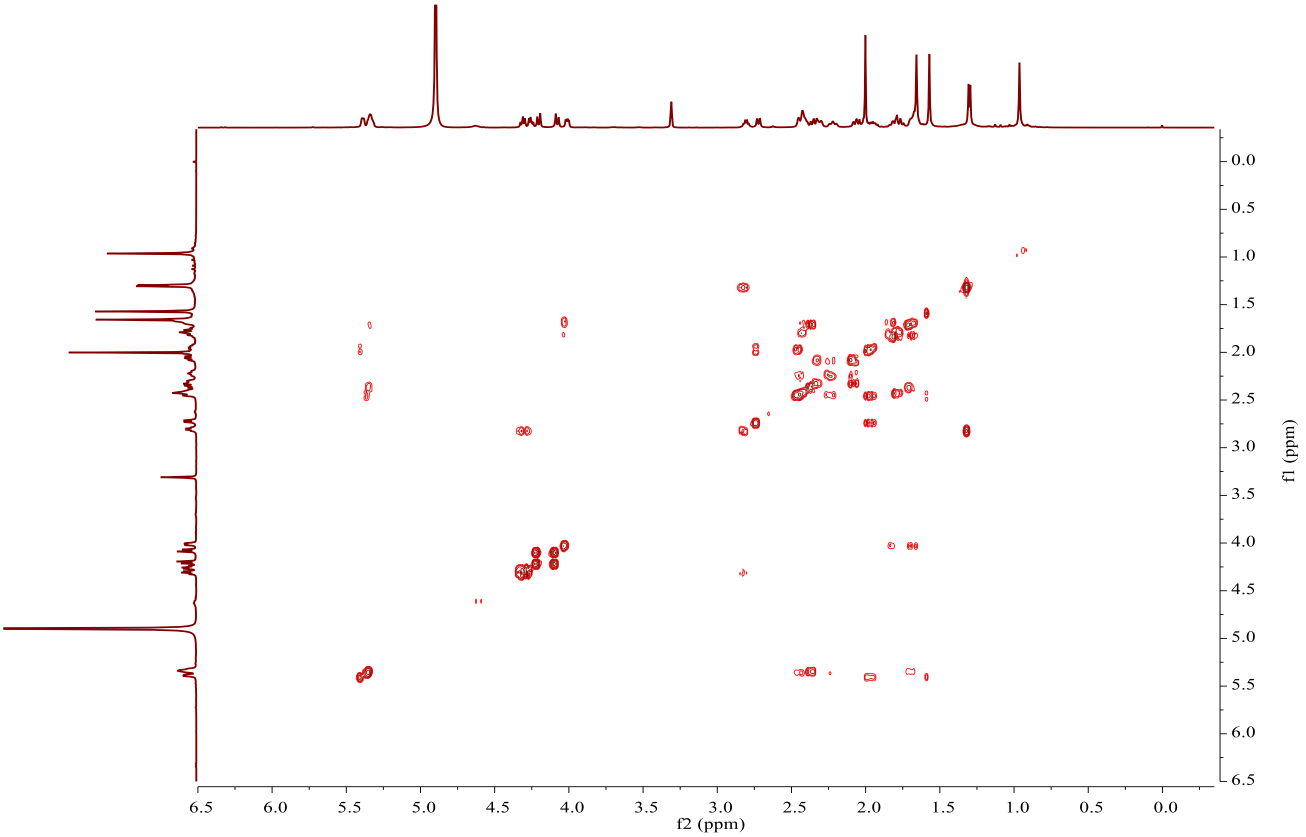

ROESY spectrum

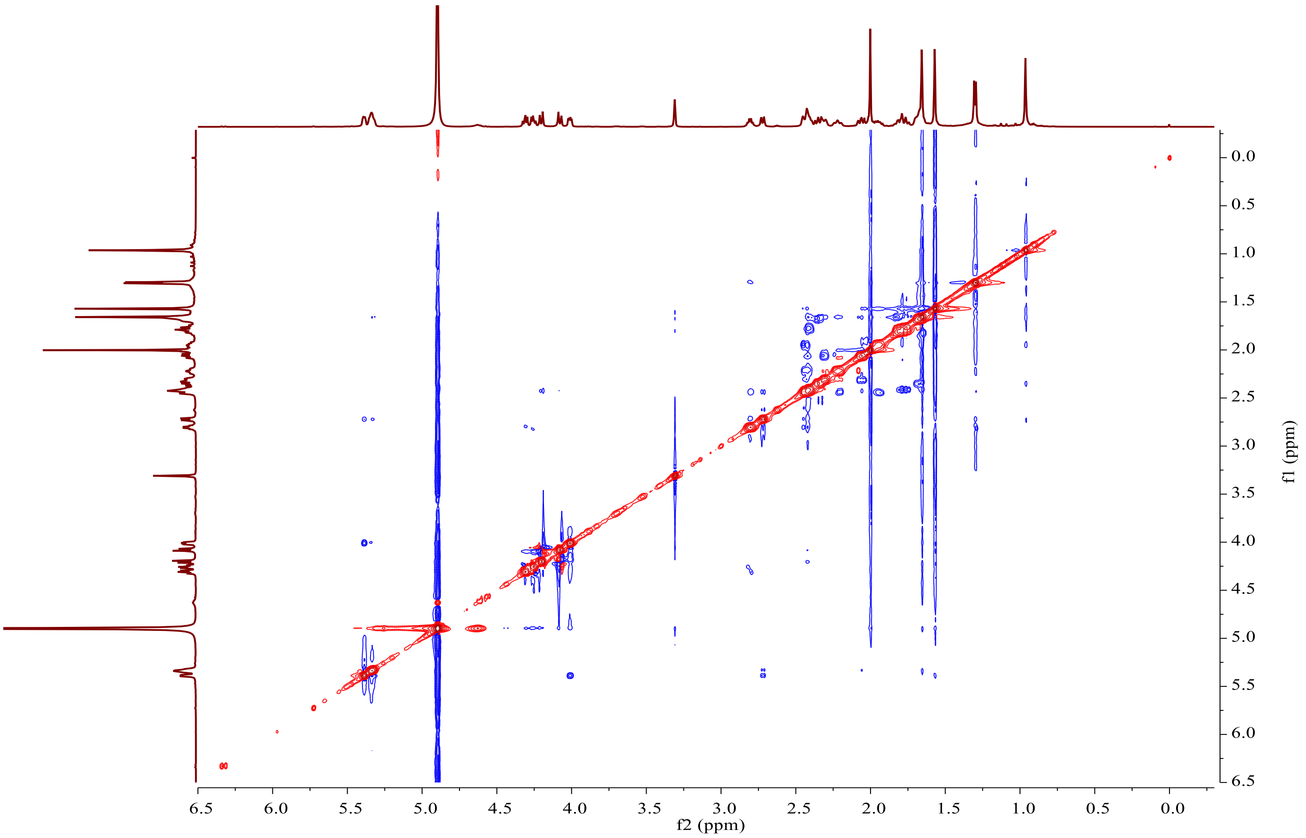

HRESIMS

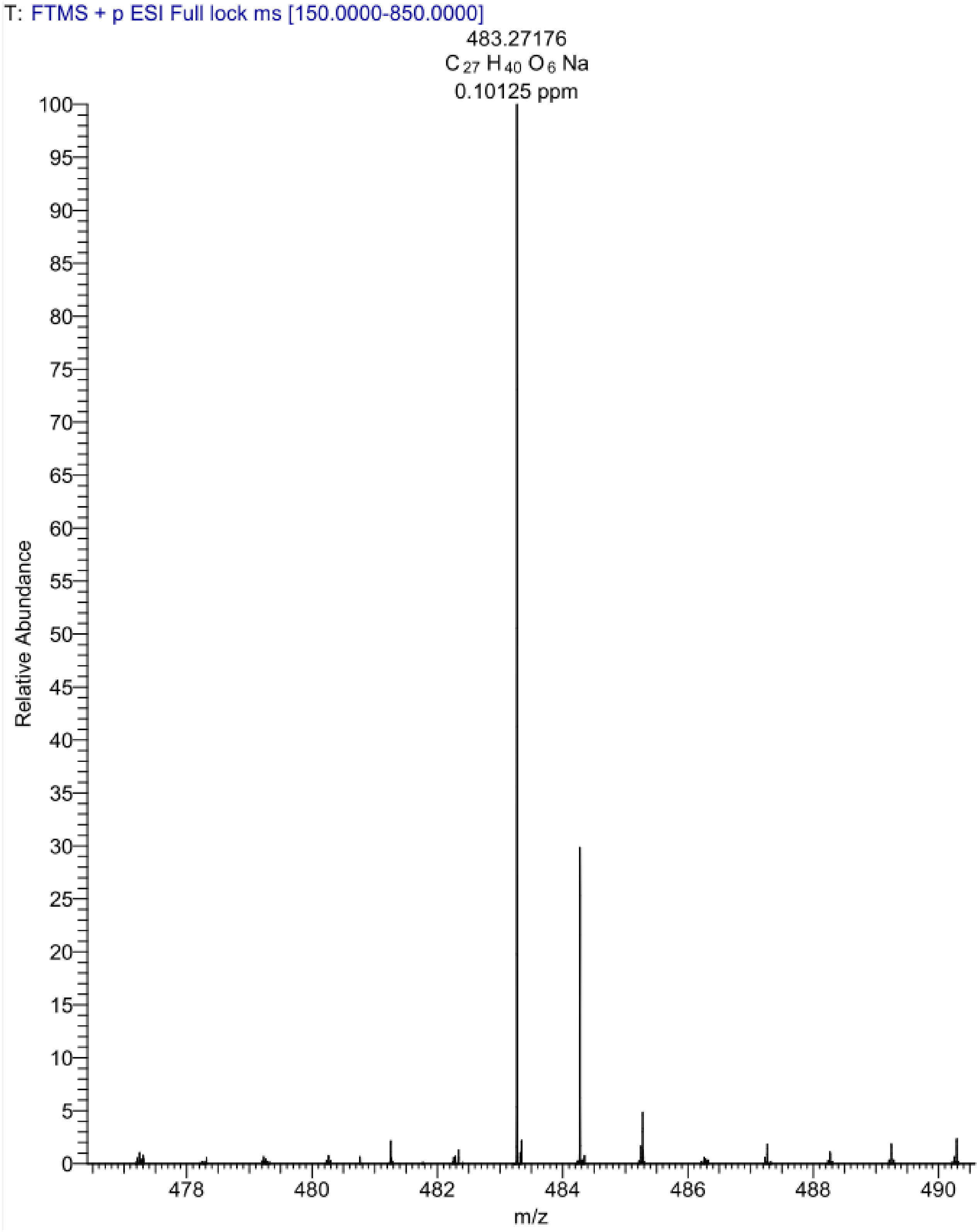
T: FTMS + p ESI Full lock ms [150.0000-850.0000]
483.27176
C 27 H 40 O 6 Na
0.10125 ppm
Relative Abundance
m/z
0
5
10
15
20
25
30
35
40
45
50
55
60
65
70
75
80
85
90
95
100
478
480
482
484
486
488
490

S1.7 NMR and HRESIMS spectra of bipolariterpene B

^{1}H NMR spectrum

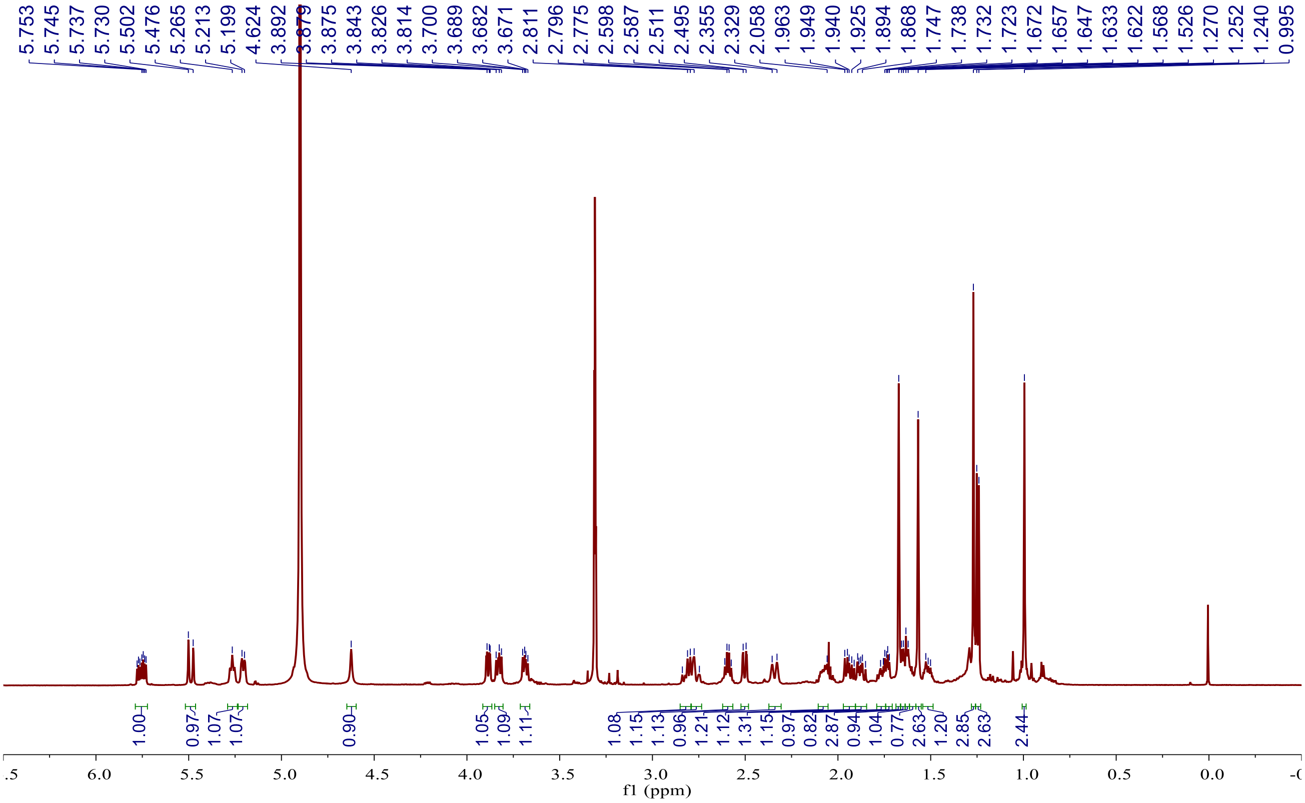

^{13}C NMR and DEPT spectra

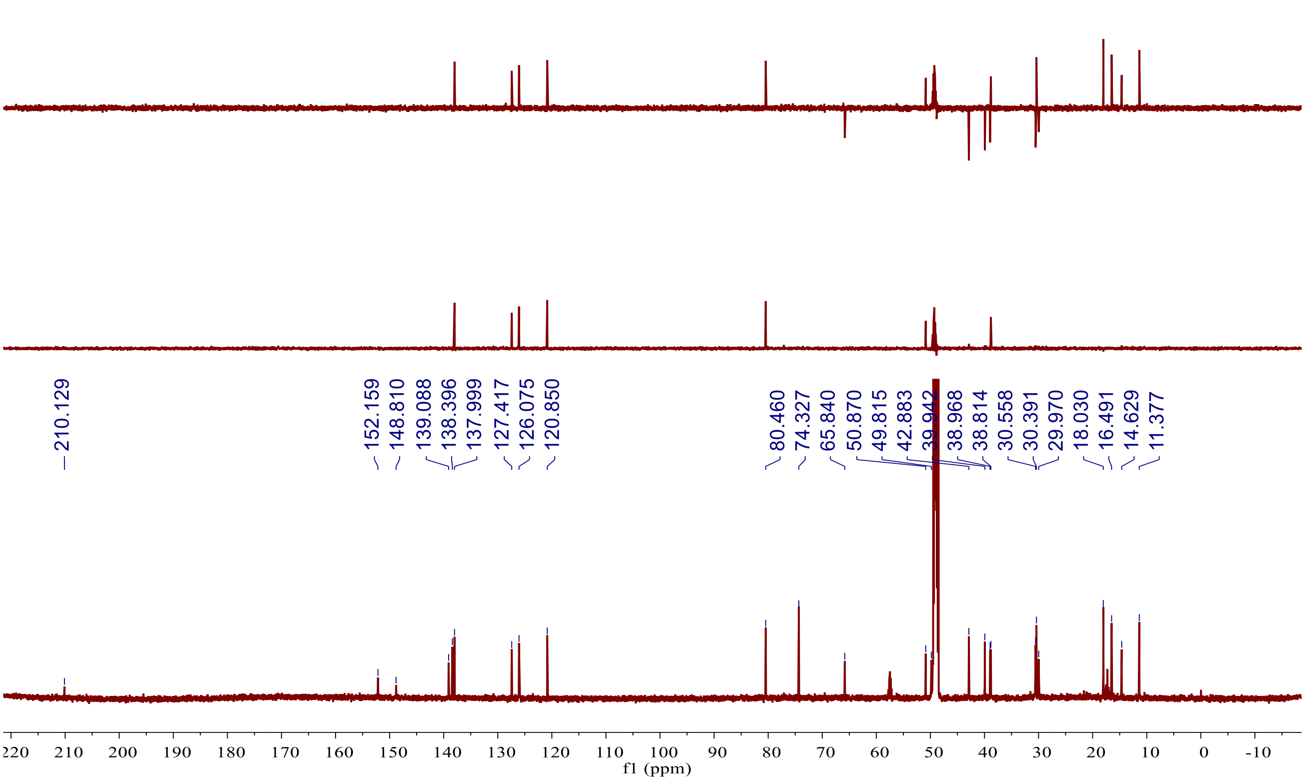

HSQC spectrum

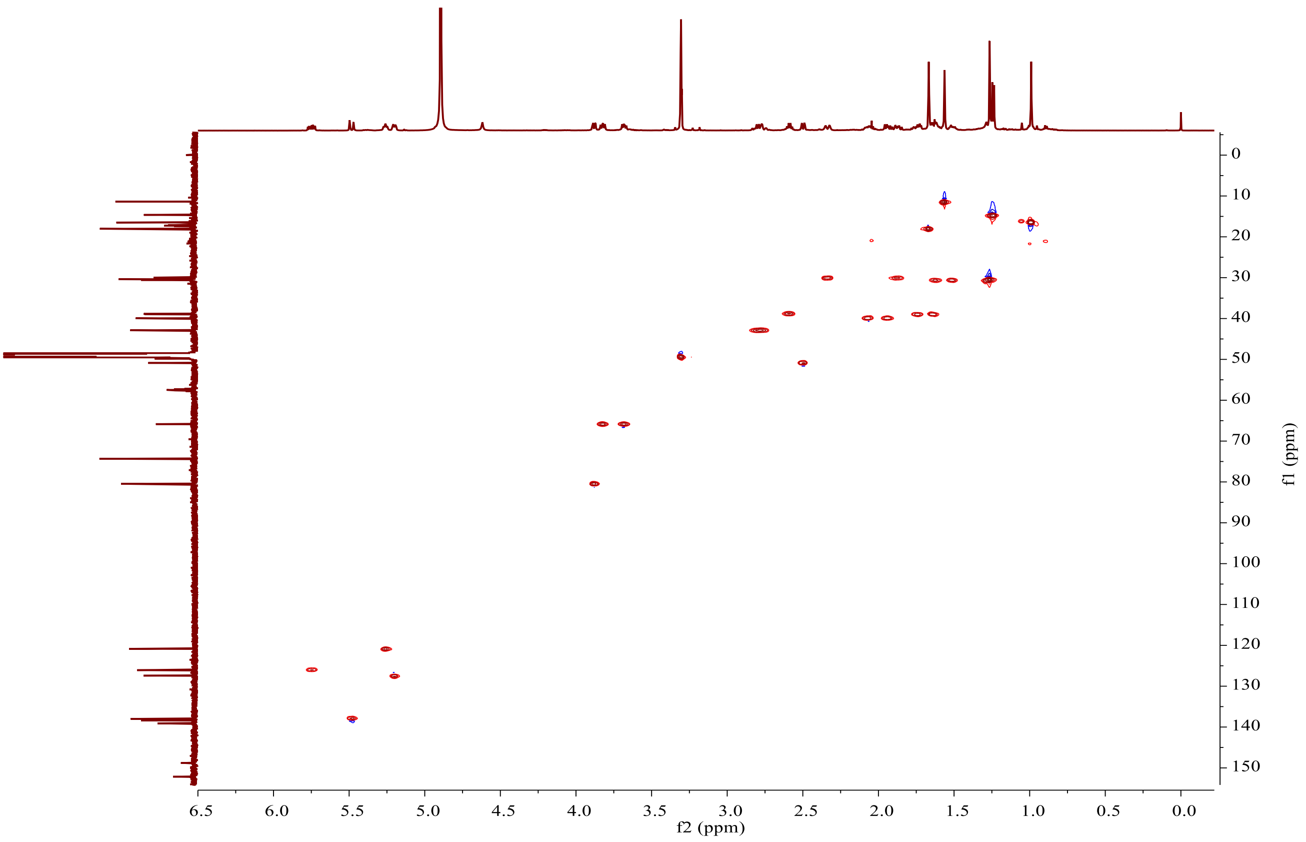

HMBC spectrum

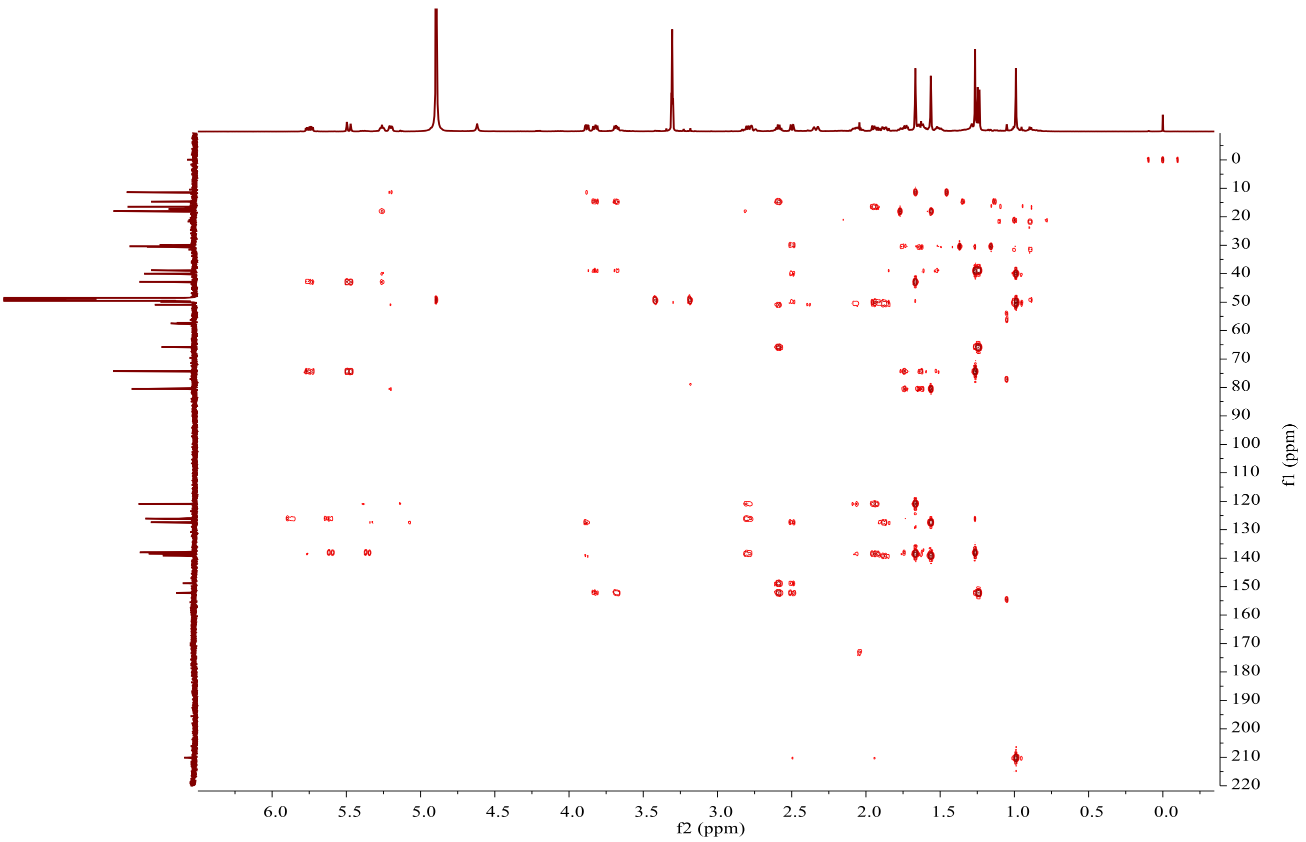

^{1}H-^{1}H COSY spectrum

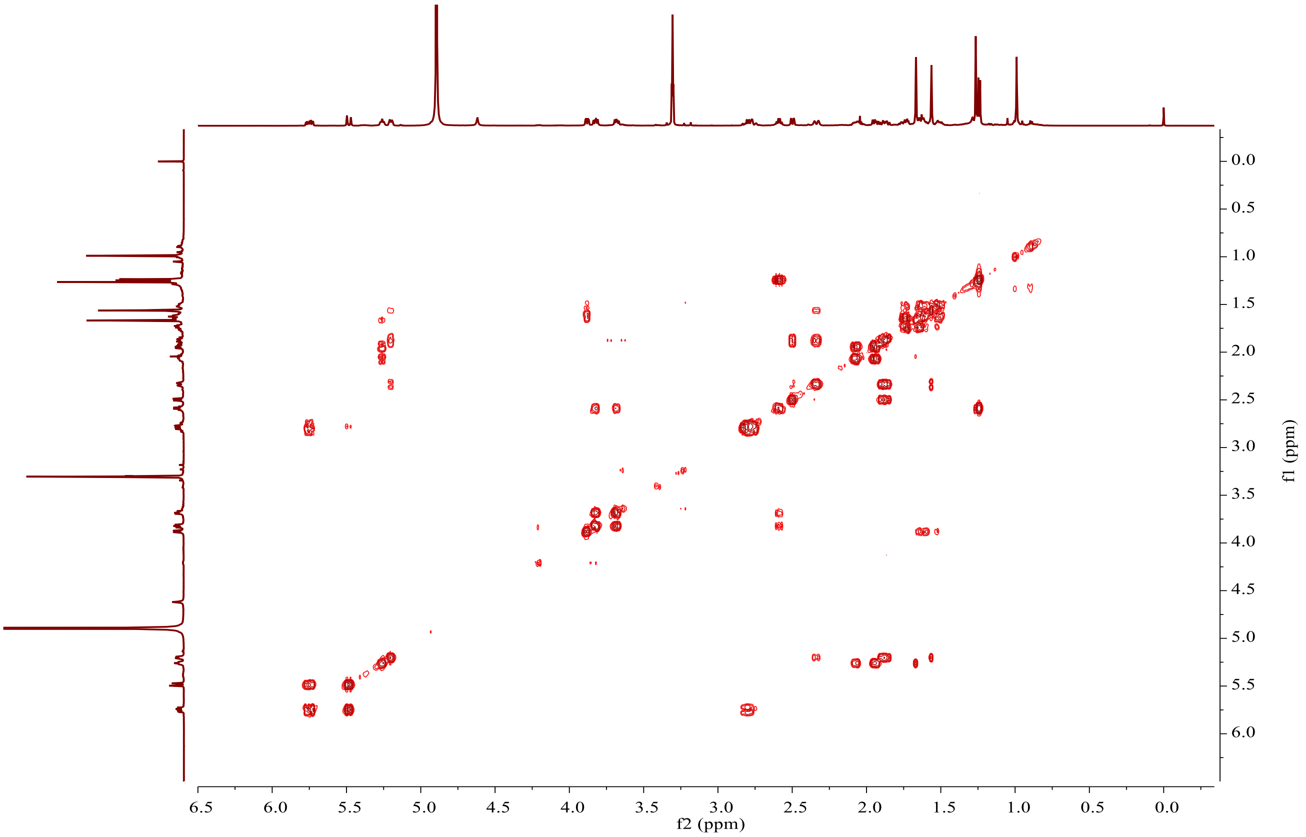

ROESY spectrum

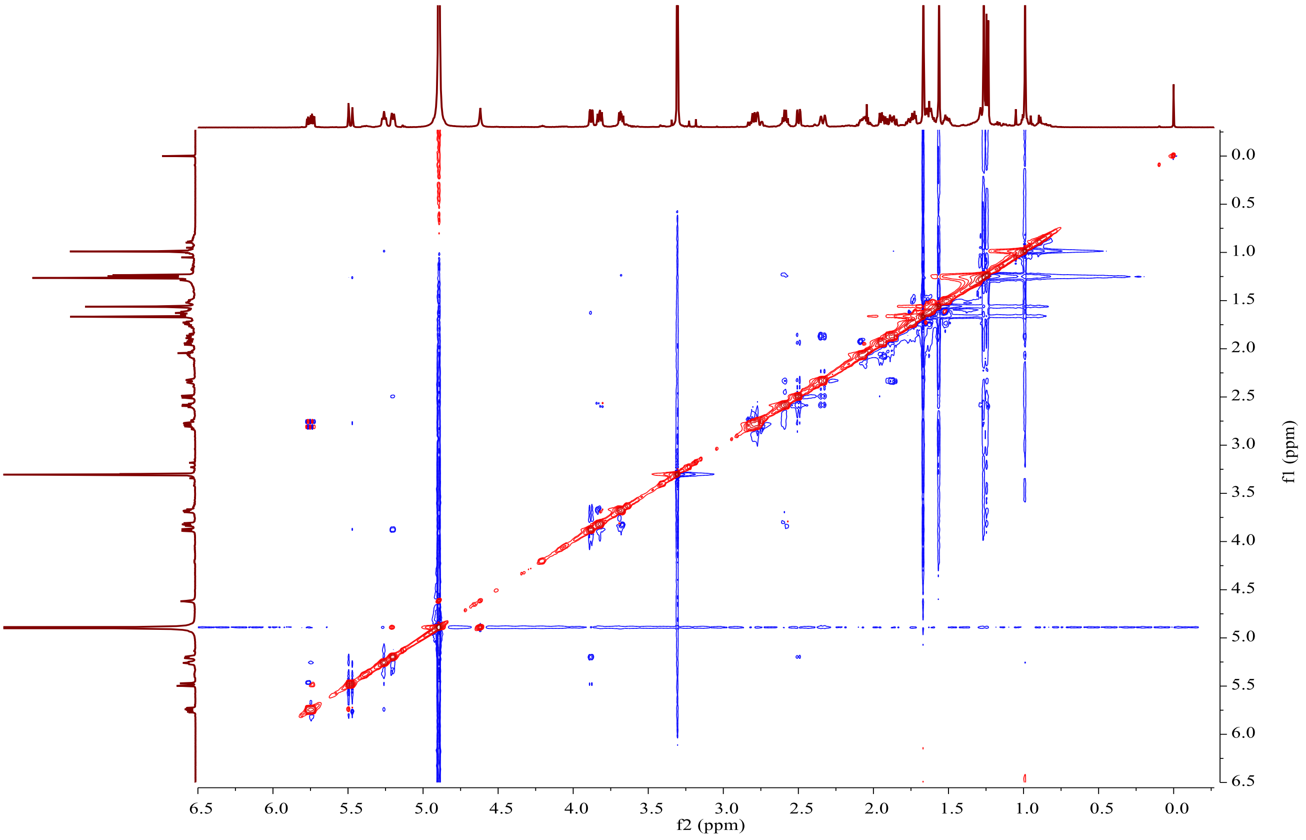

HRESIMS

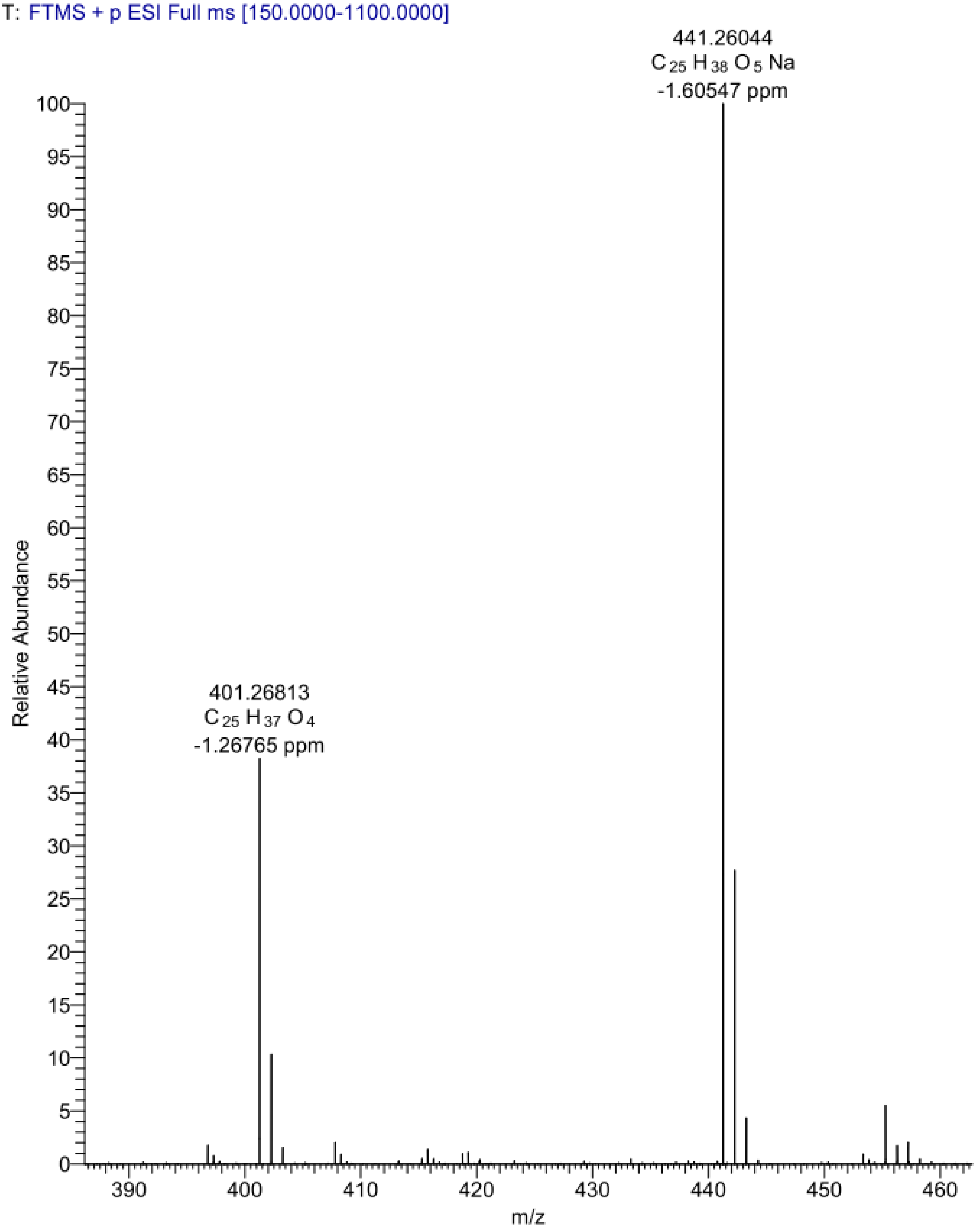

T: FTMS + p ESI Full ms [150.0000-1100.0000]
441.26044
C 25 H 38 O 5 Na
-1.60547 ppm
401.26813
C 25 H 37 O 4
-1.26765 ppm
Relative Abundance
m/z

S1.8 NMR and HRESIMS spectra of bipolariterpene C

^{1}H NMR spectrum

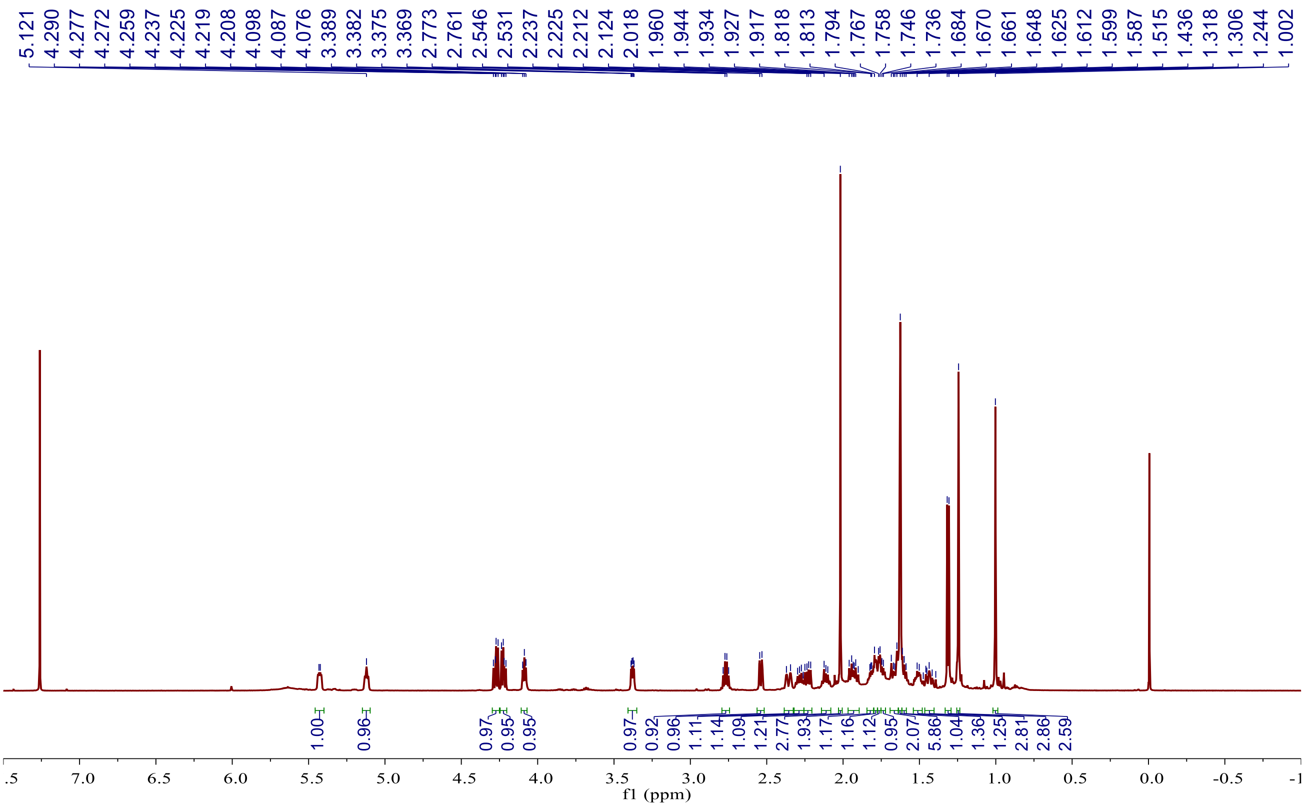

^{13}C NMR and DEPT spectra

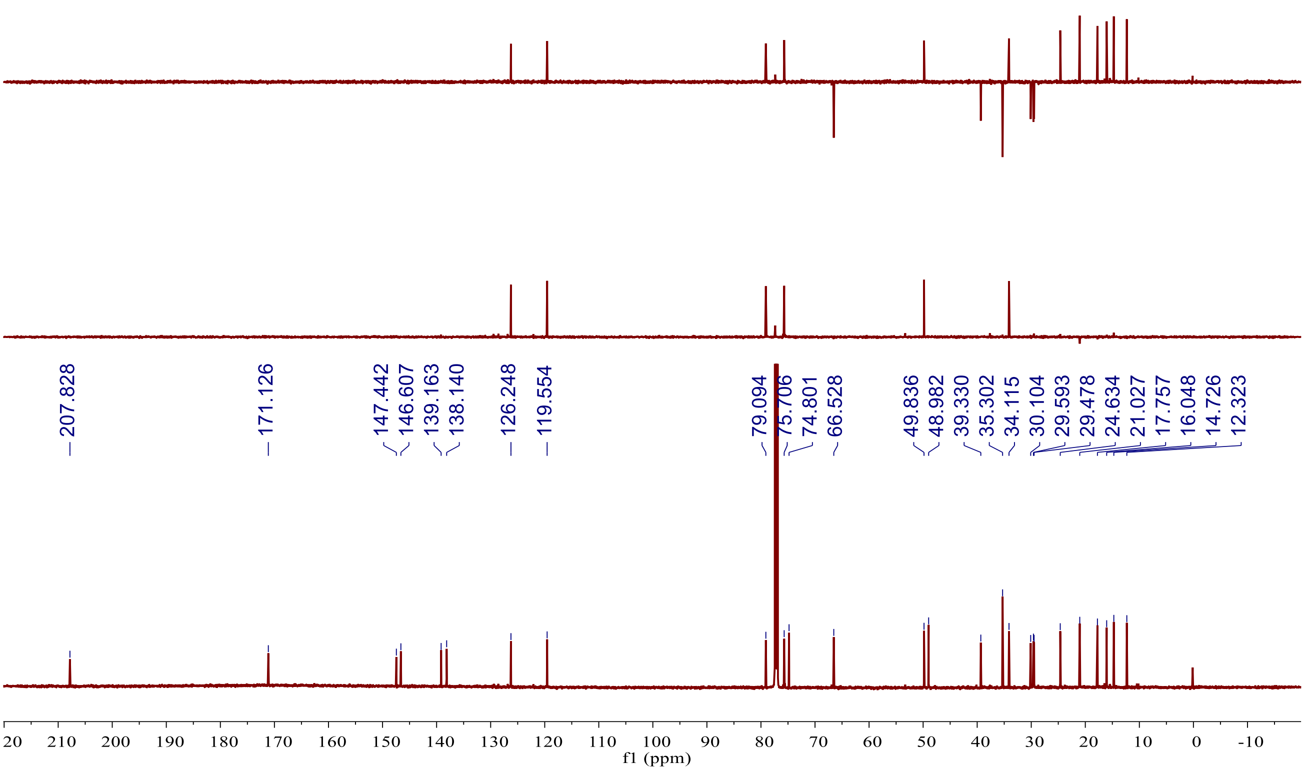

HSQC spectrum

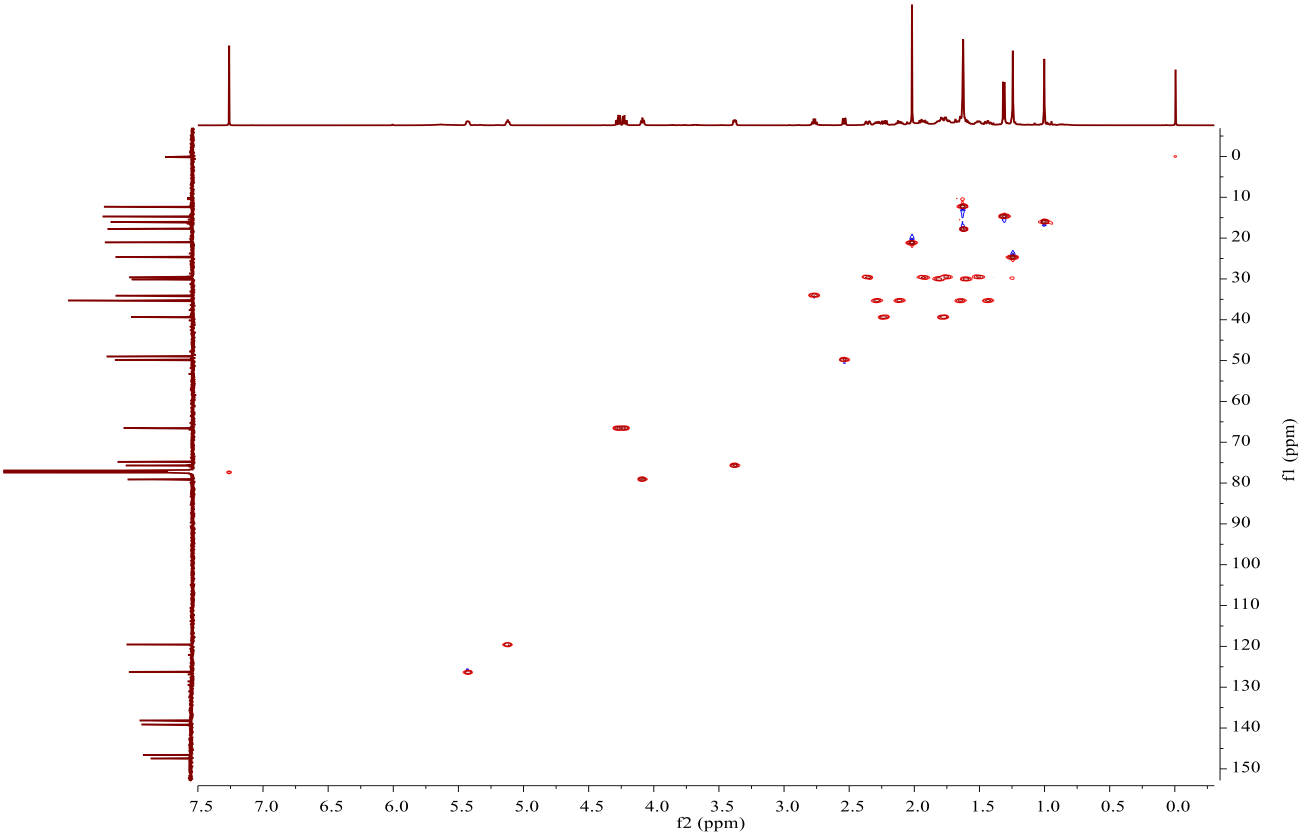

HMBC spectrum

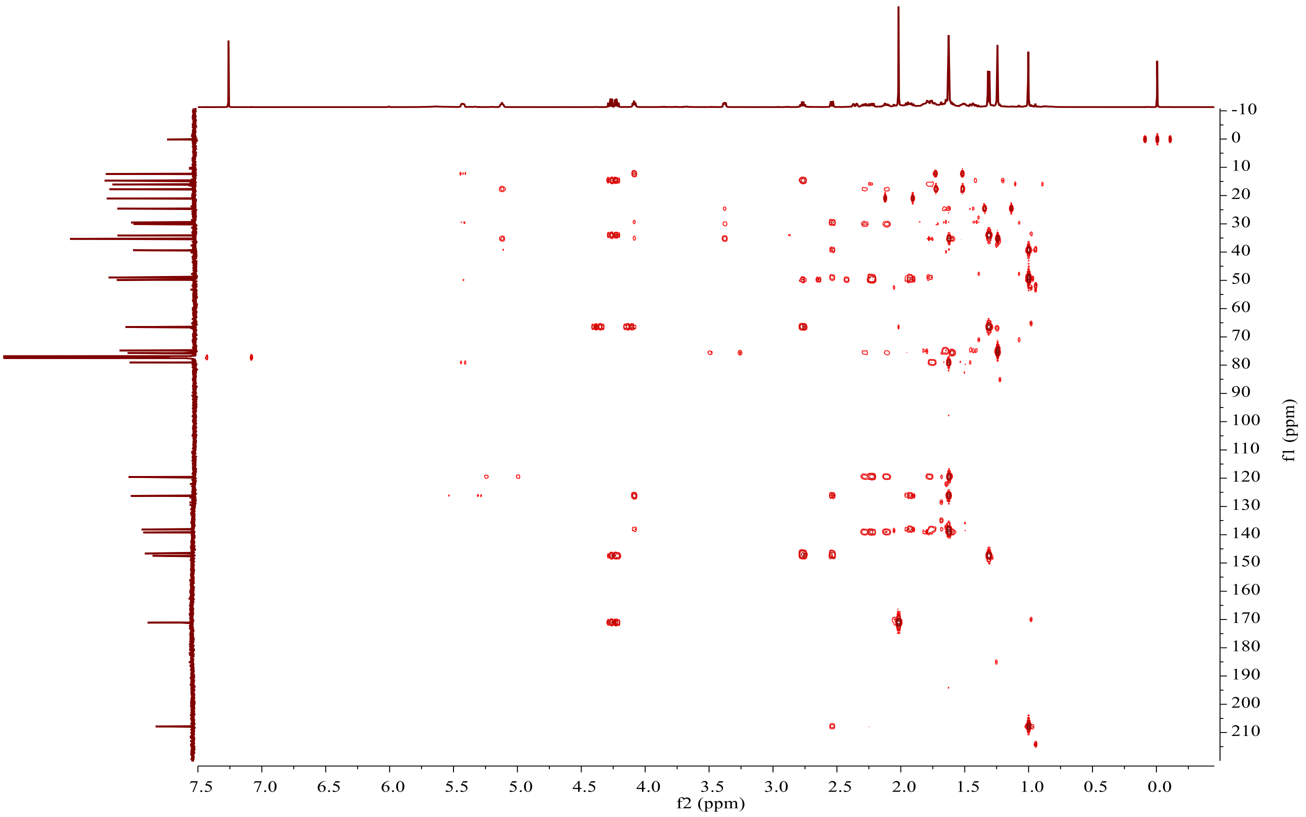

^1H-^1H COSY spectrum

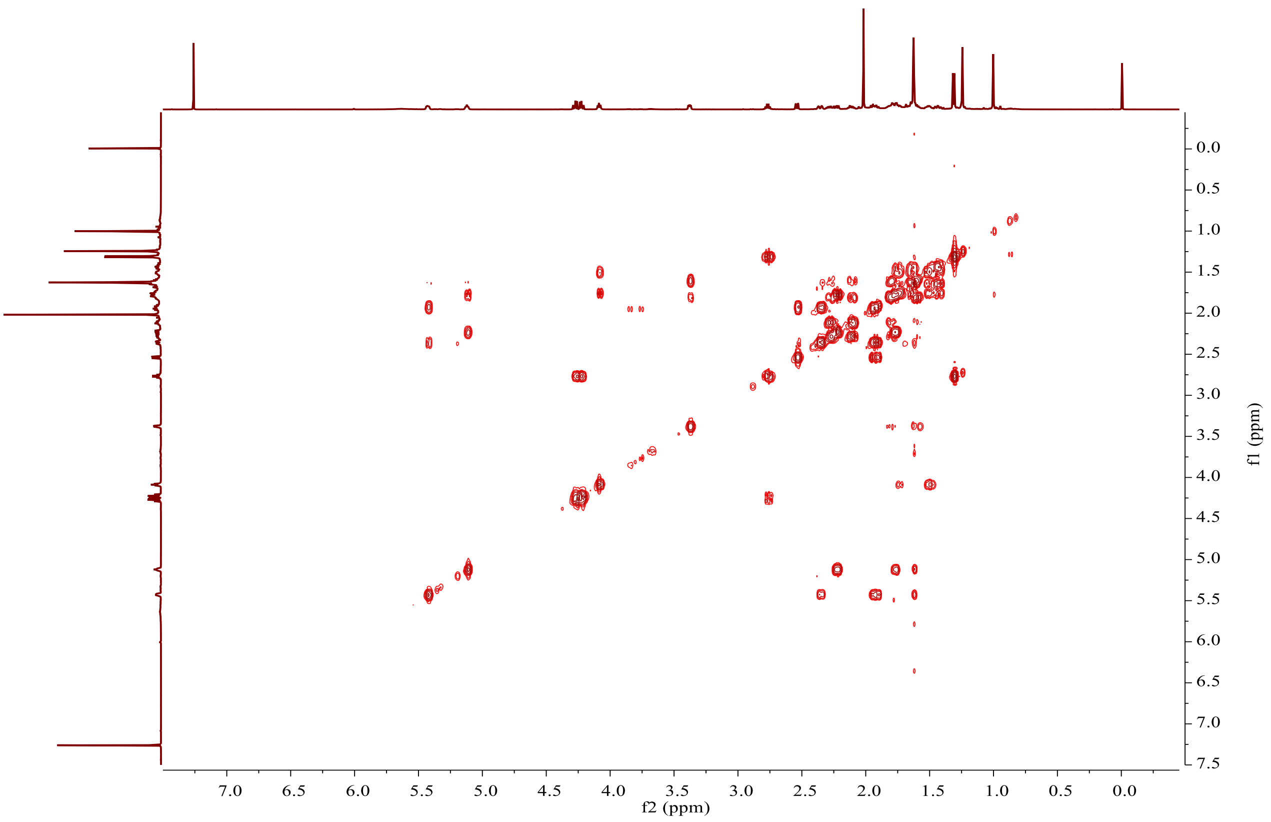

ROESY spectrum

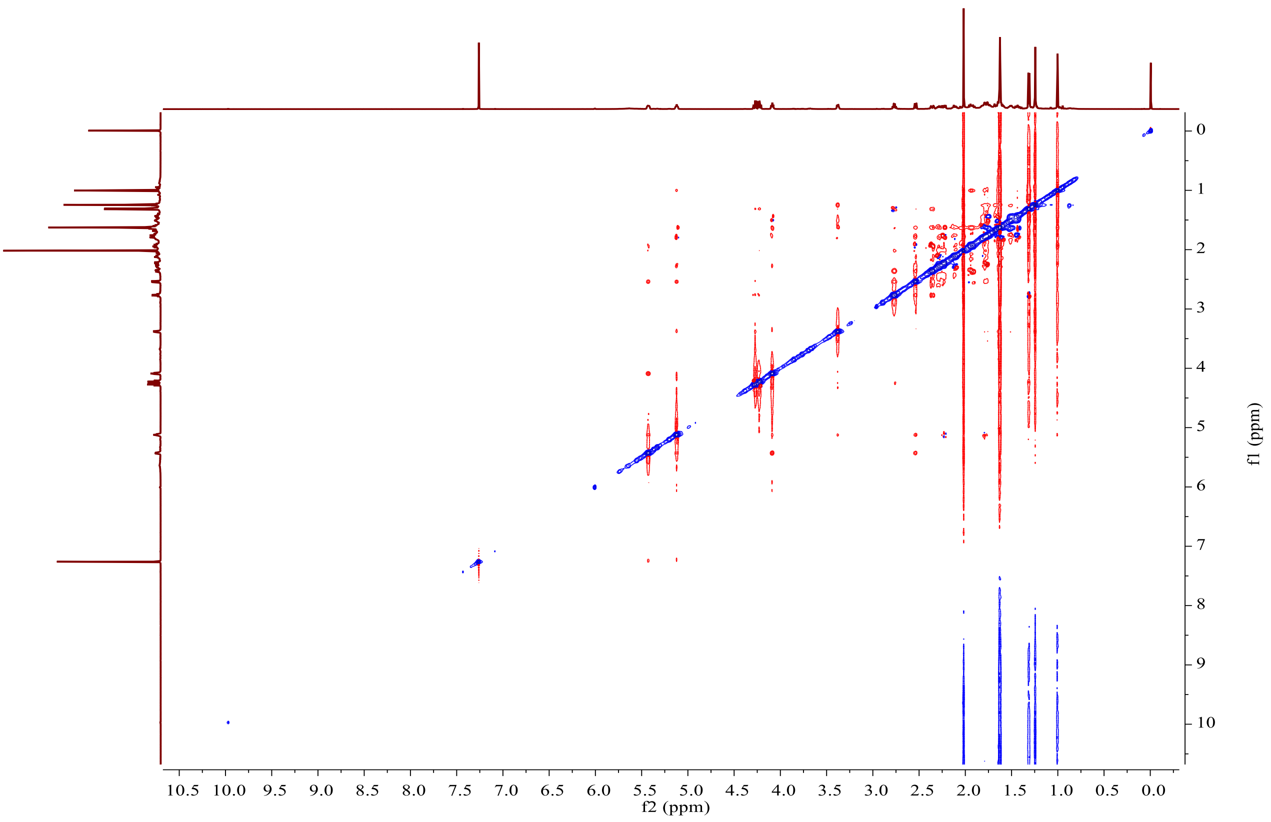

HRESIMS

T: FTMS + p ESI Full ms [150.0000-1100.0000]

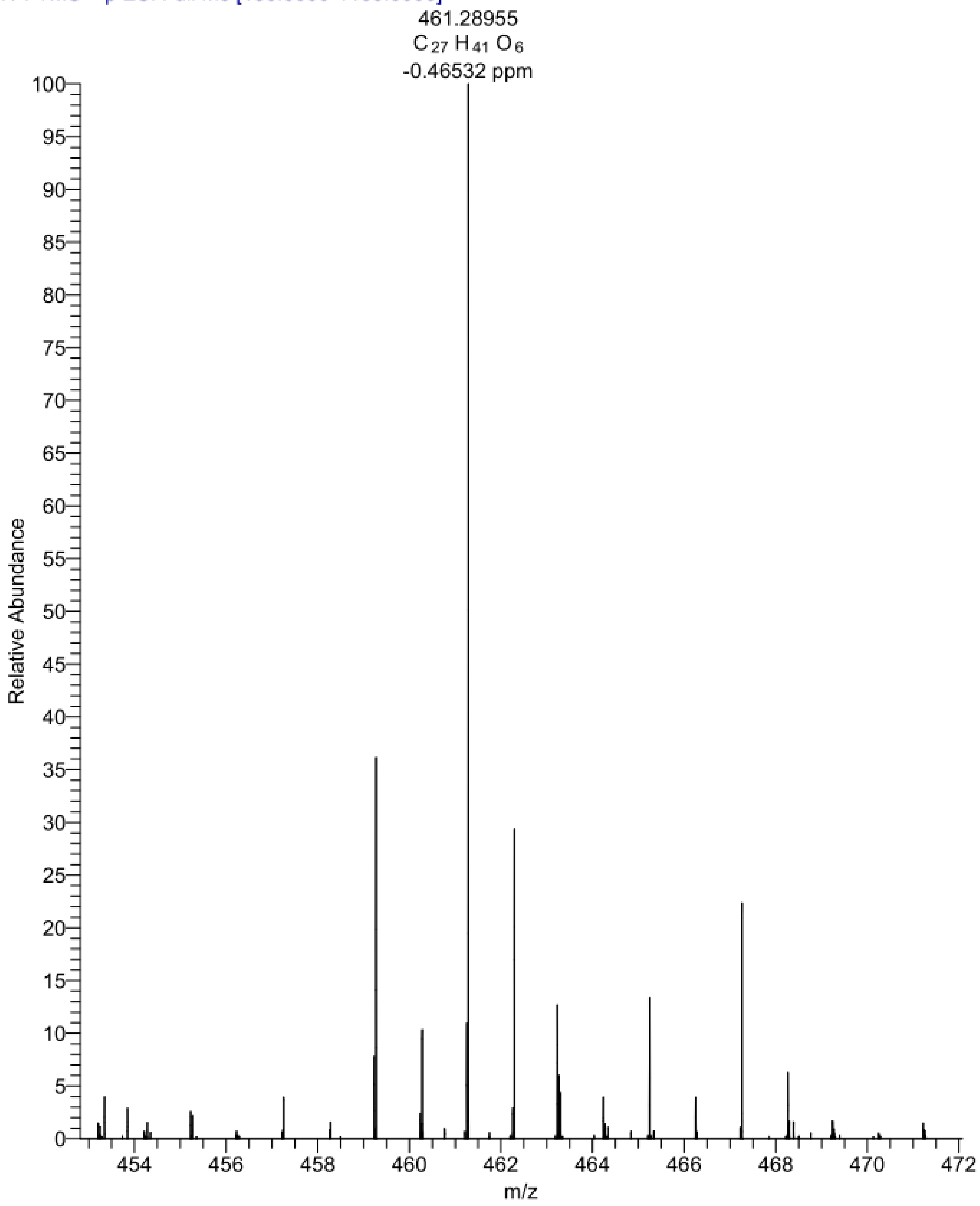

S1.9 ¹H NMR, ¹H−¹H COSY and HRESIMS spectra of (*S*)-MTPA ester (1a)

¹H NMR spectrum

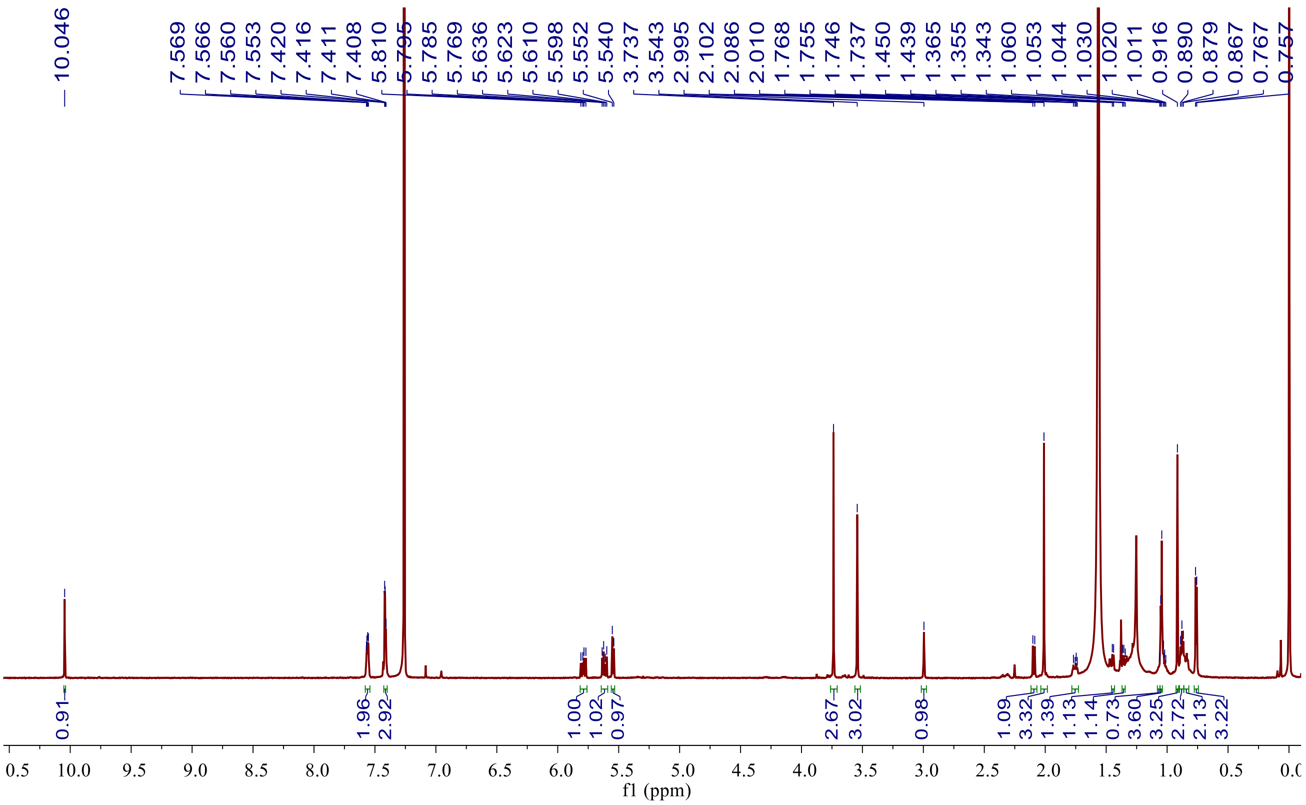

¹H-¹H COSY spectrum

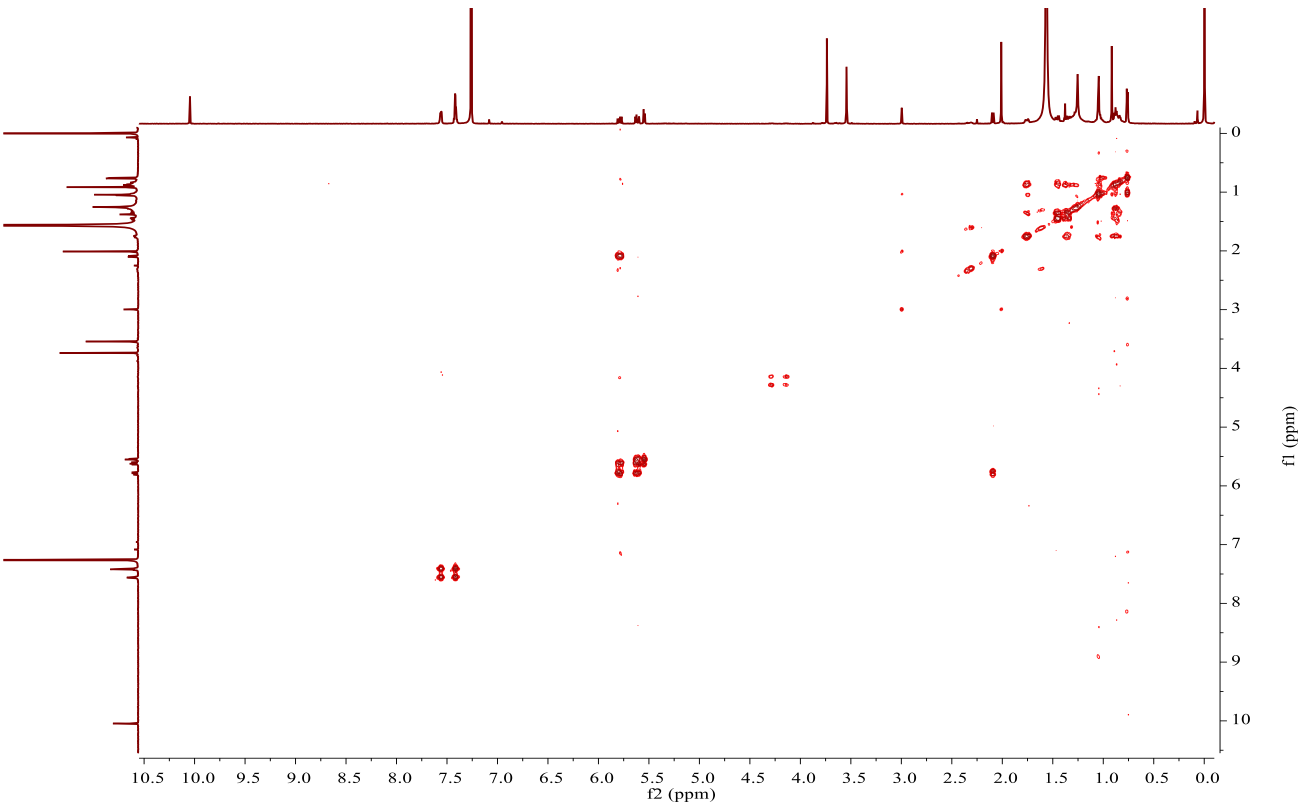

HRESIMS

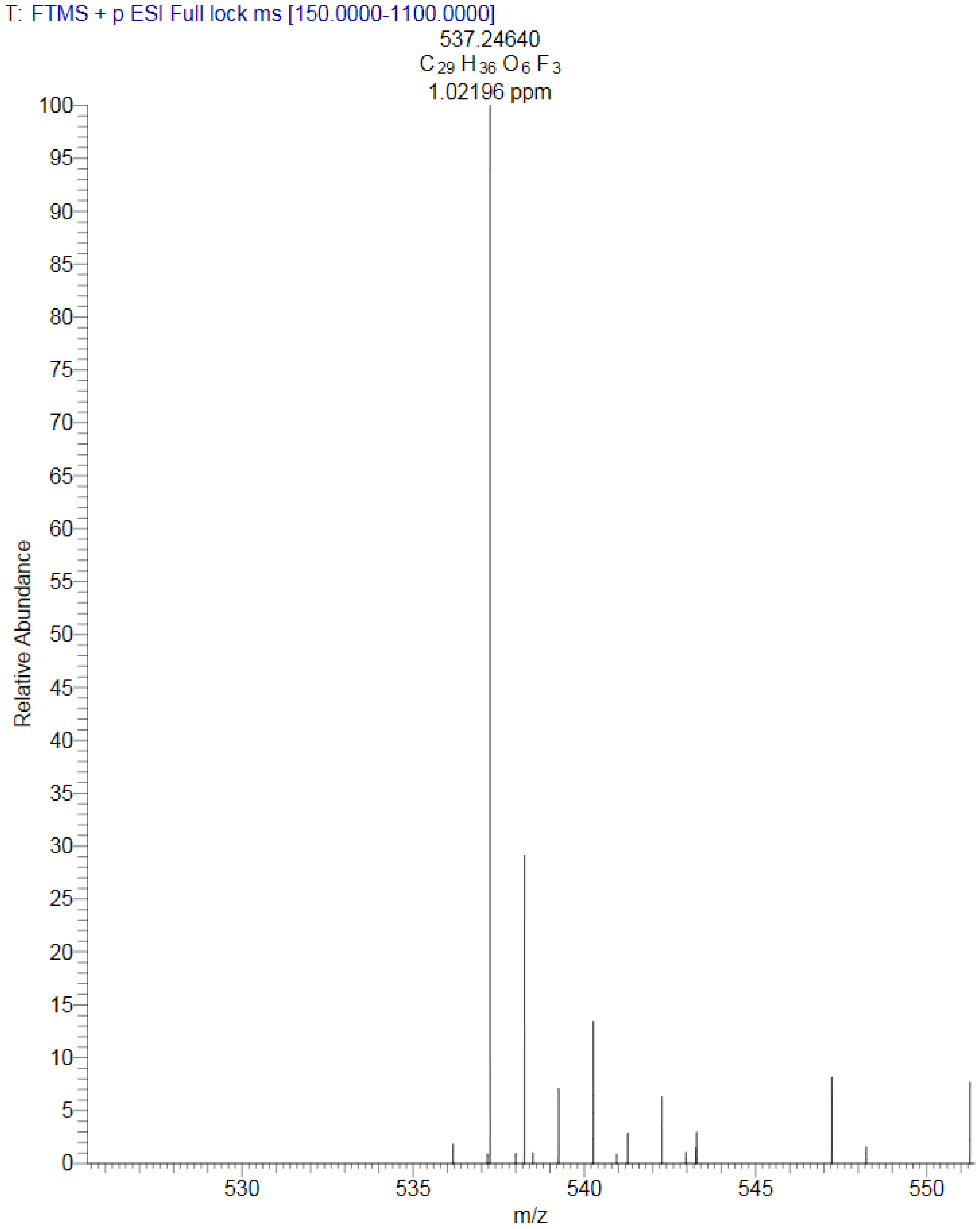

T: FTMS + p ESI Full lock ms [150.0000-1100.0000]
537.24640
C 29 H 36 O 6 F 3
1.02196 ppm
Relative Abundance
m/z

S1.10 ¹H NMR, ¹H−¹H COSY and HRESIMS spectra of (*R*)-MTPA ester (1b)

¹H NMR spectrum

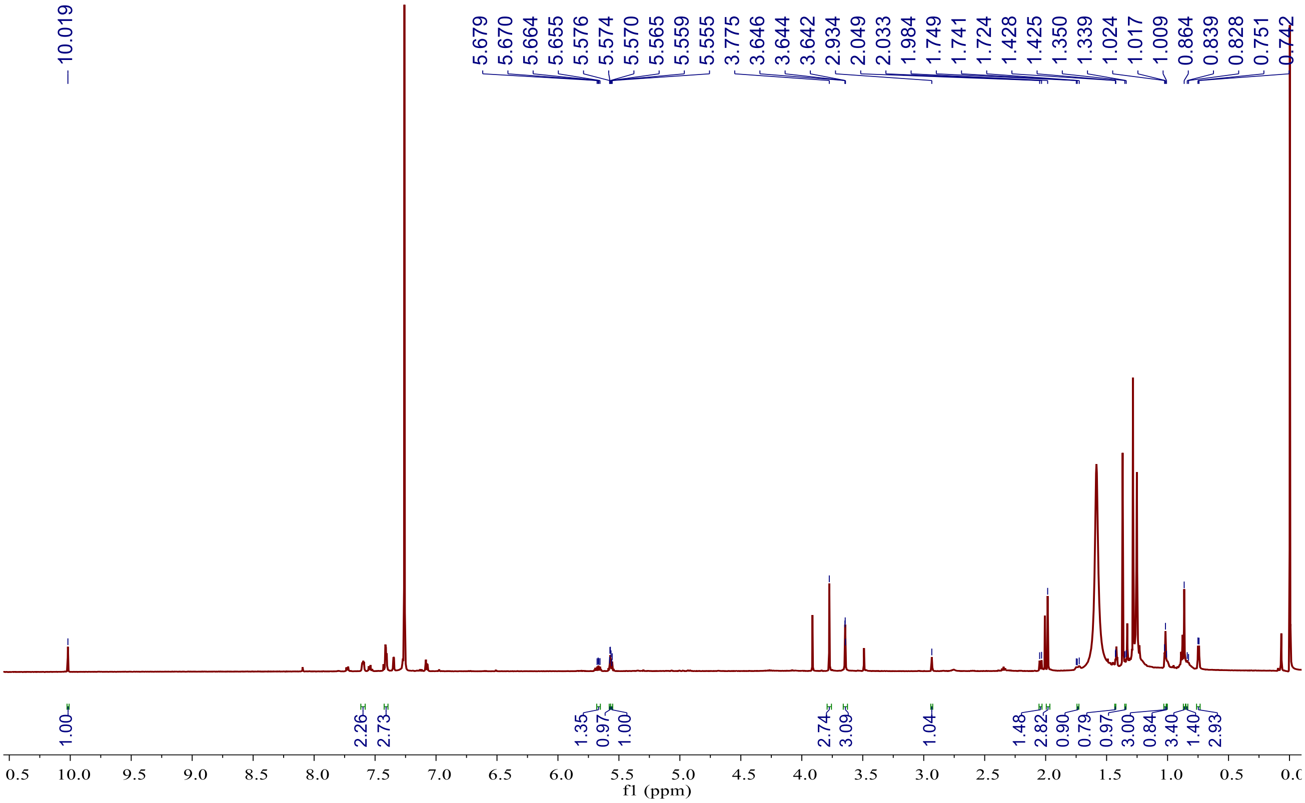

¹H-¹H COSY spectrum

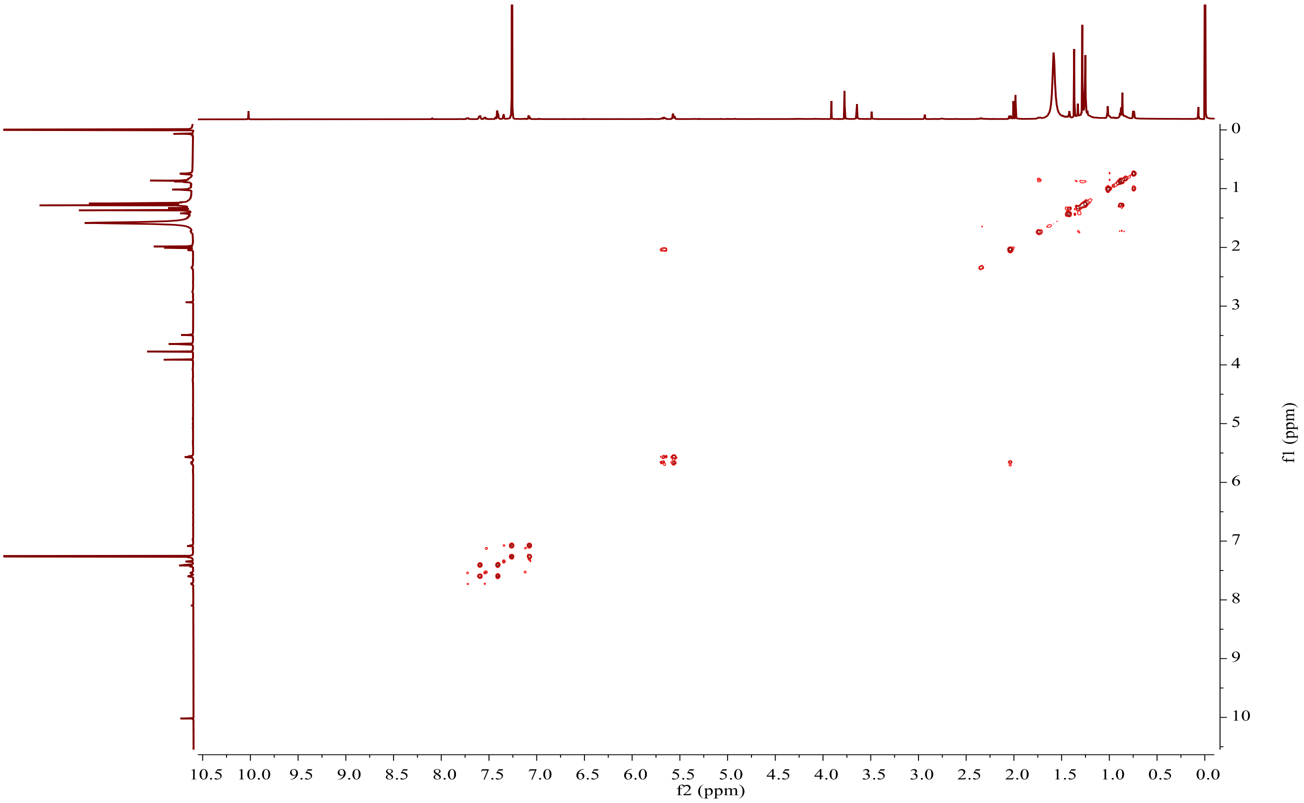

HRESIMS

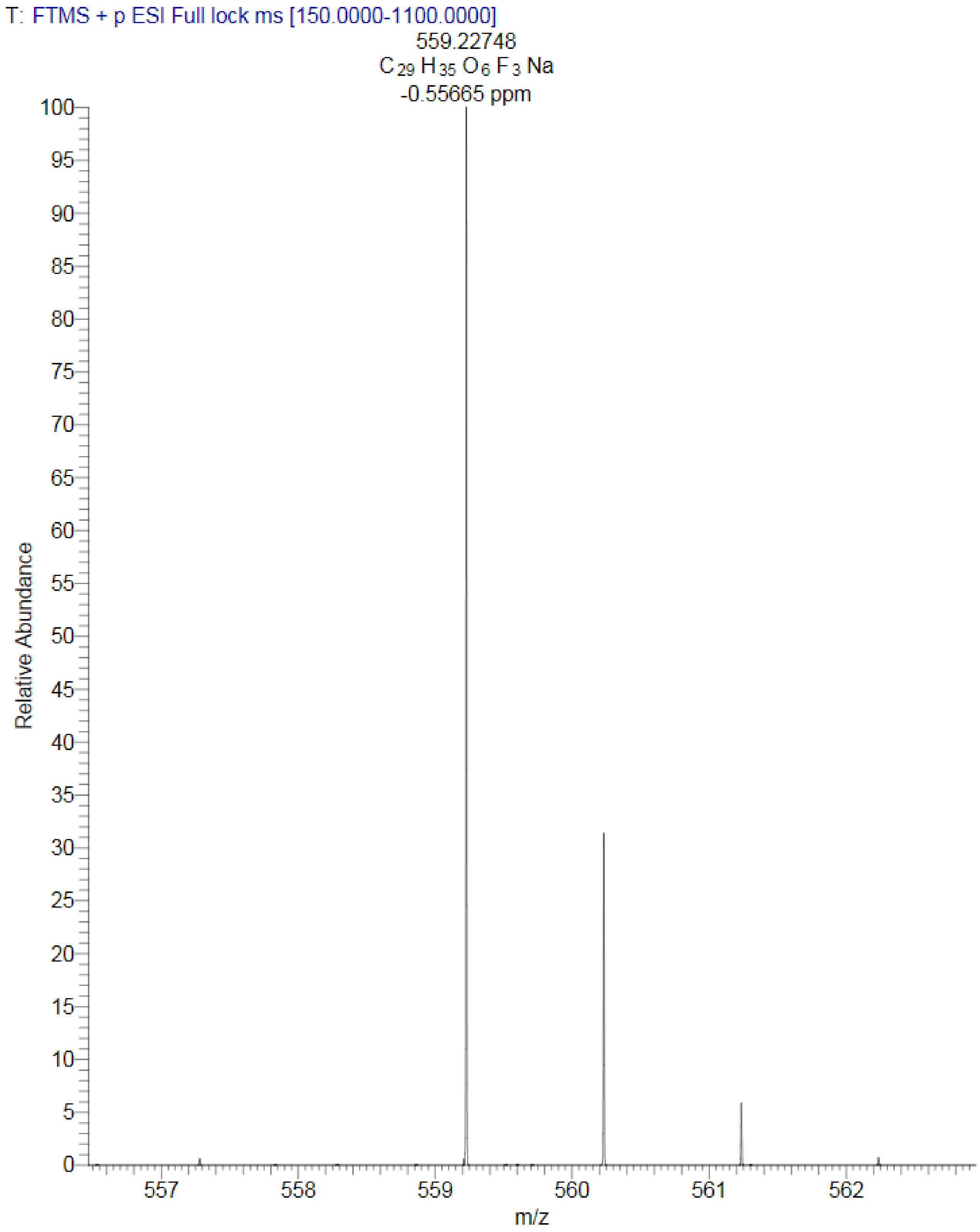

T: FTMS + p ESI Full lock ms [150.0000-1100.0000]
559.22748
C 29 H 35 O 6 F 3 Na
-0.55665 ppm
Relative Abundance
m/z

S1.11 HPLC analysis of ethanol extract of *Bipolaris* sp.

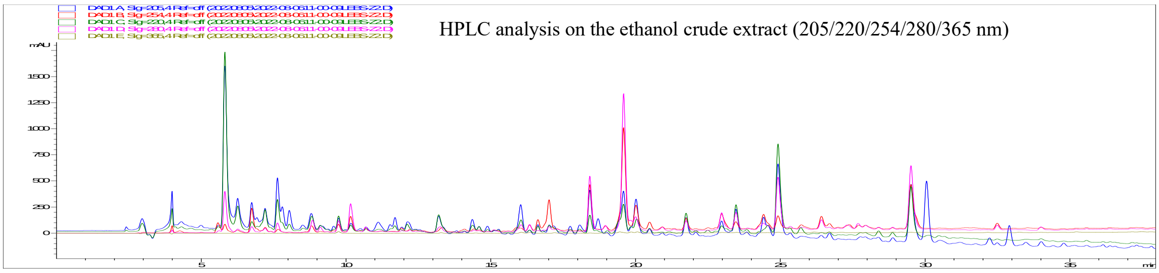

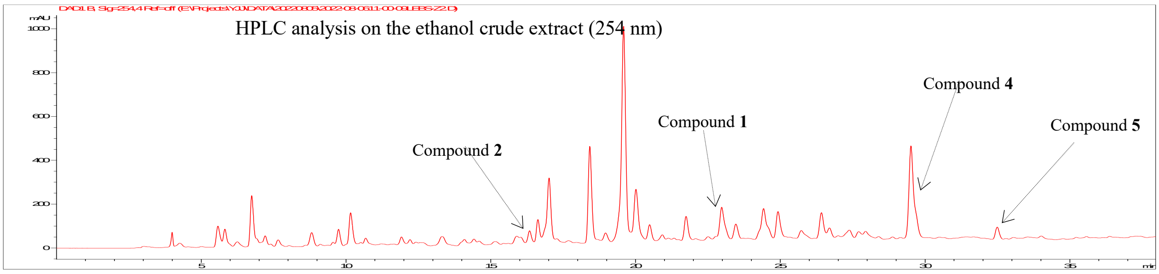

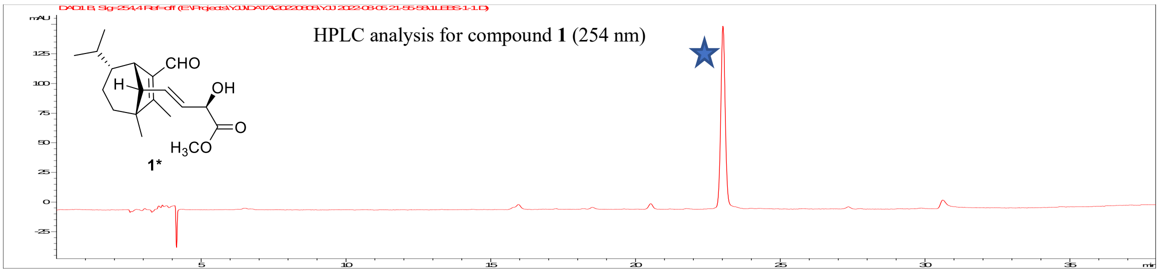

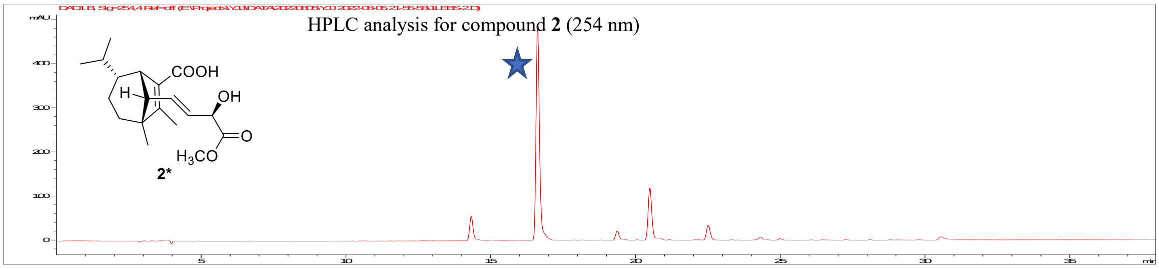

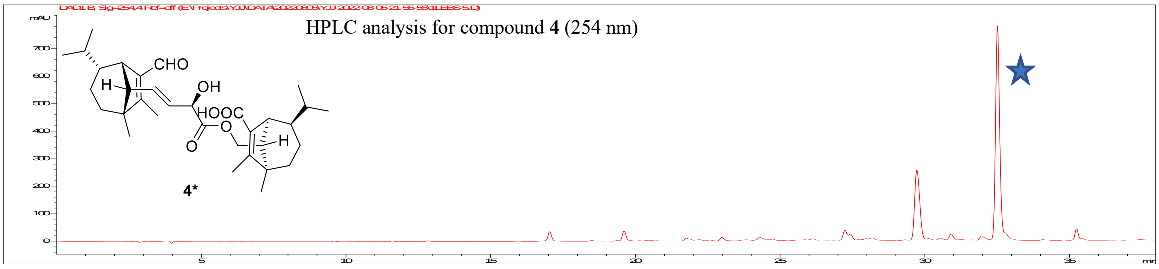

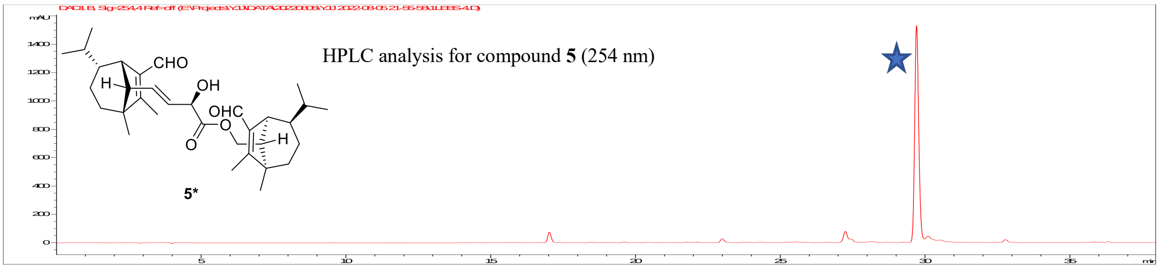

Sections S2. Computational details

S2.1 Computational details for bipolariterpene B (NMR)

All the optimized conformers were subjected to Gauge Independent Atomic Orbital (GIAO) calculations of their ^{13}C NMR chemical shifts using density functional theory (DFT) at the mPW1PW91/6-311+G (d, p) level with the PCM model in methanol. The calculated NMR data of these conformers were averaged according to the Boltzmann distribution theory and their relative Gibbs free energy. The ^{13}C NMR chemical shifts for TMS were also calculated by the same procedures and used as the reference. After calculation, the experimental and calculated data were evaluated by the improved probability DP4+ method.

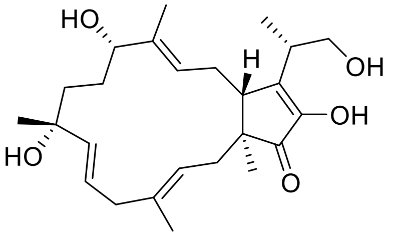

7A

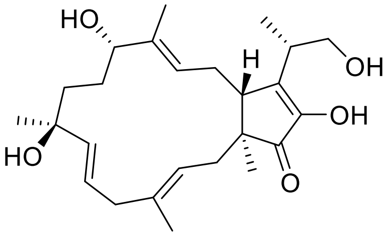

7B

Table S1. Energy analysis for conformers of **7Aa~7Ag** at B3LYP/6-31G(d) level in the gas phase

Species	*E′=E+ZPE*	*E*	*H*	*G*	*ΔG*	*ΔE*(kcal/mol)	*PE*%
**7Aa**	-1351.137332	-1351.105592	-1351.104648	-1351.197889	0	0	77.71%
**7Ab**	-1351.13514	-1351.102909	-1351.101964	-1351.196475	0.001414	0.887298433	17.37%
**7Ac**	-1351.132335	-1351.100026	-1351.099082	-1351.194672	0.003217	2.018698061	2.57%
**7Ad**	-1351.131629	-1351.09894	-1351.097996	-1351.19414	0.003749	2.352533115	1.46%
**7Ae**	-1351.132731	-1351.100829	-1351.099884	-1351.193507	0.004382	2.749746629	0.75%
**7Af**	-1351.126941	-1351.093762	-1351.092818	-1351.190052	0.007837	4.917791952	0.02%
**7Ag**	-1351.129085	-1351.096348	-1351.095404	-1351.191807	0.006082	3.816512779	0.12%

E, *E′*, *H*, *G*: total energy, total energy with zero point energy (*ZPE*), enthalpy, and Gibbs free energy

Figure S1. Main conformers of **7A**

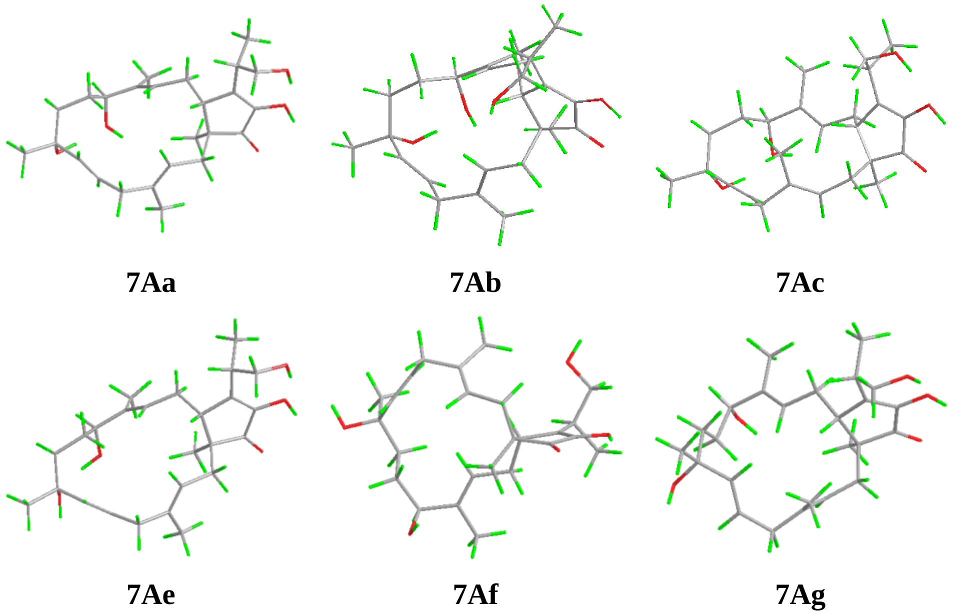

Table S2. Energy analysis for conformers of **7Ba~7Bh** at B3LYP/6-31G(d) level in the gas phase

Species	*E'*=*E*+*ZPE*	*E*	*H*	*G*	*ΔG*	*ΔE*(kcal/mol)	*PE*%
**7Ba**	-1351.133341	-1351.100865	-1351.099921	-1351.196268	0.000199	0.124874391	31.9%
**7Bb**	-1351.134858	-1351.102796	-1351.101852	-1351.196467	0	0	39.4%
**7Bc**	-1351.131825	-1351.098969	-1351.098025	-1351.194277	0.00219	1.374245805	3.9%
**7Bd**	-1351.133676	-1351.101214	-1351.10027	-1351.195275	0.001192	0.747991324	11.1%
**7Be**	-1351.13147	-1351.098896	-1351.097952	-1351.193797	0.00267	1.675450365	2.3%
**7Bf**	-1351.131997	-1351.099259	-1351.098315	-1351.194476	0.001991	1.249371415	4.8%
**7Bg**	-1351.131739	-1351.098948	-1351.098004	-1351.193533	0.002934	1.841112873	1.8%
**7Bh**	-1351.131997	-1351.099259	-1351.098315	-1351.194475	0.001992	1.249998924	4.8%

E, *E′*, *H*, *G*: total energy, total energy with zero point energy (*ZPE*), enthalpy, and Gibbs free energy

Figure S2. Main conformers of **7B**

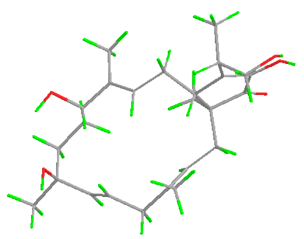

7Ba

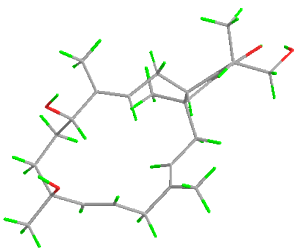

7Bb

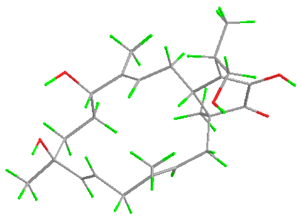

7Bc

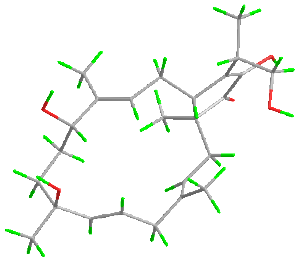

7Bd

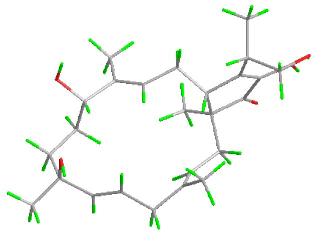

7Be

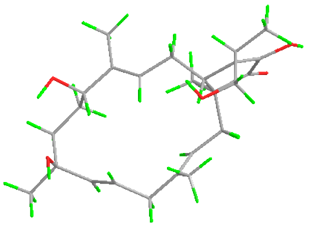

7Bf

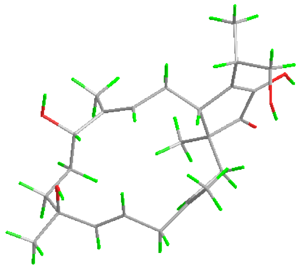

7Bg

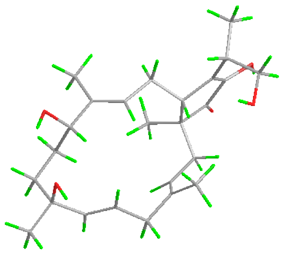

7Bh

Figure S3. ^{13}C NMR calculation results of two possible isomers of **7**

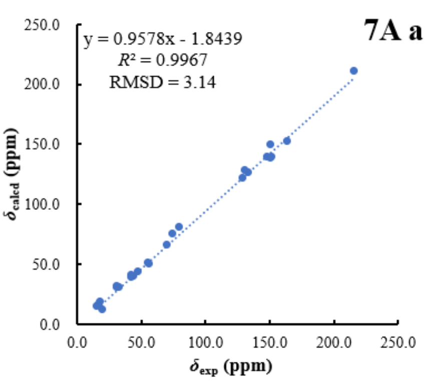

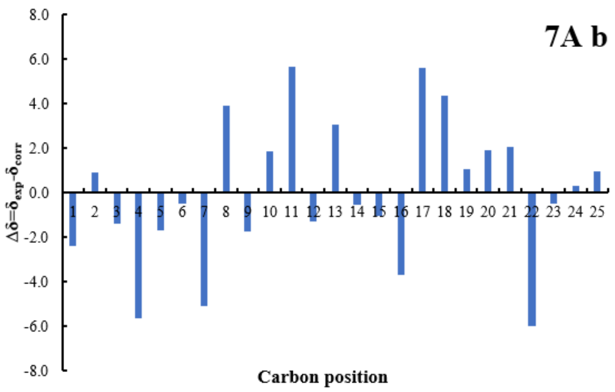

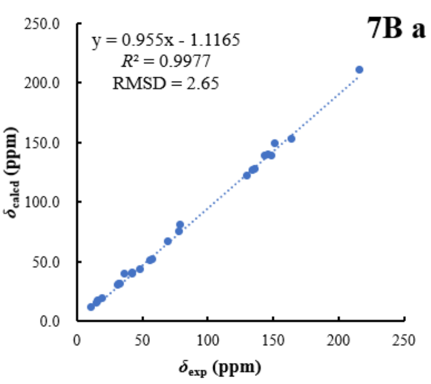

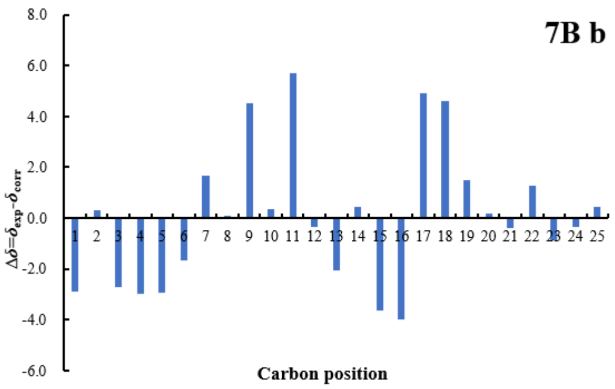

a: Linear correlation plots of predicted versus experimental ^{13}C NMR chemical shift. **b**: Relative errors between the predicted δ_C of two potential structures and recorded δ_C.

Table S3. DP4+ analysis results of **7A** (Isomer 1) and **7B** (Isomer 2)

mPW1PW91		PCM		6-311+G(d,p)	Shielding Tensors		
		DP4+	0.00%	100.00%	-	-	-
Nuclei	sp2?	Experimental	Isomer 1	Isomer 2	Isomer 3	Isomer 4	Isomer 5
C		49.8	131.8	131.8			
C		39.9	145.6	145.5			
C	x	120.9	58.6	57.5			
C	x	138.4	35.9	38.9			
C		42.9	139.7	139.0			
C	x	126.1	54.1	53.2			
C	x	138	36.9	44.2			
C		74.3	112.8	109.3			
C		39	143.7	150.9			
C		30.6	156.3	155.3			
C		80.5	108.1	108.7			
C	x	139.1	39.7	41.0			
C	x	127.4	56.4	51.4			
C		30	154.4	156.0			
C		50.9	132.0	129.9			
C	x	152.2	23.5	23.4			
C	x	148.8	36.8	36.3			
C	x	210.1	-28.5	-28.2			
C		16.5	170.1	171.2			
C		18	169.4	168.3			
C		30.4	156.7	154.7			
C		11.4	168.1	176.4			
C		38.8	145.2	145.4			
C		65.8	117.9	117.7			
C		14.6	172.0	172.2			

Functional	Solvent?		Basis Set		Type of Data	
mPW1PW91	PCM		6-311+G(d,p)		Shielding Tensors	
	Isomer 1	Isomer 2	Isomer 3	Isomer 4	Isomer 5	Isomer 6
sDP4+ (H data)	-	-	-	-	-	-
sDP4+ (C data)	0.01%	99.99%	-	-	-	-
sDP4+ (all data)	0.01%	99.99%	-	-	-	-
uDP4+ (H data)	-	-	-	-	-	-
uDP4+ (C data)	8.64%	91.36%	-	-	-	-
uDP4+ (all data)	8.64%	91.36%	-	-	-	-
DP4+ (H data)	-	-	-	-	-	-
DP4+ (C data)	0.00%	100.00%	-	-	-	-
DP4+ (all data)	0.00%	100.00%	-	-	-	-

S2.3 Computational details for bipolariterpene C (NMR)

All the optimized conformers were subjected to Gauge Independent Atomic Orbital (GIAO) calculations of their ^{13}C NMR chemical shifts using density functional theory (DFT) at the mPW1PW91/6-311+G (d, p) level with the PCM model in chloroform. The calculated NMR data of these conformers were averaged according to the Boltzmann distribution theory and their relative Gibbs free energy. The ^{13}C NMR chemical shifts for TMS were also calculated by the same procedures and used as the reference. After calculation, the experimental and calculated data were evaluated by the improved probability DP4+ method.

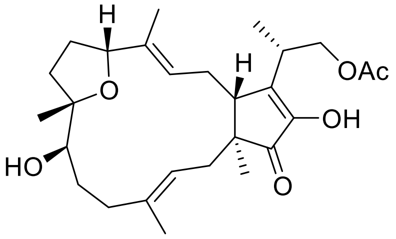

8A

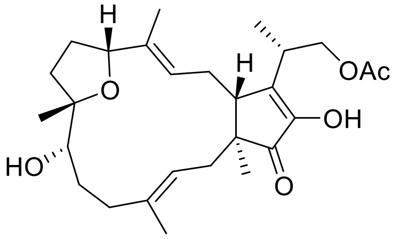

8B

Table S4. Energy analysis for conformers of **8Aa~8Ak** at B3LYP/6-31G(d) level in the gas phase

Species	*E′=E+ZPE*	*E*	*H*	*G*	*ΔG*	*ΔE*(kcal/mol)	*PE*%
**8Aa**	-1503.799376	-1503.764365	-1503.763421	-1503.865664	0	0	20.00%
**8Ab**	-1503.799492	-1503.764605	-1503.763661	-1503.864905	0.000759	0.248579711	13.14%
**8Ac**	-1503.799146	-1503.764341	-1503.763397	-1503.864896	0.000768	0.251527296	13.07%
**8Ad**	-1503.799137	-1503.765169	-1503.764225	-1503.862013	0.003651	1.195737184	2.65%
**8Ae**	-1503.797536	-1503.762939	-1503.761995	-1503.861947	0.003717	1.217352811	2.56%
**8Af**	-1503.797055	-1503.761907	-1503.760963	-1503.864393	0.001271	0.416264574	9.90%
**8Ag**	-1503.797894	-1503.762784	-1503.761839	-1503.864855	0.000809	0.264955185	12.78%
**8Ah**	-1503.795201	-1503.761441	-1503.760496	-1503.857598	0.008066	2.641691627	0.23%
**8Ai**	-1503.799455	-1503.764801	-1503.763857	-1503.865191	0.000473	0.154911993	15.39%
**8Aj**	-1503.796094	-1503.761268	-1503.760324	-1503.862301	0.003363	1.101414449	3.11%
**8Ak**	-1503.799553	-1503.765046	-1503.764102	-1503.863808	0.001856	0.607857632	7.16%

E, *E′*, *H*, *G*: total energy, total energy with zero point energy (*ZPE*), enthalpy, and Gibbs free energy

Figure S4. Main conformers of **8A**

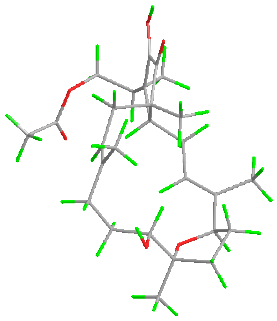

8Aa

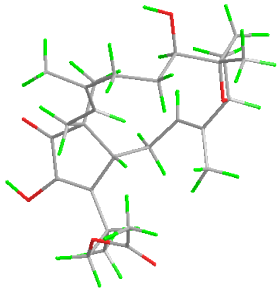

8Ab

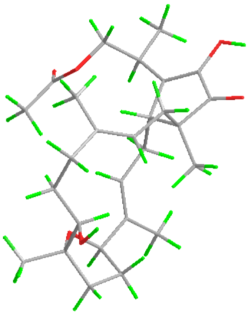

8Ac

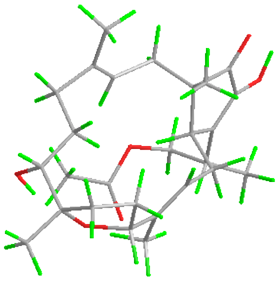

8Ad

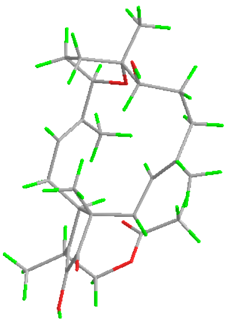

8Ae

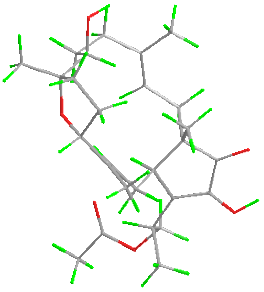

8Af

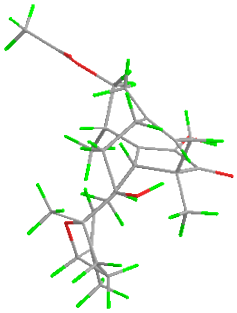

8Ag

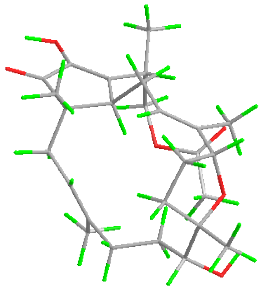

8Ah

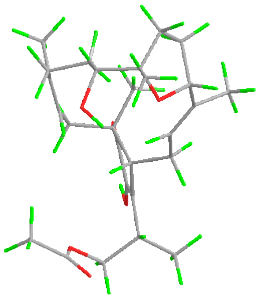

8Ai

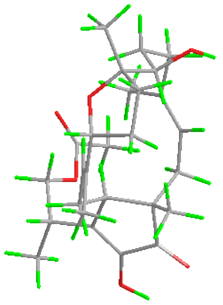

8Aj

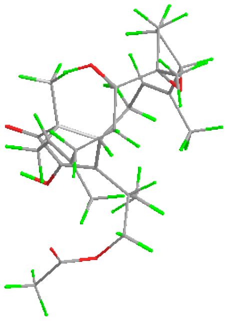

8Ak

Table S5. Energy analysis for conformers of **8Ba~8Be** at B3LYP/6-31G(d) level in the gas phase

Species	*E′*=*E*+*ZPE*	*E*	*H*	*G*	*ΔG*	*ΔE*(kcal/mol)	*PE*%
**8Ba**	-1503.807343	-1503.773395	-1503.772451	-1503.870315	0.000766	0.480672277	17.26%
**8Bb**	-1503.807589	-1503.773518	-1503.772574	-1503.871081	0	0	38.87%
**8Bc**	-1503.804675	-1503.770038	-1503.769094	-1503.870425	0.000656	0.411646232	19.39%
**8Bd**	-1503.805271	-1503.770707	-1503.769763	-1503.870622	0.000459	0.288026861	23.89%
**8Be**	-1503.805558	-1503.772001	-1503.771057	-1503.86713	0.003951	2.479290034	0.59%

E, *E′*, *H*, *G*: total energy, total energy with zero point energy (*ZPE*), enthalpy, and Gibbs free energy

Figure S5. Main conformers of **8B**

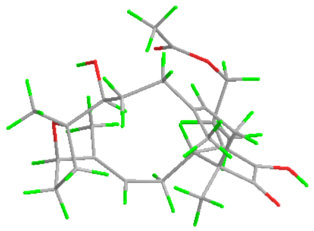

8Ba

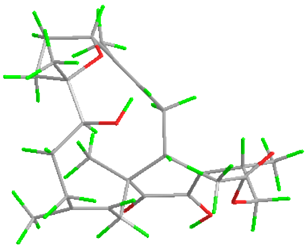

8Bb

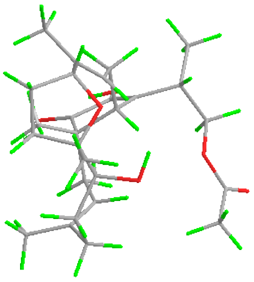

8Bc

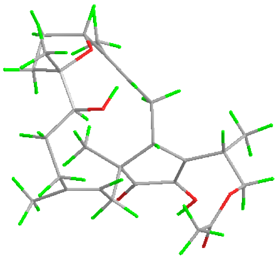

8Bd

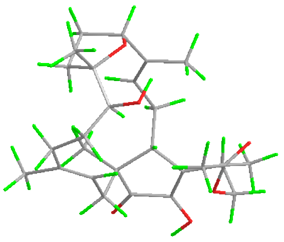

8Be

Figure S6. ^{13}C NMR calculation results of two possible isomers of **8**

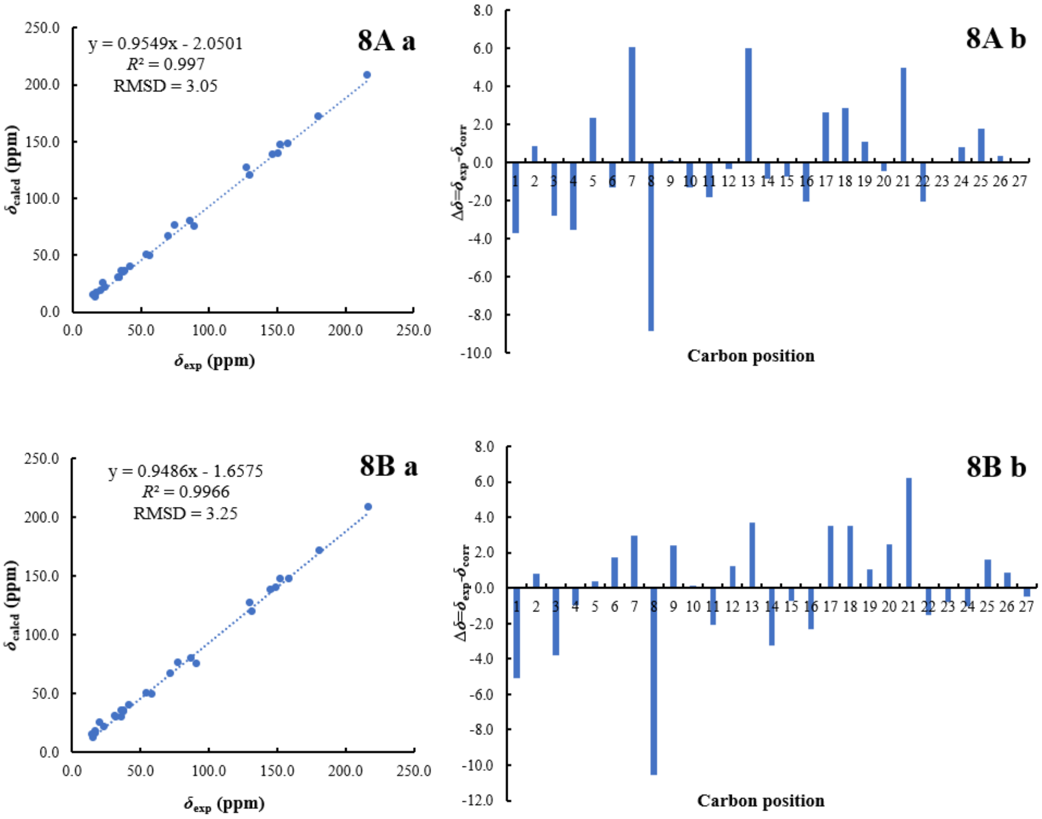

a: Linear correlation plots of predicted versus experimental ^{13}C NMR chemical shift. **b**: Relative errors between the predicted δ_C of two potential structures and recorded δ_C.

Table S6. DP4+ analysis results of **8A** (Isomer 1) and **8B** (Isomer 2)

Functional	Solvent?	Basis Set	Type of Data
mPW1PW91	PCM	6-311+G(d,p)	Shielding Tensors

		DP4+	100.00%	0.00%	-	-	-
Nuclei	sp2?	xperimenta	Isomer 1	Isomer 2	Isomer 3	Isomer 4	Isomer 5
C		49	130.6	129.4			
C		39.3	145.9	145.8			
C	x	119.6	57.3	56.3			
C	x	139.2	36.9	38.6			
C		35.3	150.6	149.6			
C		30.1	152.8	156.5			
C		75.7	112.4	109.7			
C		74.8	98.0	96.4			
C		35.3	148.8	151.7			
C		29.5	154.7	155.5			
C		79.1	101.4	100.8			
C	x	138.1	40.5	42.1			
C	x	126.2	59.8	57.2			
C		29.6	154.2	151.8			
C		49.8	133.4	133.1			
C	x	147.4	30.4	28.5			
C	x	146.6	34.7	35.5			
C	x	207.8	-28.7	-29.0			
C		16	170.5	170.6			
C		17.8	167.6	170.2			
C		24.6	165.6	167.0			
C		12.3	170.3	171.8			
C		34.1	149.9	149.6			
C		66.5	117.3	115.2			
C		14.7	172.0	172.6			
C	x	171.1	7.13	6.94			
C		21	163.88	163.72			

Functional	Solvent?	Basis Set	Type of Data
mPW1PW91	PCM	6-311+G(d,p)	Shielding Tensors

	Isomer 1	Isomer 2	Isomer 3	Isomer 4	Isomer 5	Isomer 6
sDP4+ (H data)	-	-	-	-	-	-
sDP4+ (C data)	99.61%	0.39%	-	-	-	-
sDP4+ (all data)	99.61%	0.39%	-	-	-	-
uDP4+ (H data)	-	-	-	-	-	-
uDP4+ (C data)	99.98%	0.02%	-	-	-	-
uDP4+ (all data)	99.98%	0.02%	-	-	-	-
DP4+ (H data)	-	-	-	-	-	-
DP4+ (C data)	100.00%	0.00%	-	-	-	-
DP4+ (all data)	100.00%	0.00%	-	-	-	-
